# Two Frequenins in *Drosophila*: unveiling the evolutionary history of an unusual Neuronal Calcium Sensor (NCS) duplication

**DOI:** 10.1186/1471-2148-10-54

**Published:** 2010-02-19

**Authors:** Alejandro Sánchez-Gracia, Jesús Romero-Pozuelo, Alberto Ferrús

**Affiliations:** 1 Instituto Cajal, CSIC, Ave. Dr. Arce 37, Madrid 28002, Spain; 2 Institute of Evolutionary Biology, IBE (CSIC-UPF), Passeig Marítim de la Barceloneta, 37-49, 28003 Barcelona, Spain; 3 Centro de Biología Molecular, UAM/CSIC, c/Nicolas Cabrera 1 Madrid 28049, Spain

## Abstract

**Background:**

*Drosophila *Frequenin (Frq), the homolog of the mammalian Neuronal Calcium Sensor-1 (NCS-1), is a high affinity calcium-binding protein with ubiquitous expression in the nervous system. This protein has an important role in the regulation of neurotransmitter release per synapse, axonal growth and bouton formation. In *D. melanogaster*, Frequenin is encoded by two genes (*frq1 *and *frq2*), a very unexpected feature in the Frq/NCS-1 subfamily. These genes are located in tandem in the same genomic region, and their products are 95% identical in their amino acid sequence, clearly indicating their recent origin by gene duplication. Here, we have investigated the factors involved in this unusual feature by examining the molecular evolution of the two *frq *genes in *Drosophila *and the evolutionary dynamics of NCS family in a large set of bilaterian species.

**Results:**

Surprisingly, we have found no amino acid replacements fixed across the twelve *Drosophila *species surveyed. In contrast, synonymous substitutions have been prevalent in the evolution of the coding region of *frq1 *and *frq2*, indicating the presence of strong functional constraints following gene duplication. Despite that, we have detected that significant evolutionary rate acceleration had occurred in Frq1 in early times from the duplication, in which positive selection (likely promoting functional diversification) had probably an important role. The analysis of sequence conservation and DNA topology at the non-coding regions of both genes has allowed the identification of DNA regions candidates to be *cis*-regulatory elements. The results reveal a possible mechanism of regulatory diversification between *frq1 *and *frq2*.

**Conclusions:**

The presence of two Frequenins in *Drosophila *and the rapid accumulation of amino acid substitutions after gene duplication are very unusual features in the evolution of the Frq/NCS-1 subfamily. Here we show that the action of positive selection in concordance with some extent of regulatory diversification might explain these findings. Selected amino acid substitutions in Frq1 likely contributed to the functional divergence between the two duplicates, which, in turn, should have diverged in their regulation by Ecdysone-induced early genes.

## Background

Many different aspects of neuronal function, including neurotransmitter release from synaptic vesicles, are regulated by alterations in the concentration of intracellular free Ca^2+ ^[1,2]. The transduction of calcium signals into appropriate physiological responses is frequently mediated by a range of calcium sensor proteins, which act as effectors and modulators in signalling pathways [3]. The specific effects of these changes depend on their affinity for Ca^2+^, their cellular localization in relation to the Ca^2+ ^entry signal and their interaction with other proteins. A number of calcium binding proteins related to the ubiquitous protein Calmodulin are overrepresented in or expressed only in the nervous system. These include the family of intracellular Neuronal Calcium Sensor (NCS) proteins [4-6]. Members of this protein family, which have been identified in many organisms ranging from yeast to mammals, bind Ca^2+ ^at their EF-hand domains and regulate many important processes in neuronal signalling. This family can be divided into five subfamilies or classes (A-E) based on their amino acid sequence similarity [3].

*Drosophila *Frequenin (Frq), the homolog of the mammalian Neuronal Calcium Sensor-1 (NCS-1), was the founder member of the NCS family and the first to be characterized being, at this time, the only component of the class A [7]. Frequenin is a high affinity calcium-binding protein composed by two pairs of EF-hand motifs with ubiquitous expression in the nervous system. Its over-expression enhances facilitation and increases release per synapse [8,9]. Also, the levels of Frq in motoneuron control the number of boutons and branching, strongly suggesting a role in modulating axonal growth and bouton formation [8,9]. In *Drosophila melanogaster*, this calcium sensor is encoded by two different genes, *frq1 *and *frq2 *[9], which are located in tandem in the same genomic region (spaced by 11.6 kb). These two genes show a similar intron-exon structure and a high amino acid sequence similarity (they differ in only 10 residues [9]). All these features clearly support an origin by gene duplication and suggest that the encoded proteins would still perform similar functions in *D. melanogaster*. In addition, the prospective search [7] across some fully sequenced genomes failed to detect duplicated copies belonging to this subfamily, with the exception of the zebrafish *Danio rerio *[10]. In this species, like in *Drosophila*, there are two independent Frequenin encoding genes. In *D. melanogaster*, both genes have very similar temporal and qualitative expression profiles but their quantitative levels differ [9]: while *frq1 *mRNA is 2-3 fold more abundant at the end of the embryo stage and in 1st instar larvae than in the adult, *frq2 *mRNA always reaches its peak expression level in the adult. Comparing the mRNA expression in the adult, *frq*1 shows a 20-30% higher level than *frq*2. Significant expression in the ventral ganglia was found for both genes, and this expression appears to be specific. Interestingly, the reduction or abolishment of Frequenin activity in flies does produce neither lethality nor sterility, which originally suggested a small contribution to individual fitness of these proteins. The analyses of over-expression and loss-of-function phenotypes, nevertheless, showed that mutants had larval locomotion defects, deficient synaptic transmission, impaired Ca^2+ ^entry and enhanced nerve terminal growth, demonstrating a relevant role of Frequenin in the development and function of the nervous system [9,11].

In order to understand the causes of the atypical preservation of two *frq *genes in *Drosophila*, we have analyzed nucleotide and amino acid sequence variation of *frq1 *and *frq2 *across the twelve species of the *Drosophila *genus with the complete genome sequenced [12]. Besides, we have also examined amino acid sequence evolution of the other members of NCS protein family to identify functionally important sites responsible for differentiation between subfamilies. We have found that positive selection had an important role in the fixation of some of the amino acid changes found between *Drosophila *Frequenins. The results also show that these changes probably shaped the functional differentiation between the two copies. Finally, the evolutionary analysis of non-coding sequences suggests that gene expression differences observed between these two genes might be, at least in part, consequence of the different evolution of regulatory regions after gene duplication.

## Results

### Two old and highly constrained Frequenins in *Drosophila*

**Figure 1:**
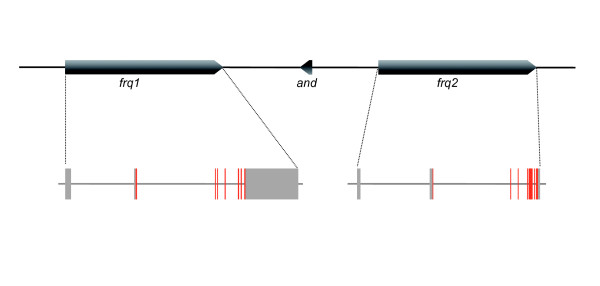
**Genomic structure of *frq1 *and *frq2 *genes in *Drosophila melanogaster***. Schematic representation of the genomic region encompassing *frq1 *and *frq2 *in the *Drosophila melanogaser *X chromosome. Red and grey boxes indicate coding and non-conding exons, respectively. Arrow head indicates direction of transcription with respect to the centromere which is at the right. The small gene *Andorra *(*and*) is located between the two *frq *genes.

We have found that the 12 *Drosophila *species have two genes encoding Frequenin. In all species, the orthologues of *frq1 *and *frq2 *are located in the syntenic region of the X chromosome and maintain nearly the same gene structure (i.e. exon/intron number and sizes; Figure 1). The *frq1 *genes of *D. sechellia *and *D. pseudoobscura *are situated in genomic contigs that contain several gaps filled with 'N' and they are not complete. In spite of that, partial coding regions available for these genes have no mutations truncating the open reading frames or generating stop codons, suggesting that both are functional copies. In order to be conservative in our further analysis, however, we used only complete coding regions for comparisons.

**Figure 2:**
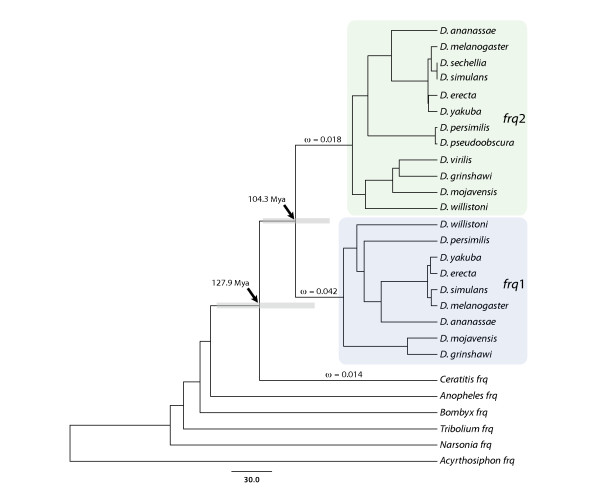
**Phylogenetic tree of insect *frq *homologuous genes**. Bayesian phylogeny of insect homologues based on substitutions at synonymous positions. Arrows indicate the age of the Frq1-Frq2 duplication and of the common ancestor of *Drosophila *and *Ceratitis *Frequenins. The 95% high posterior density intervals of node ages are depicted in grey. Coloured shaded boxes denote the *frq1 *and *frq2 *subtrees. The ω values estimated in the ML analysis are also shown. Scale is in My.

Both genes show high levels of silent nucleotide divergence across the *Drosophila *genus. The Maximum Likelihood (ML) estimate of the total tree length (using PAML software) for synonymous substitutions per synonymous site, *d*_s_, is 1.96 and 2.98, for *frq*1 and *frq*2, respectively. As expected from an independent evolution after gene duplication, the ML average estimate of synonymous divergence between paralogues (*d*_s _= 1.78) is higher than across orthologues (*frq1*, *d*_s _= 0.58; *frq2*, *d*_s _= 1.07), with little evidence for concerted evolution (based on a gene conversion analysis; data not shown). Surprisingly, despite the extensive divergence at synonymous sites, no nonsynonymous substitution has been detected across orthologous copies of neither *frq1 *nor *frq2*. In fact, non-synonymous divergence is exclusively restricted to between duplicates (*d*_N _= 0.036), resulting in the 10 fixed amino acid differences between Frq1 and Frq2 proteins previously detected in [9]. Furthermore, estimates of silent variation suggest that *frq*1 and *frq*2 have evolved at different evolutionary rate across the *Drosophila *genus since results of the two-cluster test confirm that *frq2 *accumulates synonymous substitutions faster than its paralogue (two-cluster test in LINTRE, *P*-value = 0.007). Using BEAST, we have estimated the age of duplication in 104 My (95% posterior density: 78-135 My; Figure 2), which implies that the two copies originated at some point before the split of the *Drosophila *and *Sophophora *subgenera (~60 Mya).

### Accelerated evolution of Frq1 in early stages after gene duplication

**Figure 3:**
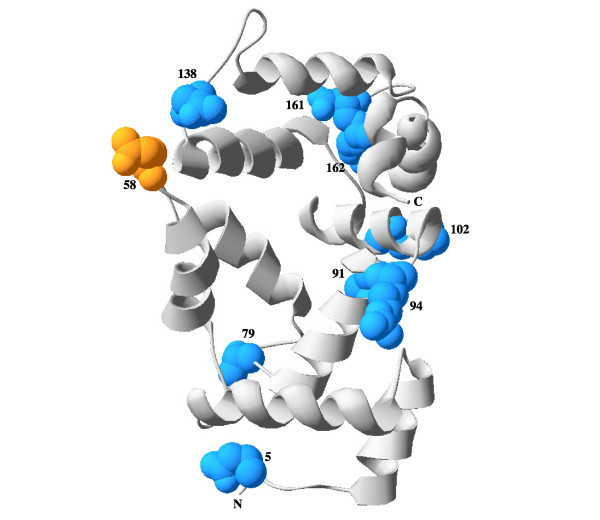
**Putative structure of the ancestral Frequenin**. Predicted 3D structure of the ancestral Frequenin. The model is based on amino acid sequence reconstructed from the comparison of known Frequenins in insects (sequences in figure 2). Amino acid replacements found in *D. melanogaster *Frq1 and Frq2 are highlighted in blue and orange, respectively. Numbers indicate the position in the Frq1. C and N termini are indicated.

In contrast to *Drosophila*, the other surveyed insect species hold only one *frq *gene (Figure 2). All these single insect homologues are co-orthologues of *Drosophila frq1 *and *frq2 *(i.e., they are orthologues of both *Drosophila *copies), likely indicating that the duplication postdated the split between *Drosophila *and *Ceratitis *relatives. The putative sequence of the ancestral Frequenin, reconstructed by a ML approach (with PAML software) using the JTT distance [13] (which correspond to the best-fit amino acid substitution model in the model selection analysis), was used to polarize the 10 replacements fixed between duplicates; interestingly, nine of them occurred in the Frq1 lineage (only the substitution in the position 58 seems to have occurred in Frq2; amino acid positions are numbered relative to the sequence of Frq1, a convention used throughout this report). In fact, amino-acid substitution rate is significantly different between paralogues after duplication (using *Ceratitis capitata *Frequenin as reference and the JTT distance; Relative Rate Test module in Hyphy package, *P*-value = 0.05), which is consistent with an acceleration of amino-acid substitution rate in Frq1. Replacements are broadly distributed along the primary structure of Frq1 with three of them included within EF-hand domains. Figure 3 shows the location of the residues with nonsynonymous substitutions in the modelled 3D structure. There is no apparent spatial coupling among residues, except for the two pairs that are contiguous (161 and 162) or very close (91 and 94) in the primary structure. We have also investigated the relationship between amino acid substitutions and residue accessibility. We have found that five residues (positions 5, 58, 94, 102 and 138) should be exposed in the Frequenin ancestral protein whereas the rest (79, 91, 161 and 162) were probably less accessible. Thus, in contrast to the observations for other proteins [14], solvent exposure does not seem to be a major structural determinant of *Drosophila *Frequenins residue evolution.

**Figure 4:**
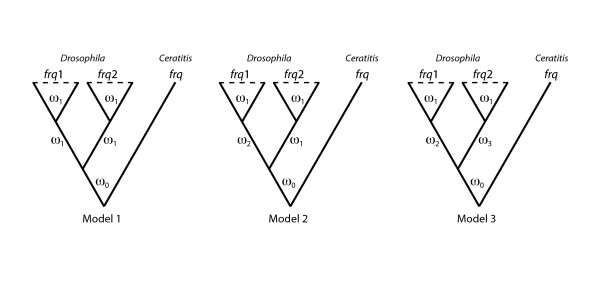
**Evolutionary models used in the ML analysis**. Scheme of the evolutionary models compared in the codon model substitution-based ML analysis. ω_0_, ω_1_, ω_3 _and ω_3 _denote different *d*_N_/*d*_S _ratios. Model 1 (null model), Model 2 (alternative model 1) and Model 3 (alternative model 2) have 44, 45 and 46 free parameters (the degrees of freedom for each LRT is equal to the difference in the number of free parameters), respectively.

To determine whether functional constraints changed after gene duplication we have performed a ML analysis using the codon substitution models implemented in PAML software (Figure 4). The evolutionary scenario allowing different nonsynonymous to synonymous substitution rates ratio, ω, in the internal branches leading to the *frq*1 and *frq2 *lineages (Model 3) clearly shows the best-fit to the data (Model 2 versus Model 1, *P*-value = 1.92 x 10^-7^; Model 3 versus Model 2, *P*-value = 0.0002). This result implies that selective constraints acting on the two copies changed both immediately after the duplication event and some time before the split of the *Drosophila *genus species. ML estimates of the ω parameter in these lineages (Figure 2) advocate for a relaxation of functional constraints acting on *frq1 *gene just after gene duplication or, alternatively, for the action of positive selection driving some of the replacements found in the protein encoded by this gene.

### Divergence of non-coding *frq1 *and *frq2 *sequences

The evolutionary fate of duplicated copies can be also determined by changes in non-coding regions (i.e. regulatory divergence). We have analyzed nucleotide divergence at non-coding sequences of *frq*1 and *frq*2 in six species of the *Sophophora *genus. The Dot Plot of the complete genomic region containing both genes (Additional file 1) clearly shows coding exons as the only regions with significant sequence similarity between both genes, i.e. intron and flanking non-coding sequences do not retain signals of the duplication event. In contrast, the phylogenetic shadowing analysis using PhastCons reveals a number of significantly conserved non-coding sequences (CNS) across species, which might indicate the presence of functional regulatory elements (Additional file 2). Interestingly, some of these CNS are located in the two large introns of these genes (see Figure 1), which might have been maintained across *Drosophila *genus because of their functional role. Among these CNS, we have identified a number of topologically highly accessible transcription factor binding (TF) motives that could be involved in the regulation of *frq1 *and *frq2 *in these species (Additional file 2). Interestingly, the motives for two factors, DL-2 (dorsal-2) and Brc (Broad-complex) are only present in *frq1 *whereas binding sites for Eip74EF (Ecdysone induced protein 74EF) appear exclusively in *frq2*. Although a few species have putative binding sites for Brc and Eip74EF factors in one duplicate, in all cases only one of the sites maintains its position across the subgenus. Apart from these differentially distributed TF binding sites, the regulatory regions of *frq *genes have also highly accessible conserved sites for Ubx (Ultrabitorax), HB (Hunchback), Sna (Snail) and HSP-1 (Heat Shock Protein 1). In addition, we have found a number of accessible CNS with no apparent TF binding site present in surveyed databases, which might represent regulatory regions for other factors not yet identified.

### Amino acid sequence evolution in the NCS family

In order to better understand the evolutionary history of Frequenin in *Drosophila*, we have also analyzed protein sequence evolution of this subfamily in a large set of bilaterian species. The phylogenetic analysis (Multiple Sequence Alignment (MSA) with the 38 Frequenins with complete sequence) shows that Frq/NCS-1 is a strongly conserved protein - the mean expected number of amino acid substitutions per amino acid site is 1.037. The most variable positions are outside the EF-hand domains, which are well conserved. The evolutionary rate of positions with changes between *Drosophila *Frequenins is variable, some of them being among the most conserved positions (Additional file 3). Results of the exhaustive search for homologous copies in databases (we found homologues of Frequeninin more than 50 bilaterian species, with representatives in major taxonomic groups) confirm that duplications are rare in the evolution of this subfamily; in fact, no fixed duplication has been found other than the already known in *Danio rerio *[10]. The two zebrafish copies (*ncs1a *and *ncs1b*) are noticeably younger than *frq*1 and *frq*2, i.e. the closest species in our data set with only one Frequenin is *Pimephales pomelas*, which diverged from *Danio rerio *20-50 Mya [15,16]; NCS1a and NCS1b differ in 7 amino acid positions, but unlike in *Drosophila*, changes have been homogeneously distributed between paralogues after gene duplication (using the sequence of *P. pomelas *as reference, 3 and 3 changes in NCS1a and NCS1b, respectively, and one ambiguous replacement).

To evaluate the possibility that amino acid substitutions between Frq1 and Frq2 were correlated, we have searched for the presence of coevolving positions across the Frq/NCS-1 subfamily MSA. The analysis based on correlation coefficients [17] does not detect any significant intra-molecular co-evolution across bilaterian Frequenins. In contrast, the CTMP model approach [18], allows detecting 40 pairs of amino acid sites with significantly more double substitutions than expected under the best-fit amino acid substitution model. The predicted positions are scattered across the sequence and only the C-terminal is free of putative co-evolving positions. No coordinating residues of the EF-hands appear among the sites with high *score*, with the only exception of position 79, located at the second EF hand. Some of the predicted coevolving pairs enclose positions with amino acid differences between Frq1 and Frq2 proteins (positions 58, 79, 102 and 138 in *Drosophila *Frq1). Nevertheless, one of these positions (position 58) corresponds to the substitution in the Frq2 lineage and no significant pair is composed by two sites with replacements in Frq1.

We have investigated whether amino acid substitutions between Frq1 and Frq2 are actually involved in functional divergence (rather than representing compensatory changes). For that, we extended the analysis to the other NCS subfamilies. The rate of evolution is remarkably different both among subfamilies and between the members of the same NCS class (we analyzed the 14 subfamilies that compose the 5 NCS classes; Table 1 in [6]). NCS-1 and VILIP1 are clearly the most conserved subfamilies (the average root-to-tip length of the vertebrate tree for amino acid substitutions per site is 0.017 and 0.025, respectively) whereas GCAP subfamilies evolve faster than the rest (ranging from 0.297 to 0.739). The results of the functional divergence analysis in Diverge software reveal that positions 58, 79, 91, 102, 138, 161 and 162, have been involved (they are among the 5% highest values in the posterior probability profiles) in functional constraints changes and specific functional divergence between NCS subfamilies (Table 1). These positions show site-specific shifts of evolutionary rate (type-I functional divergence) and/or of amino acid property (type-II functional divergence). Interestingly, although the specific amino acids present in positions 79, 102 and 162 of Frq1 and Frq2 are not the same than those responsible for type II functional divergence between NCS proteins, they also denote radical changes in Frq1 (i.e. in terms of charge and hydrophobicity). This suggests that they might have been involved in cluster specific functional divergence between Frq1 and Frq2 [19]. The same occurs with position 58, which imply a radical change in Frq2. The putative contribution of the positions 5 and 186 to functional divergence between subfamilies was unachievable because of the high levels of divergence in the C- and N-terminal parts of the NCS proteins. The rapid evolution of these regions prevented a confident identification of positional homologies for the two sites.

**Table 1:** Functional divergence between NCS subfamilies

Subfamily (Class)	Subfamily (Class)	Type I θ (SE)	Type II θ (SE)	Positions^a^
Frq/NCS-1 (A)	Recoverin (C)	0.246 (0.089)	0.342 (0.072)	*79*, *102*, 162
	Hippocalcin (B)	0.858 (0.230)	0.273 (0.046)	*79*, 102, *162*
	KChIP1 (E)	0.899 (0.233)	0.415 (0.063)	*58*, *79*, 102, 138
	KChIP2 (E)	NS	0.348 (0.078)	*58*
	KChIP3 (E)	0.594 (0.268)	NS	*138, 162*
	KChIP4 (E)	0.561 (0.232)	0.447 (0.057)	*58*, 138, 162
	VILIP2 (B)	NS	0.298 (0.052)	*102*
	VILIP3 (B)	0.829 (0.285)	-	79, 102
Recoverin (C)	VILIP2 (B)	NS	0.317 (0.076)	*102*
	VILIP3 (B)	0.563 (0.187)	NS	102
	GCAP1 (D)	0.665 (0.069)	0.480 (0.088)	79, *161*
	GCAP2 (D)	0.765 (0.076)	0.501 (0.077)	*161*, 161
	KChIP1 (E)	0.778 (0.090)	NS	91
	KChIP2 (E)	0.867 (0.095)	NS	79
	KChIP3 (E)	0.665 (0.070)	0.547 (0.074)	79, *161*
	KChIP4 (E)	0.731 (0.100)	0.500 (0.070)	79, *161*, *162*
Hippocalcin (B)	KChIP1 (E)	NS	0.455 (0.060)	*58*
	KChIP2 (E)	NS	0.430 (0.073)	*58*
	KChIP4 (E)	NS	0.496 (0.055)	*58*, *162*
VILIP2 (B)	KChIP1 (E)	NS	0.483 (0.060)	*161*
	KChIP2 (E)	NS	0.517 (0.066)	*161*
	KChIP3 (E)	NS	0.416 (0.076)	*161*
	KChIP4 (E)	NS	0.546 (0.055)	*161*
GCAP3 (D)	GCAP2 (D)	NS	0.498 (0.127)	*79*

^a ^Sites with changes between Frq1 and Frq2 that show significant shift in their evolutionary rate between NCS subfamilies (type I and type II sites are in regular and italic font, respectively). Positions are numbered relative to the *D. melanogaster *Frq1 protein. θ, ML estimate of the coefficient of functional divergence. SE, standard error. NS, non-significant.

Among the amino acid positions replaced between Frq1 and Frq2, positions 58, 79, 102, 161 and 162 are the more firm candidates to participate in the functional diversification of the NCS family (Table 1). These five positions are involved in many independent rate shift changes across the evolution of NCS members, although the particular amino acid residues and the type of divergence depend on the comparison. Position 58 contributed to the functional divergence between members of the class E and representatives of the classes A and B. Positions 79 and 102 were involved in several rate shift changes associated with functional divergence between all NCS subfamilies and between families belonging to the classes A, B and C, respectively. Position 161 is a functional diverged site between KChIPs and other subfamilies, whereas the contiguous position, 162, is among the sites with high posterior probability in different comparisons between all NCS classes. Interestingly, these five positions, which involve chemically radical changes between Frq1 and Frq2, also participated (apart from in type I) in type II functional divergence between NCS subfamilies. Finally, positions 91 and 138 (with chemically conservative changes in Frq1) are candidates only for type I functional divergence (between KChIPs and Frequenin and Recoverin, respectively).

## Discussion

The NCS family includes members with very different evolutionary rates, which provide insights into their diversification in both structure and function. Among these calcium sensors, Frq/NCS-1 appears to be one of the most conserved subfamilies, not only at the amino acid sequence level but also because of their very low propensity to retain gene duplications - no other fixed duplication has been detected here except in *D. rerio*. For that reason, the discovery of a fixed and long-term stable (estimated as > 100 Mya) duplication in *Drosophila*, as well as the rapid and heterogeneous accumulation of amino acid changes between duplicates, constitutes a very unusual feature for this family. Understanding the basis of this finding may contribute to the better knowledge of molecular evolution of NCS proteins as well as of important fields as the origin and fate of duplicated genes and of functional innovation.

Gene duplications arise initially in a single individual and can become fixed in the population by natural selection or by random genetic drift. Initially, duplications generate functional redundancy, a situation that is generally non-advantageous; hence, even if duplicates have been fixed by chance, the accumulation of mutations will result in the disruption of structure and function of one of the duplicates, which becomes a pseudogene (i.e. a non-functional gene). Results of nucleotide and amino acid variation analyses performed here clearly demonstrate that amino acid changes observed in *Drosophila *Frequenins are not the result of this pseudogenization process. The coding sequence of *frq*1 shows strong functional constraints across the *Drosophila *genus as well as within a *D. melanogaster *population (unpublished results) and none of the nine amino acid substitutions affecting the encoded protein was disruptive, pointing to purifying selection as the main force acting on this gene. In addition, analysis of loss-of-function and over-expression phenotypes also point to the role of both Frq1 and Frq2 on synaptic transmission and nerve terminal growth [9,11].

Several theoretical models have been proposed to explain the maintenance of duplicated genes, which consider different mechanisms of preservation and subsequent optimization [20-25]. The main differences among models rely in which part of the gene is involved (coding or non-coding sequences) and in the relative role of natural selection and genetic drift in determining the outcome of duplication. Dealing with protein sequence evolution, two duplicates can be preserved without the action of positive selection just by the selectively neutral division of different subsets of the original functions between daughter copies (product subfunctionalization). Nevertheless, this situation is not compatible with present data because of: i) the distribution of mutations after gene duplication is significantly different from the one expected under neutrality, and ii) the strongly selective constrains acting on amino acid positions affected by these mutations across the *Drosophila *genus. We would expect that positions with degenerative mutations removing ancestral functions evolved in a completely neutral way.

The amino acid substitution pattern observed in Frq1 might result from the fixation of nearly neutral mutations (and so governed largely by genetic drift) in an initial period of relaxation, just after gene duplication, followed by a subsequent increase of functional constraints in *Drosophila*. Environmental conditions responsible for these constraint changes would affect *frq*1 and *frq*2 in a different way as suggested by the highly biased distribution of amino acid substitutions detected between these paralogues.

Accordingly, some functional or regulatory diversification from the native state between *Drosophila *Frequenins would be needed. In this context, some models of structural evolution such as compensatory mutations [26] or conformational epistasis [27] might generate the observed evolutionary pattern. In the first, some variants are fixed by positive selection to compensate deleterious mutations in other epistatically interacting positions. Under this model, some of the amino acid substitutions in Frq1 ought to have been selected to maintain protein structure or function rather than be adaptive [28]. Under the conformational epistasis hypothesis, most of the Frq1 mutations should have been slightly deleterious or permissive substitutions (i.e. small-effect) that stabilized specific structural elements in this protein allowing further positively selected mutations-which in the absence of previous small-effect mutations should destabilize the protein. These mutations could have conferred a new function and then increased selective constraints in *Drosophila*. Although we have found evidences of molecular co-evolution across the Frq/NCS-1 subfamily, none of the predicted coevolving amino acids involves two positions with replacements in Frq1. This finding should rule out structural evolution as the main explanation for the rapid accumulation of amino acid substitutions between duplicates. Nevertheless, the probability of observing more double substitutions than expected by chance largely depends on the presence of relatively strong epistatic interactions between mutations. If both Frequenins have very few potentially permissive substitutions, the probability of observing repeated pathways across the subfamily should be very low. Under this situation, we will have very little power to detect signals of co-evolution between amino acid sites across the alignment. An in-depth experimental study would be needed to analyze the putative contribution of these structural evolution models in generating the pattern observed in *Drosophila *Frequenins.

The recurrent fixation of advantageous mutations might also account for the excess of amino acid substitutions in Frq1. ML estimate of the *d*_N_/*d*_S _ratio in the internal branch leading to *Drosophila *Frq1 sequences is actually higher than in the rest of the branches of the *Drosophila *Frq phylogeny. Although the estimate is considerably lower than 1, as well as lower than genomic averages reported for the *Drosophila *genus [29], this result does not exclude the possibility that positive selection acted in the fixation of certain Frq1 changes. It has been largely demonstrated that positive selection commonly acts on few amino acid positions in a protein and, therefore, present estimates based on the complete coding sequence could be too conservatives. Frequenin is a highly conserved protein, with very low ω estimates across bilateria (the pair-wise ω ratios calculated between vertebrate NCS-1 sequences range from 0 to 0.0065; *d*_N_/*d*_S _data from Ensembl Genome Browser) and, therefore, even a significant increase in the number of amino acid changes could not be greatly reflected in the average ω value calculated from the entire protein. In fact, when we applied to the data a much more powerful branch-site approach [30], results were marginally significant (results not shown). Even so, we have to interpret this result with caution because it has been reported that this method often generates false positives under certain conditions [31,32].

All simple models for the preservation of *frq1 *and *frq2 *in *Drosophila *considered above that are compatible with the existing data require the action of positive selection. Thus, the key question that remains unsolved is if natural selection promoted a functional change between Frq1 and Frq2 in *Drosophila*. We investigated whether positions differing between paralogues are involved in functional diversification between other members of the NCS family. The fact that many of these positions are candidates to participate in significant amino acid substitution rate shifts between NCS subfamilies suggests that Frq1 and Frq2 might have diverged (at least to some extent) in their functions. It is difficult to determine, however, the specific functional features that could have diverged between these two proteins. Most of the positions with changes between *Drosophila *Frequenins are among the most probable functionally diverged residues in many subfamily comparisons. The putative specialized roles in neural function of *Drosophila *Frequenins might result from differences in the affinity to Ca^2+^, sub-cellular location or targeted proteins [6]. It has been demonstrated that sites of the C-terminal part of the human NCS-1 interact with target proteins [33]. Also, we had shown that the last 33 amino acids of the *Drosophila *Frq1 and Frq2 act as dominant negative peptide, with effects in synaptic transmission and terminal morphology [9]. Thus, the two candidate C-terminal positions, 161 and 162 (and perhaps the position 187) might have promoted some diversification in the interaction with target proteins. On the other hand, the replacements observed in these two sites might have also produced changes in Ca^2+ ^binding either directly, because they are located in the fourth EF-hand, or by producing structural changes affecting protein thermostability and Ca^2+ ^affinity of the other EF-hands [34-36]. The position 79 is one of the coordinated residues of the second EF-hand and, therefore, replacements in this site might also be related with Ca^2+ ^affinity differences between paralogues. The amino acid fixed in Frq1 in this position is hydrophilic and highly exposed, in contrast to the hydrophobic and buried ancestor. This feature might indicate a possible structural change produced by the replacement in this position. Binding sites for target proteins have been also mapped in the N-terminal part of the human Frq/NCS-1protein [37]. Consequently, substitution in positions 58 (and perhaps in 79 and 102 in Frq1) might have altered the interaction properties of Frq2 with some of their partners. The other good candidate to participate in functional divergence between NCS subfamilies, the position 102, is located in the loop connecting the second and third EF-hands. The homologous region in GUCA2 determines the concentration of Ca^2+ ^that activates the target of this protein [38]. Then, the replacement in this position might also affect the Ca^2+ ^binding properties of Frq1, contributing to the functional divergence of duplicates in *Drosophila*.

Finally, in addition to the retention and diversification of protein coding regions, regulatory divergence is also prevalent in the evolution of duplicated genes [39]. In fact, it has been proposed that subfunctionalization of regulatory regions can increase the mutational space accessible to duplicates, removing selective constraints and allocating neofunctionalization [40]. The mRNAs of *frq1 *and *frq2 *are expressed in *D. melanogaster *with a similar spatio-temporal pattern, but with important quantitative differences [9]. These quantitative differences could be related with the significantly different silent evolutionary rate found between these two proteins since it has been shown that gene expression levels are negatively correlated with evolutionary rates [41]. Here we found that Brc and Eip74EF factors might be involved in the regulatory divergence of these two duplicates. Suitable Bcr binding sites are only present in regulatory regions of *frq1*. In fact, *frq1 *and *br *(the locus encoding Brc proteins) [42] mRNAs have a very similar expression pattern in late stages of embryo development (both appear in brain and ventral nervous system at embryo stages 13-16; http://www.fruitfly.org/cgi-bin/ex/insitu.pl), coincident with the peak of *frq1 *expression. *Eip74EF*, in contrast, appear only in early metamorphosis, after the major pulse of Ecdysone in the third larval instar, being consistent with the higher expression of *frq2 *mRNA in adult flies. Hence, differences in the response to these two Ecdysone-induced early genes might be responsible, at least in part, for the differences in gene expression levels between duplicates. This regulatory diversification might render the action of positive selection suitable, resulting in further functional diversification at the protein level. The current *in silico *analysis sets the frame of future experimental studies on the regulation of *frq1 *and *frq2 *expression, as well as on the functional mechanisms of the corresponding proteins.

## Conclusions

The increasing availability of sequenced genomes from polymorphic to highly divergent taxa has allowed the application of phylogenetic and molecular variation analyses across different time scales. This approach has proven useful to disentangle evolutionary processes that were not completely resolved in previous comparative genomic or population genetics studies. Using this approach we found that positive selection, acting on protein sequence evolution, jointly with the diversification of non-coding regulatory sequences, might be the main forces responsible for the origin and preservation of the unusual Frequenin duplication in *Drosophila*. The results of this study of variation at the molecular level of Frequenin sequences will inspire future experiments on the mechanisms for protein function and gene regulation.

## Methods

### Sequence searches

We used TBLASTN tool [43] against public databases to search homologues of the *Drosophila *Frq1 and Frq2 proteins. The databases surveyed were: i) the twelve complete *Drosophila *genomes available in FlyBase [44] ii) the genomes of invertebrate vectors of human pathogens in VectorBase [45], iii) the dbESTs database in GeneBank [46] and iv) eukaryotic genomes available in ENSEMBL project [47]. In addition, we retrieved all proteins annotated as members of the NCS family from ENSEMBL database (family accession number: ENSFM00500000269655). Orthologous relationships were assigned using the BLAST reciprocal best-hit approach together with gene tree and species tree reconciliation and synteny conservation. Gene structure features of *Drosophila *orthologues were revisited and adjusted using the information of FlyBase annotated genes (http://rana.lbl.gov/drosophila/; Drosophila 12 Genomes Consortium) as a guide.

### Nucleotide sequence analyses

The DNA sequences corresponding to the coding regions of *frq*1 and *frq*2 genes were multiple aligned using the Mafft software [48], and manually edited with MacClade version 3 program [49]. The resulting multiple sequence alignment (MSA) was used to estimate nucleotide sequence variation in DnaSP version 5.0 [50], and MEGA version 4 [51] software. To determine if paralogous genes evolve at different substitution rates we conducted the two-cluster test implemented in the LINTRE package [52].

BEAST version 1.4.8 [53] was used to place the duplication event that produces *frq1 *and *frq2 *copies on the insect phylogeny and to obtain a posterior density of the duplication age. The tree was built using the 21 complete *Drosophila *coding regions available plus the sequence of 6 other insects. We set constraints on divergence times of i) the *Drosophila *and *Sophophora *subgenera, ii) the *melanogaster *and *obscura *groups and iii) the divergence of the species belonging to the *melanogaster *subgroup. These ages were set in uniform distributions with ranges previously published [54]. We used divergence at synonymous sites with a relaxed, uncorrelated lognormal clock and the HKY85 nucleotide substitution model [55] with gamma rate heterogeneity. The length of the Markov chain was 5000000, with a sample frequency every 200. The first 2500 trees were discarded in the burn-in step.

Selective pressures acting on coding regions after gene duplication and the estimates of synonymous and nonsynonymous rates, including total tree length for synonymous substitutions, were estimated using the ML approach implemented in PAML version 4 [56]. Since homologous sequences identified in mosquito and *Drosophila *are highly divergent (they might yield unreliable estimates of the relevant parameters) we used only the 21 *Drosophila *coding regions and the sequence of the fly *Ceratitis capitata *for the analysis. The ratio of nonsynonymous (*d*_N_) to synonymous substitution rates (*d*_s_), ω = *d*_N_/*d*_S_, was used as the measure of protein selective constraints [57]. Competing models (Figure 4) were compared in a Likelihood Ratio Test (LRT), assuming that twice the log likelihood difference between the two models (2Δℓ) follows a χ^2 ^distribution with a number of degrees of freedom equal to the difference in the number of free parameters [58]. To prevent incorrect parameter estimates caused by local optima, the program was run multiple times for the same model, specifying different initial values.

In order to identify the regions of DNA sequence similarity produced by the duplication event, a Dot Plot of the complete genomic region (~66 kb) that includes *frq1 *and *frq2 *(and *andorra*) genes in *D. melanogaster *was obtained in the Zpicture tool web [59]. Moreover, a MSA corresponding to this genomic region in 6 *Drosophila *genomes (*D. melanogaster*, *D. simulans*, *D. erecta*, *D. yakuba*, *D. ananassae*, and *D. pseudoobscura*) was obtained from the pre-computed alignments of Comparative Assembly Freeze 1 (CAF1) http://rana.lbl.gov/drosophila/caf1.html available in VISTA server [60]. This MSA was used to perform a phylogenetic shadowing [61] search for conserved (orthologous) non-coding regions using the hidden Markov model (phylo-HMM) approach implemented in PhastCons [62]. We used the UCSC genome browser database [63] aligment of all CDSs in the genomic fragment corresponding to the positions chrX:2000000-3000000 for modelling sequence conservation. The same MSA was also analysed with the Chai algorithm to obtain a profile of protein binding affinity based on local DNA topology [64]. Finally, regions selected as candidates to include functional regulatory elements were scanned for the presence of insect transcription factor binding site profiles contained in TRANSFAC [65] and JASPAR [66] collections.

### Amino acid sequence analyses

For protein sequence analyses, we built two different MSA. The first (Additional file 4) included all bilaterian members of the Frequenin subfamily retrieved from databases and was used to analyze amino acid co-evolution by means of two approaches. In one of them, the correlated variance of the evolutionary rates between amino acid sites was calculated in CAPS software [67]. In the other, a ML approach [18] based in a continuous time Markov substitution process (CTMP) was applied to the data. This method compares the goodness of fit of a null model, in which each pair of sites evolves independently, and an alternative coevolving model obtained by reweighing the independent substitution rate matrix to favour double over single changes. For this analysis, we used a ε value of 0.75 and only the positions with a *log-odds score *threshold > 15 were considered. The second protein MSA, with only vertebrate sequences but with representatives of the entire NCS family, was used to identify the amino acid positions with significant shifts in substitution rates, likely responsible for changes in functional constrains and functional diversification between NCS subfamilies. We analyzed only vertebrate sequences to avoid large unbalanced trees (i.e., only vertebrates have representatives of all NCS subfamilies), which can produce biased results [19]. We discarded sequences representing species or lineage specific expansions, which likely reflect particular histories, partial sequences and sequences that produce poor aligned fragments (Additional file 5). Type I and type II functional divergence coefficients [68,19] as well as the posterior probability (PP) profiles to contribute to functional divergence of all amino acid sites were calculated in Diverge 2.0 software [69]. ML trees for these analyses were built in PhyML [70] using the best-fit amino acid substitution model selected in MODELTEST 3.7 [71]. Sites with the 5% highest *PP *values were considered as firm candidates to be functionally diverged sites. Functional divergence between KChIP and GCAP subfamilies was not analyzed because of the high level of divergence and the poor quality of the alignment.

Neurocalcin-δ and VILIP1 subfamilies were not used for this analysis because of their lack of amino acid rate heterogeneity. The probability of observing a given distribution of amino acid changes between paralogues after gene duplication was calculated with the Relative Rate Test module implemented in Hyphy package [72]. This test was performed using the previously selected amino acid substitution model.

The putative 3D structure of the ancestral Frequenin (inferred by ML ancestral reconstruction in PAML 4) was obtained with Swiss-Model server [73]. The 3D structure of human Frequenin (PDB ID: 1G8I) [74,75] was used as template for this analysis. The model was visualized in Swiss-PdbViewer program version 4 [76]. This program was also used to highlight the relevant amino acid replacements identified in the evolutionary analyses.

## Authors' contributions

AS-G designed the study, carried out the analyses and wrote the first version of the manuscript. JR-P provided complementary information and modified the text. AF supervised the study and edited the final text. All authors read and approved the final manuscript.

## Supplementary Material

Additional file 1 **Dot plot of the *frq *genomic region**. Dot Plot of the genomic region enclosing *frq1 *and *frq2 *genes in *Drosophila melanogaster *against itself. Click here for file

Additional file 2 **Conservation, DNA topology and transcription factor binding sites predictions**. Conservation, DNA topology and transcription factor (TF) binding sites profiles of the different *frq1 *and *frq2 *non-coding regions analyzed. Red and blue lines indicate sequence conservation across species and DNA accessibility, respectively. The Y-axis indicates either the posterior probability of each individual site to be conserved relative to the genomic fragment used as reference in PhastCons [62] or the normalized score (the score of each site divided by the maximum of the investigated region) obtained in Chai [64]. Vertical coloured bars indicate the conserved elements predicted by the Viterbi algorithm in PhastCons (the probability of conservation increases from blue to red). Horizontal bars indicate the positions predicted to contain insect TF binding site profiles present in TRANSFAC [65] and JASPAR [66] collections. Click here for file

Additional file 3 **Amino acid evolutionary rates in NCS-1 subfamily**. Distribution of amino acid evolutionary rates across NCS-1 protein. Asterisks show the amino acid positions that differ between Frq1 and Frq2. The evolutionary rate in these positions is coloured in grey. Click here for file

Additional file 4 **Frq/NCS-1 subfamily multiple sequence alignment**. Multiple sequence alignment with all bilaterian NCS-1 sequences used in the amino acid sequence evolution analysis of this subfamily. Data is in NBRF format. Click here for file

Additional file 5 **Data set of vertebrate NCS family sequences**. Data set of vertebrate NCS sequences used in the functional divergence analysis. Species names are in the same four-letter format as in pre-computed alignments downloaded from ENSEMBL (except for Frequenin subfamily sequences, which are named as in Additional file 4. Data is in NBRF format. Click here for file
