# Supplementary figures and images for: Two Frequenins in Drosophila: unveiling the evolutionary history of an unusual Neuronal Calcium Sensor (NCS) duplication
Source: BMC Evol Biol. 2010 Feb 19;10:54. doi: 10.1186/1471-2148-10-54 (PMC2837045; doi:10.1186/1471-2148-10-54)

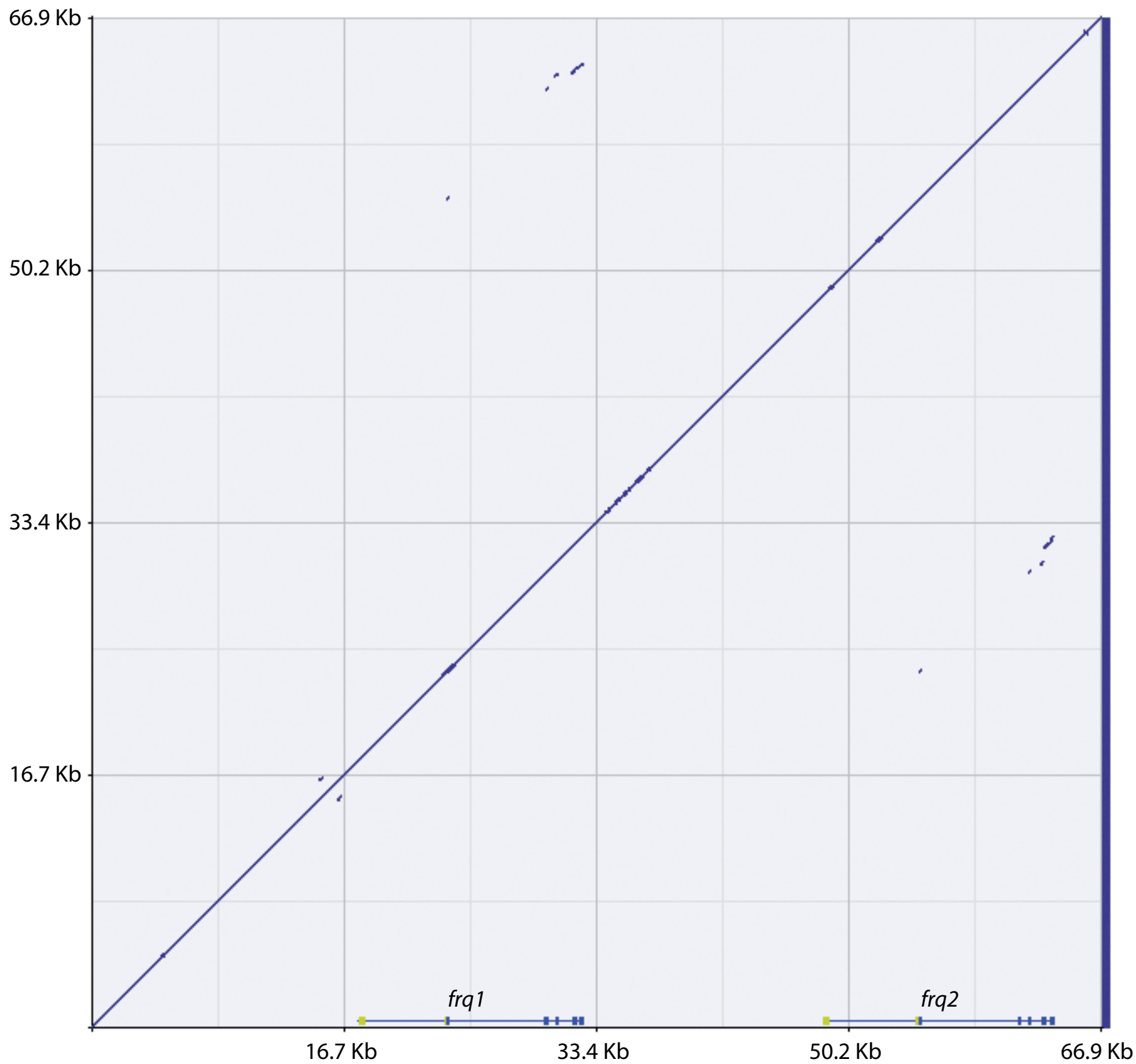

Supplement: Additional file 1 — Dot plot of the frq genomic region. Dot Plot of the genomic region enclosing frq1 and frq2 genes in Drosophila melanogaster against itself. [file 1471-2148-10-54-S1.PDF]

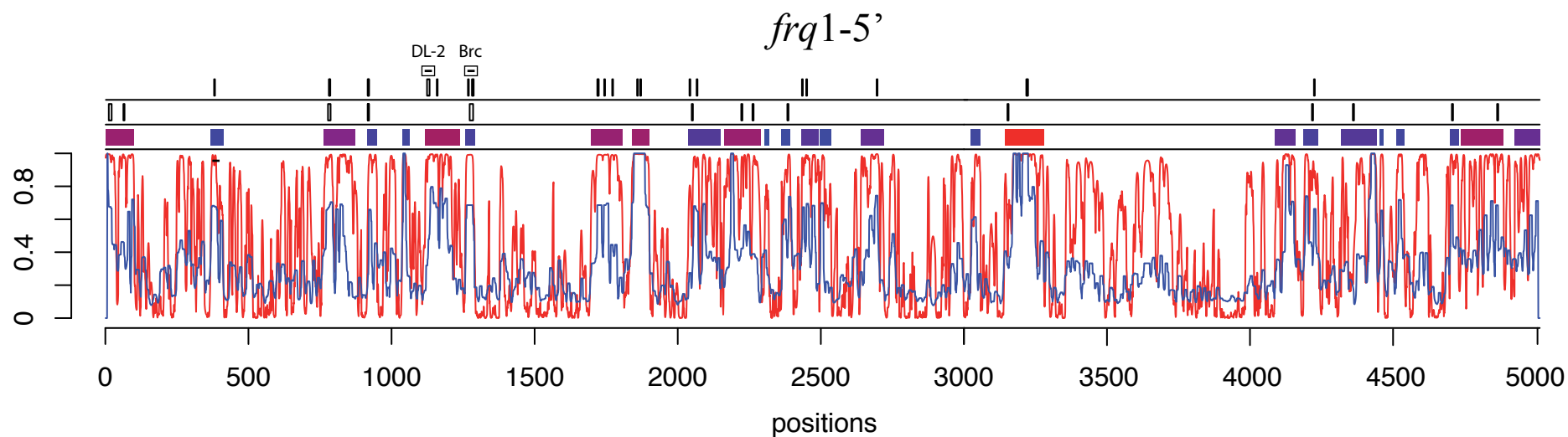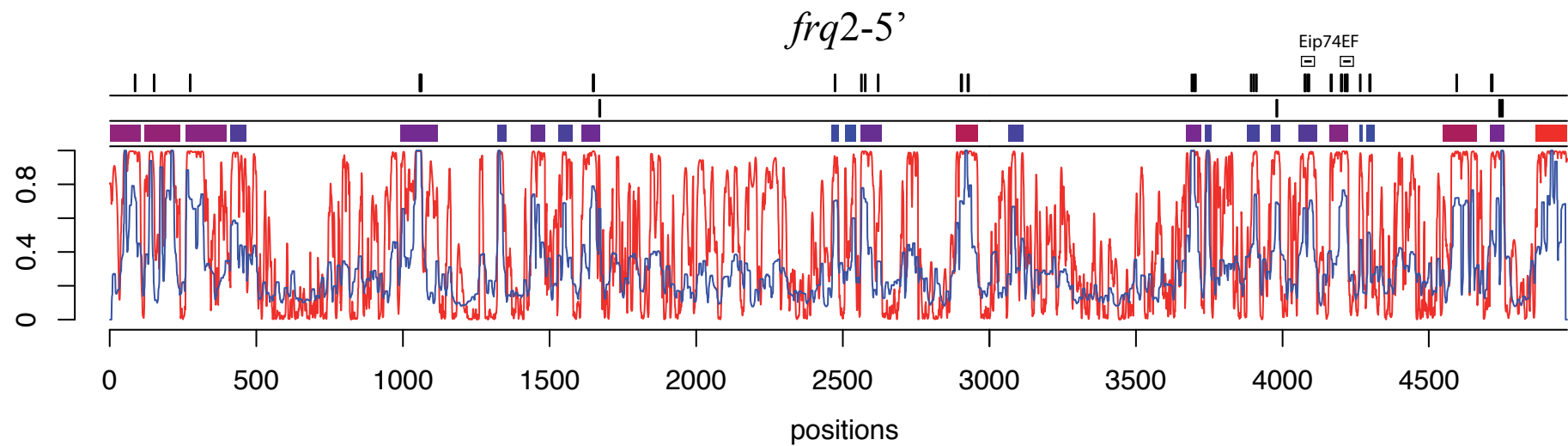

*frq1*-intron1

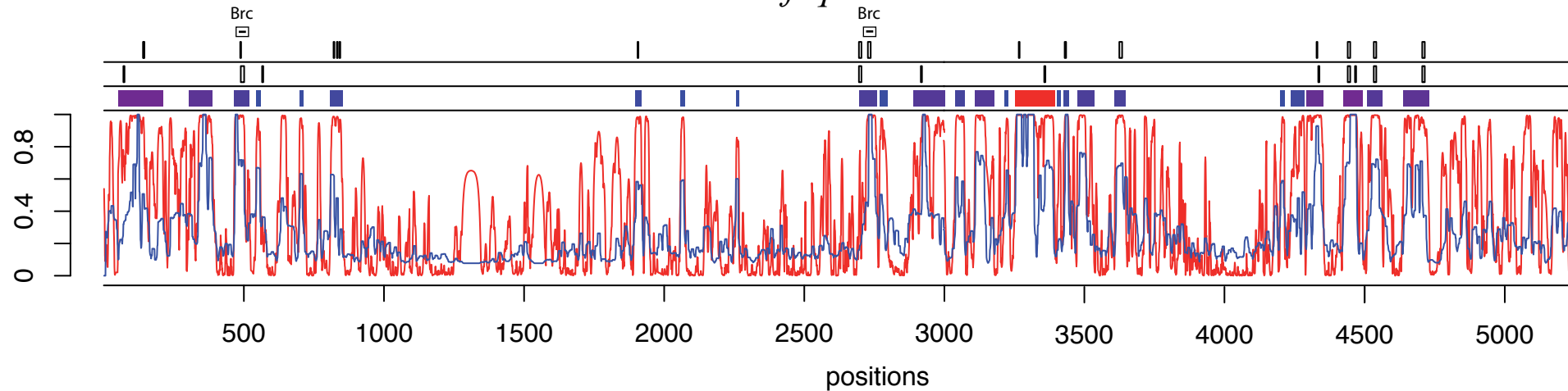

*frq2*-intron1

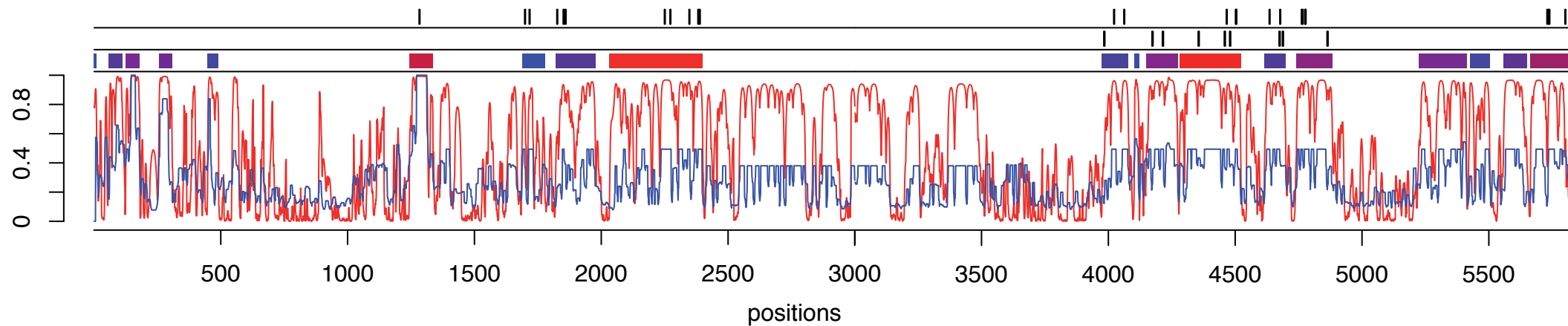

*frq1*-intron2

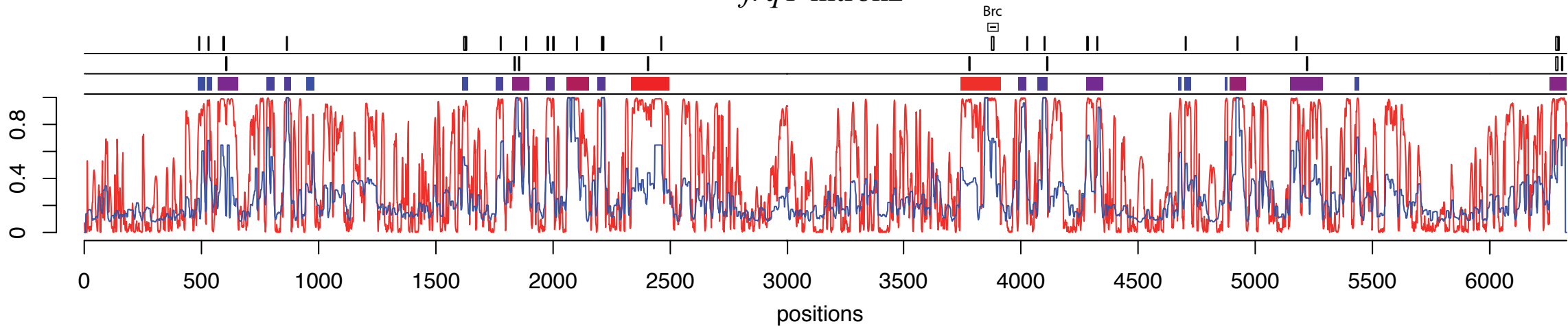

*frq2*-intron2

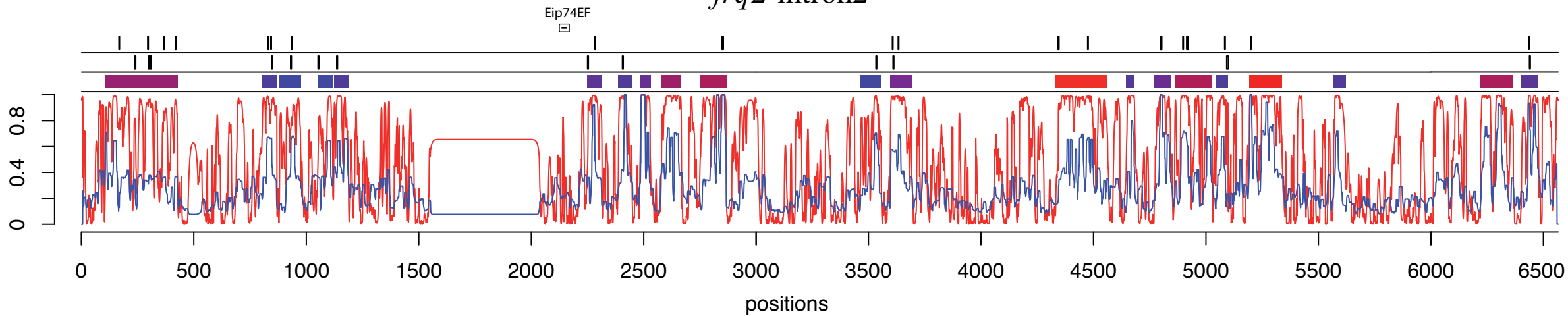

Supplement: Additional file 2 — Conservation, DNA topology and transcription factor binding sites predictions. Conservation, DNA topology and transcription factor (TF) binding sites profiles of the different frq1 and frq2 non-coding regions analyzed. Red and blue lines indicate sequence conservation across species and DNA accessibility, respectively. The Y-axis indicates either the posterior probability of each individual site to be conserved relative to the genomic fragment used as reference in PhastCons [62] or the normalized score (the score of each site divided by the maximum of the investigated region) obtained in Chai [64]. Vertical coloured bars indicate the conserved elements predicted by the Viterbi algorithm in PhastCons (the probability of conservation increases from blue to red). Horizontal bars indicate the positions predicted to contain insect TF binding site profiles present in TRANSFAC [65] and JASPAR [66] collections. [file 1471-2148-10-54-S2.PDF]

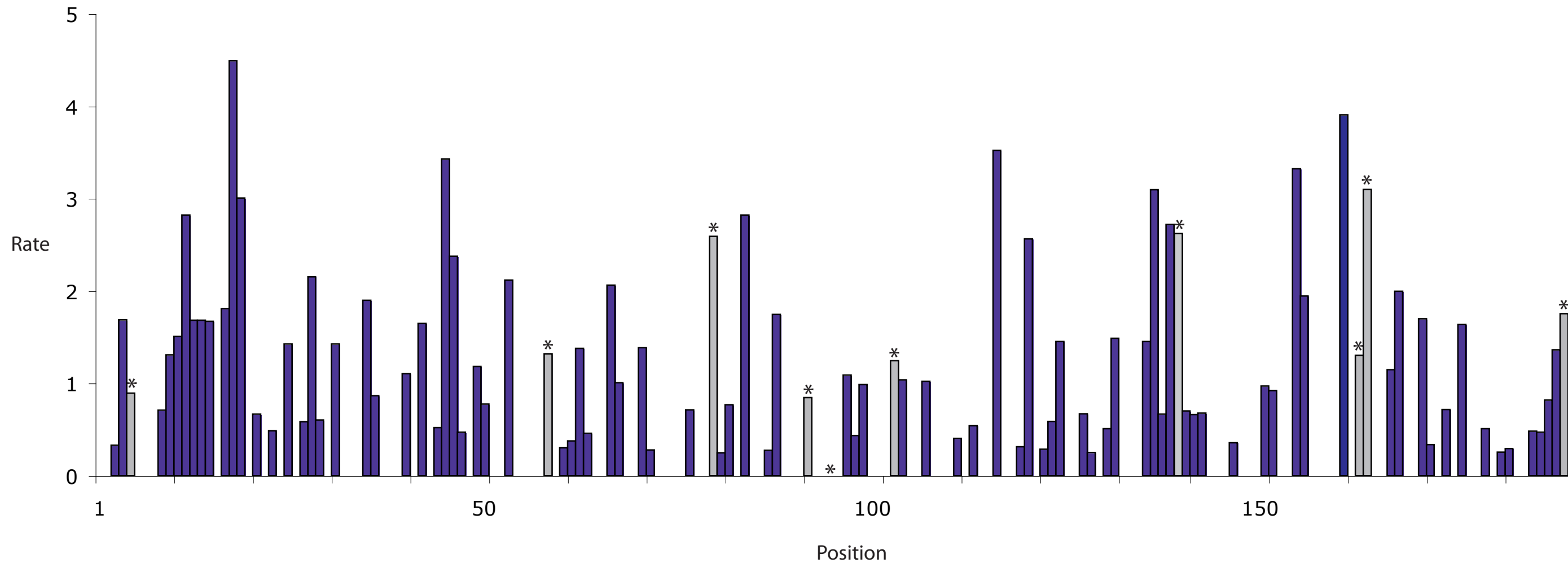

Supplement: Additional file 3 — Amino acid evolutionary rates in NCS-1 subfamily. Distribution of amino acid evolutionary rates across NCS-1 protein. Asterisks show the amino acid positions that differ between Frq1 and Frq2. The evolutionary rate in these positions is coloured in grey. [file 1471-2148-10-54-S3.PDF]
