# Supplementary material for: Two Frequenins in Drosophila: unveiling the evolutionary history of an unusual Neuronal Calcium Sensor (NCS) duplication
Source: BMC Evol Biol. 2010 Feb 19;10:54. doi: 10.1186/1471-2148-10-54 (PMC2837045; doi:10.1186/1471-2148-10-54)
Supplement: Additional file 4 — Frq/NCS-1 subfamily multiple sequence alignment. Multiple sequence alignment with all bilaterian NCS-1 sequences used in the amino acid sequence evolution analysis of this subfamily. Data is in NBRF format. [file 1471-2148-10-54-S4.DOC]

>P1; Drosophila_melanogaster_Frq1

Drosophila_melanogaster_Frq1

MGKKSSKLKQDTIDRLTTDTYFTEKEIRQWHKGFLKDCPNGLLTEQGFIKIYKQFFPQGDPSKFASLVFRVFDEN

NDGSIEFEEFIRALSVTSKGNLDEKLQWAFRLYDVDNDGYITREEMYNIVDAIYQMVGQQPQ-SEDENTPQKRVD

KIFDQMDKNHDGKLTLEEFREGSKADPRIVQALSLGGG----*

>P1; Drosophila_melanogaster_Frq2

Drosophila_melanogaster_Frq2

MGKKNSKLKQDTIDRLTTDTYFTEKEIRQWHKGFLKDCPNGLLTEQGFIKIYKQFFPDGDPSKFASLVFRVFDEN

NDGAIEFEEFIRALSITSRGNLDEKLHWAFRLYDVDNDGYITREEMYNIVDAIYQMVGQQPQ-TEDENTPQKRVD

KIFDQMDKNHDDRLTLEEFREGSKADPRIVQALSLGGD----*

>P1; Ceratitis_capitata_Frq

Ceratitis_capitata_Frq

MGKKNSKLKQDTIDRLTTATYFTEKEIRQWHKGFLKDCPNGLLTEQGFIKIYKQFFPQGDPSKFASLVFRVFDEN

EDGAIEFEEFIRALSITSRGNLDEKLQWAFRLYDVDNDGYITREEMYNIVDAIYQMVGQQPQ-TEEENTPQKRVD

KIFDQMDKNHDDRLTLDEFREGSKADPRIVQALSLGGD----*

>P1; Anopheles_gambiae_Frq

Anopheles_gambiae_Frq

MGKKNSKLKQDAIDRLTTATYFTEKEIRQWHKGFLKDCPNGLLTEQGFIKIYKQFFPQGDPSKFASLVFRVFDEN

NDGSIEFEEFIRALSITSRGNLDEKLHWAFRLYDVDNDGFITRDEMYNIVDAIYQMVGQQPQ-SEDDNTPQKRVD

KIFDQMDKNHDDRLTLEEFREGSKADPRIVQALSLGGD----*

>P1; Aedes_aegypti_Frq

Aedes_aegypti_Frq

MGKKNSKLKQDTIDRLTTATYFTEKEIRQWHKGFLKDCPNGLLTEQGFIKIYKQFFPQGDPSKFASLVFRVFDEN

NDGSIEFEEFIRALSITSRGNLDEKLHWAFRLYDVDNDGFITRDEMYNIVDAIYQMVGQQPQ-TEDENTPQKRVD

KIFDQMDKNHDDRLTLEEFREGSKADPRIVQALSLGGD----*

>P1; Culex_pipiens_Frq

Culex_pipiens_Frq

MGKKNSKLKQDTIDRLTTATYFTEKEIRQWHKGFLKDCPNGLLTEQGFIKIYKQFFPQGDPSKFASLVFRVFDEN

NDGSIEFEEFIRALSITSRGNLDEKLHWAFRLYDVDNDGFITRDEMYNIVDAIYQMVGQQPQ-TEDENTPQKRVD

KIFDQMDKNHDDRLTLEEFREGSKADPRIVQALSLGGD----*

>P1; Bombyx_mori_Frq

Bombyx_mori_Frq

MGKKNSKLKQDTIDRLTSATYFTEKEIRLWHKGFLKDCPNGLLTEQGFIKIYKQFFPQGDPSKFASLVFRVFDEN

NDGSIEFEEFIRALSVTSRGNLDEKLHNAFRLYDVDNDGYITRDEMYNIVDAIYQMVGQTPQ-PEDENTPQKRVD

KIFDQMDKNHDDRLTLEEFREGSKADPRIVQALSLGGD----*

>P1; Tribolium_castaneum_Frq

Tribolium_castaneum_Frq

MGKKNSKLKQDTIDRLTTATYFTEKEIRQWHKGFLKDCPNGLLTEQGFIKIYKQFFPQGDPSKFASLVFRVFDEN

NDGSIEFEEFIRALSVTSRGNLDEKLHWAFRLYDVDNDGFITRDEMYNIVDAIYQMVGQQP--SEDENTPQKRVD

KIFDQMDKNHDDRLTLEEFREGSKADPRIVQALTLGPE----*

>P1; Acyrthosiphon_pisum_Frq

Acyrthosiphon_pisum_Frq

MGKRNSKLKQDTIDKLIEDTYFSEKEIRQWHKGFLKDCPNGLLTEQGFIKIYRQFFPQGDPTKFASLVFRVFDEN

KDGSIEFEEFIKALSVTSRGNLEEKLHWAFRLYDVDNDGFITRDEMYNIVDAIYQMVGQQPQ-AEDENTPQKRVD

KIFDQMDKNHDDKLTLEEFREGSKADPRIVQALSLGGD----*

>P1; Nasonia_vitripennis_Frq

Nasonia_vitripennis_Frq

MGKKNSKLKQDTIDRLTTDTYFTEKEIRSWHKGFLKDCPDGLLTEQGFIKIYKQFFPHGDPSKFASLVFRVFDEN

NDGTIEFEEFIRALSVTSRGNLDEKLHWAFRLYDVDNDGFITRDEMYNIVDAIYQMVGQAPQ-AEDENTPQKRVD

KIFDQMDKNHDDKLTLEEFREGSKADPRIVQALSLGGPPIDG*

>P1; Myzus_persicae_Frq

Myzus_persicae_Frq

MGKRNSKLKQDTIDKLIEDTYFSEKEIRQWHKGFLKDCPNGLLTEQGFIKIYRQFFPQGDPTKFASLVFRVFDEN

KDGSIEFEEFIKALSVTSRGNLDEKLHWAFRLYDVDNDGFITRDEMYNIVDAIYQMVGQQPQ-AEDENTPQKRVD

KIFDQMDKNHDDKLTLEEFREGSKADPRIVQALSLGGD----*

>P1; Lernaeocera_branchialis_Frq

Lernaeocera_branchialis_Frq

MGKNNSKLNNETLNKLTSETYFTEKEIKQWYKGFLKDCPNGLLTEQGFIKIYTQFFPNGDPTKFASLVFRVFDEN

KDGSIEFEEFIRALSVTSRGNLDEKLHWAFRLYDVDNDGYITRKEMYDIVDAIYQMVGQQSA-EDDDNTPQKRVE

KIFNQMDKNGDDKLTLDEFREGSKADPRIVQALSL-------*

>P1; Molgula_tectiformis_Frq

Molgula_tectiformis_Frq

MGNRKSKLKPEIMEKLTKETKFTEAELNQWYKGFLHDCPTGKLSYEEFKEIYKQFFPQGNSEKFAKFVFHTFDEN

KDGTVEFEEFILALSITSRGTLEEKLNWAFQLYDLDNDGYITRKEMLDIVSAIFAMVGDAVQLPEEENTPQKRVD

KIFGSMDKNQDGKLTKEEFLEGAKNDPSIVQALSIYDGL--V*

>P1; Bos_taurus_Frq

Bos_taurus_Frq

MGKSNSKLKPEVVEELTRKTYFTEKEVQQWYKGFIKDCPSGQLDAAGFQKIYKQFFPFGDPTKFATFVFNVFDEN

KDGRIEFSEFIQALSVTSRGTLDEKLRWAFKLYDLDNDGYITRNEMLDIVDAIYQMVGNTVELPEEENTPEKRVD

RIFAMMDKNADGKLTLQEFQEGTKADPSIVQALSLYDGL--V*

>P1; Gallus_gallus_Frq

Gallus_gallus_Frq

MGKSNSKLKPEVVEELTRKTYFTEKEVQQWYKGFIKDCPSGQLDAAGFQKIYKQFFPFGDPTKFATFVFNVFDEN

KDGRIEFSEFIQALSVTSRGTLDEKLRWAFKLYDLDNDGYITRNEMLDIVDAIYQMVGNTVELPEEENTPEKRVD

RIFAMMDKNADGKLTLQEFQEGSKADPSIVQALSLYDGL--V*

>P1; Homo_sapiens_Frq

Homo_sapiens_Frq

MGKSNSKLKPEVVEELTRKTYFTEKEVQQWYKGFIKDCPSGQLDAAGFQKIYKQFFPFGDPTKFATFVFNVFDEN

KDGRIEFSEFIQALSVTSRGTLDEKLRWAFKLYDLDNDGYITRNEMLDIVDAIYQMVGNTVELPEEENTPEKRVD

RIFAMMDKNADGKLTLQEFQEGSKADPSIVQALSLYDGL--V*

>P1; Pongo_pygmaeus_Frq

Pongo_pygmaeus_Frq

MGKSNSKLKPEVVEELTRKTYFTEKEVQQWYKGFIKDCPSGQLDAAGFQKIYKQFFPFGDPTKFATFVFNVFDEN

KDGRIGFSEFIQALSVTSRGTLDEKLRWAFKLYDLDNDGYITRNEMLDIVDAIYQMVGNTVELPEEENTPEKRVD

RIFAMMDKNADGKLTLQEFQEGSKADPSIVQALSLYDGL--V*

>P1; Ratus_norvegicus_Frq

Ratus_norvegicus_Frq

MGKSNSKLKPEVVEELTRKTYFTEKEVQQWYKGFIKDCPSGQLDAAGFQKIYKQFFPFGDPTKFATFVFNVFDEN

KDGRIEFSEFIQALSVTSRGTLDEKLRWAFKLYDLDNDGYITRNEMLDIVDAIYQMVGNTVELPEEENTPEKRVD

RIFAMMDKNADGKLTLQEFQEGSKADPSIVQALSLYDGL--V*

>P1; Mus_musculus_Frq

Mus_musculus_Frq

MGKSNSKLKPEVVEELTRKTYFTEKEVQQWYKGFIKDCPSGQLDAAGFQKIYKQFFPFGDPTKFATFVFNVFDEN

KDGRIEFSEFIQALSVTSRGTLDEKLRWAFKLYDLDNDGYITRNEMLDIVDAIYQMVGNTVELPEEENTPEKRVD

RIFAMMDKNADGKLTLQEFQEGSKADPSIVQALSLYDGL--V*

>P1; Monodelphis_domestica_Frq

Monodelphis_domestica_Frq

MGKSNSKLKPEVVEELTRKTYFTEKEVQQWYKGFIKDCPSGQLDAAGFQKIYKQFFPFGDPTKFATFVFNVFDEN

KDGRIEFSEFIQALSVTSRGTLDEKLRWAFKLYDLDNDGYITRNEMLDIVDAIYQMVGNTVELPEEENTPEKRVD

RIFAMMDKNSDGKLTLQEFQEGSKADPSIVQALSLYDGL--V*

>P1; Xenopus_tropicalis_Frq

Xenopus_tropicalis_Frq

MGKSNSKLKPEVVEELTRKTYFTEKEVQQWYKGFIKDCPSGQLDAAGFQKIYKQFFPFGDPTKFATFVFNVFDEN

KDGRIEFSEFIQALSVTSRGTLDEKLRWAFKLYDLDNDGYITRNEMLDIVDAIYQMVGNTVELPEEENTPEKRVD

RIFAMMDKNSDGKLTLQEFQEGSKADPSIVQALSLYDGL--V*

>P1; Danio_rerio_Frq1

Danio_rerio_Frq1

MGKSNSKLKPEVVEELTRKTYFTEKEVQQWYKGFIKDCPSGQLDAAGFQKIYKQFFPFGDPTKFASFVFNVFDEN

KDGRIEFSEFIQALSVTSRGTLDEKLRWAFKLYDLDNDGYITRDEMLNIVDAIYQMVGNTVDLPEEENTPEKRVD

RIFAMMDKNADGKLTLQEFQEGSKADPSIVQALSLYDGL--V*

>P1; Danio_rerio_Frq2

Danio_rerio_Frq2

MGKSNSKLKPEVVEDLCRKTYFTEKEVQQWYKGFIKDCPSGQLDSSGFQKIYKQFFPFGDPTKFATFVFNVFDEN

KDGRIEFSEFIQALSVTSRGTLDEKLRWAFKLYDLDNDGYITRDEMLNIVDAIYQMVGNTVELPEEENTPEKRVD

RIFAMMDKNADGMLTLQEFQEGSKADPSIVQALSLYDGL--V*

>P1; Oryzias_latipes_Frq

Oryzias_latipes_Frq

MGKSNSKLKPEVVEELTRKTYFTEKEVQQWYKGFIKDCPSGQLDAVGFQKIYKQFFPFGDPTKFASFVFNVFDEN

KDGRIEFSEFIQALSVTSRGTLDEKLRWAFKLYDLDNDGYITRDEMLNIVDAIYQMVGNTVELPEEENTPEKRVD

RIFAMMDKNADGKLTLQEFQEGSKADPSIVQALSLYDGL--V*

>P1; Gasterosteus_aculeatus_Frq

Gasterosteus_aculeatus_Frq

MGKSNSKLKPEVVEELTRKTYFTEKEVQQWYKGFIKDCPSGQLDAVGFQKIYKQFFPFGDPTKFASFVFNVFDEN

KDGRIEFSEFIQALSVTSRGTLDEKLRWAFKLYDLDNDGYITRDEMLNIVDAIYQMVGNSVELPEEENTPEKRVD

RIFAMMDKNADGKLTLQEFQEGSKADPSIVQALSLYDGL--V*

>P1; Takifugu_rubripes_Frq

Takifugu_rubripes_Frq

MGKSNSKLKPEVVEELTRKTYFTEKEVQQWYKGFIKDCPSGQLDAAGFQKIYKQFFPFGDPTKFASFVFNVFDEN

KDGRIEFSEFIQALSVTSRGTLDEKLRWAFKLYDLDNDGYITRDEMLNIVDAIYQMVGNTVELPEEENTPEKRVD

RIFAMMDKNADGKLTLQEFQEGSKADPSIVQALSLYDGL--V*

>P1; Tetraodon_nigroviridis_Frq

Tetraodon_nigroviridis_Frq

MGKSNSKLKPEVVEELTSKTYFTEKEVQQWYKGFIKDCPSGQLDAAGFQKIYKQFFPFGDPTKFASFVFNVFDEN

KDGRIEFSEFIQALSVTSRGTLDEKLRWAFKLYDLDNDGYITRDEMLNIVDAIYQMVGNTVELPEEENTPEKRVD

RIFAMMDKNADGKLTLQEFQEGSKADPSIVQALSLYDGL--V*

>P1; Procavia_capensis_Frq

Procavia_capensis_Frq

MGKSNSKLKPEVVEELTRKTYFTEKEVQQWYKGFIKDCPSGQLDAAGFQKIYKQFFPFGDPTKFATFVFNVFDEN

KDGRIEFSEFIQALSVTSRGTLDEKLRWAFKLYDLDNDGYITRNEMLDIVDAIYQMVGNTVELPEEENTPEKRVD

RIFAMMDKNADGKLTLQEFQEGSKADPSIVQALSLYDGL--V*

>P1; Taeniopygia_guttata_Frq

Taeniopygia_guttata_Frq

MGKSNSKLKPEVVEELTRKTYFTEKEVQQWYKGFIKDCPSGQLDAAGFQKIYKQFFPFGDPTKFATFVFNVFDEN

KDGRIEFSEFIQALSVTSRGTLDEKLRWAFKLYDLDNDGYITRNEMLDIVDAIYQMVGNTVELPEEENTPEKRVD

RIFAMMDKNADGKLTLQEFQEGSKADPSIVQALSLYDGL--V*

>P1; Canis_familiaris_Frq

Canis_familiaris_Frq

MGKSNSKLKPEVVEELTRKTYFTEKEVQQWYKGFIKDCPSGQLDAAGFQKIYKQFFPFGDPTKFATFVFNVFDEN

KDGRIEFSEFIQALSVTSRGTLDEKLRWAFKLYDLDNDGYITRNEMLDIVDAIYQMVGNTVELPEEENTPEKRVD

RIFAMMDKNADGKLTLQEFQEGSKADPSIVQALSLYDGL--V*

>P1; Perca_flavescens_Frq

Perca_flavescens_Frq

MGKSNSKLKPEVVEELTRKTYFTEKEVQQWYKGFIKDCPSGQLDAVGFQKIYKQFFPFGDPTKFASFVFNVFDEN

KDGRIEFSEFIQALSVTSRGTLDEKLRWAFKLYDLDNDGYITRDEMLNIVDAIYQMVGNTVELPEEENTPEKRVD

RIFAMMDKNADGKLTLQEFQEGSKADPSIVQALSLYDGL--V*

>P1; Pimephales_promelas_Frq

Pimephales_promelas_Frq

MGKSSSKLKPEVVEELCRKTYFTEKEVQQWYKGFIKDCPSGQLDAAGFQKIYKQFFPFGDPTKFATFVFNVFDEN

KDGRIEFSEFIQALSVTSRGTLDEKLRWAFKLYDLDNDGYITRDEMLNIVDAIYQMVGNTVELPEEENTPEKRVD

RIFAMMDKNADGLLTLQEFQEGSKGDPSIVQALSLYDGL--V*

>P1; Salmo_salar_Frq

Salmo_salar_Frq

MGKSNSKLKPEMVEELTRKTYFTEKEVQQWYKGFIKDCPSGQLDSAGFQKIYKQFFPFGDPTKFASFVFNVFDEN

KDGRIEFAEFIQALSVTSRGTLDEKLRWAFKLYDLDNDGYITRDEMLNIVDAIYQMVGNTVELPEEENTPEKRVD

RIFAMMDKNADGLLTLKEFQEGSKADPSIVQALSLYDGL--V*

>P1; Xenopus_laevis_Frq

Xenopus_laevis_Frq

MGKSNSKLKPEVVEELTRKTYFTEKEVQQWYKGFIKDCPSGQLDATGFQKIYKQFFPFGDPTKFATFVFNVFDEN

KDGRIEFSEFIQALSVTSRGTLDEKLRWAFKLYDLDNDGYITRNEMLDIVDAIYQMVGNTVELPEEENTPEKRVD

RIFAMMDKNSDGKLTLQEFQEGSKADPSIVQALSLYD-----*

>P1; Idiosepius_paradoxus_Frq

Idiosepius_paradoxus_Frq

MGKRNSKLKPDEIEELRTKTYFSEEEIQQWYKGFMKDCPDGKLTLEGFTKIYRQFFPFGDPSKFAAFVFNVFDEN

KDGYIEFDEFLQALSVTSRGNVDEKLRWAFRLYDLDSDGFITREELLDIVDAIYKMVGNMVKLPEEENTPEKRVN

KIFEIMDKNKDDRLTFDEFLEGSKKDPTIIQALTLYDG----*

>P1; lymnaea_stagnalis_Frq

lymnaea_stagnalis_Frq

MGKRASKLRPEEVDELKAHTYFTESEIKQWHKGFRKDCPDGKLTLEGFTKIYQQFFPFGDPSKFANFVFNVFDEN

KDGFISFSEFLQALSVTSRGTVEEKLKWAFRLYDLDNDGYITRDELLDIVDAIYRMVGESVTLPEEENTPEKRVN

RIFQVMDKNKDDQLTFEEFLEGSKEDPTIIQAL---------*

>P1; Ciona_intestinalis_Frq

Ciona_intestinalis_Frq

MGNRKSKLKPEVLEKLTKQTKFTEAELHQWHKGFLHDCPTGKLSYEEFQGIYRQFFPQGDSAKFAKLVFTTFDEN

KDGTVEFDEFIIALSVTSRGSLDEKLHWAFQLYDLDNDGFITKNEMLNIVEAIFAMVGDAVNLPAEENTPQKRVE

KIFNVMDKNKDGKLTKEEFLVGAKSDPSIVQALSIYDGL--V*

>P1; Caenorhabditis_elegans_Frq

Caenorhabditis_elegans_Frq

MGKGNSKLKSSQIRDLAEQTYFTEKEIKQWYKGFVRDCPNGMLTEAGFQKIYKQFFPQGDPSDFASFVFKVFDEN

KDGAIEFHEFIRALSITSRGNLDEKLHWAFKLYDLDQDGFITRNEMLSIVDSIYKMVGSSVQLPEEENTPEKRVD

RIFRMMDKNNDAQLTLEEFKEGAKADPSIVHALSLYEGLSS-*
