# Supplementary material for: Two Frequenins in Drosophila: unveiling the evolutionary history of an unusual Neuronal Calcium Sensor (NCS) duplication
Source: BMC Evol Biol. 2010 Feb 19;10:54. doi: 10.1186/1471-2148-10-54 (PMC2837045; doi:10.1186/1471-2148-10-54)
Supplement: Additional file 5 — Data set of vertebrate NCS family sequences. Data set of vertebrate NCS sequences used in the functional divergence analysis. Species names are in the same four-letter format as in pre-computed alignments downloaded from ENSEMBL (except for Frequenin subfamily sequences, which are named as in Additional file 4. Data is in NBRF format. [file 1471-2148-10-54-S5.DOC]

>P1; HsapGCAP1

HsapGCAP1

MEGKSVEELSSTECHQWYKKFMTECPSGQLTLYEFRQFFGLKNLSPSASQYVEQMFETFDFNKDGYIDFMEYVAA

LSLVLKGKVEQKLRWYFKLYDVDGNGCIDRDELLTIIQAIRAINPCSDTTMTAEEFTDTVFSKIDVNGDGELSLE

EFIEGVQKDQMLLDTLTRSLDLTRIVRRLQNGEQDEEGADEAAEAAG*

>P1; TtruGCAP1

TtruGCAP1

IDGKSVEELSSTECHQWYKKFMTECPSGQLTFYEFRQFFGLKNLSPWASQYVEQMFETFDFNKDGYIDFMEYVAA

LSLVLKGKVEQKLRWYFKLYDVDGNGCIDRDELLTIIQAIRTINPCSDSTMTAEEFTDTVFSKIDVNGDGELSLE

EFMEGVQKDKMLLDTLTRSLDLTRIVRRLQNGEQDEEGAGGRESEAAEAAG*

>P1; CfamGCAP1

CfamGCAP1

MDGKSVEELSSTECHQWYKKFMTECPSGQLTLYEFRQFFGLKNLSPSASQYVEQMFETFDFNKDGYIDFMEYVAA

LSLVLKGKVEQKLRWYFKLYDVDGNGCIDRDELLTIIRAIRAINPCSDSTMSAEEFTNTVFSKIDVNGDGELSLE

EFIEGVQKDQMLLDTLTRSLDLTRIVRRLQNGEQEEEGAEGAGGGGTEAAG*

>P1; OcunGCAP1

OcunGCAP1

VEGKSVEELSSTECHQWYKKFMTECPSGQLTLYEFRQFFGLKNLSPTASQYVEQMFETFDFNKDGYIDFMEYVAA

LSLVLKEKVEHKLRWYFKLYDVDGNGCIDRDELLTIIQAIRTINPWSDSTMTAEEFTDTVFSKIDVNGDGELSLE

EFMEGVQKDQMLLDTLTQSDTRIVRRLQNDEDEEGAGTEKPKAAG*

>P1; TnigGCAP1

TnigGCAP1

TFGSLEELQACESHQWYKKFMTECPSGLLTFYEFKQFFGLKNLSPSSNAYIQTMFKTFDMNDDGFIDFMEYVAAL

SLVLKAGVQQKLRWYFKLYDIDGSGCIDRGELLIIIKSIRAINGIPSEMTSEEFANMVFDKIDVNGDGELSYEEF

MEGIQKDETLLKMLTESLDLTHIVQRIEGEMNTS*

>P1; BtauGCAP1

BtauGCAP1

MDGKSVEELSSTECHQWYKKFMTECPSGQLTLYEFRQFFGLKNLSPWASQYVEQMFETFDFNKDGYIDFMEYVAA

LSLVLKGKVEQKLRWYFKLYDVDGNGCIDRDELLTIIRAIRAINPCSDSTMTAEEFTDTVFSKIDVNGDGELSLE

EFMEGVQKDQMLLDTLTRSLDLTRIVRRLQNGEQDEEGASGRETEAAEADG*

>P1; RnorGCAP1

RnorGCAP1

VEGKSVEELSSTECHQWYKKFMTECPSGQLTLYEFRQFFGLKNLSPSASQYVEQMFETFDFNKDGYIDFMEYVAA

LSLVLKGKVEQKLRWYFKLYDVDGNGCIDRDELLTIIRAIRTINPWSDSSMSAEEFTDTVFAKIDINGDGELSLE

EFMEGVQKDQMLLDTLTRSLDLTRIVRRLQNGEQEEAGAGDLAAEAAG*

>P1; MmurGCAP1

MmurGCAP1

MDGKSVEELSSTECHQWYKKFMTECPSGQLTLYEFRQFFGLKNLSPSASQYVEQMFETFDFNKDGYIDFMEYVAA

LSLVLKGKVEQKLRWYFKLYDVDGNGCIDRDELLTIIQAIRAINPCSDTTMSAEEFTDTVFSKIDVNGDGELSLE

EFVEGVQKDQMLLDTLTRSLDLTRIVRRLQNSEQDGEEDGDPATEAAG*

>P1; TrubGCAP1

TrubGCAP1

VFGSLEELQACESHQWYRKFMTECPSGLLTFYEFKQFFGLKNLSPSSNAYIQTLFRTFDMNDDGFIDFMEYVAAL

SLVLKAGVQQKLRWYFKLYDIDGSGCIDRDELLLIIKSIRAINGIPSEMSAEEFTNMVFDKIDINGDGELSYEEF

IEGIQNDETLLKMLTESLDLTHIMQKIEGSGSGSGSGSDRYVEMFF*

>P1; OanaGCAP1

OanaGCAP1

MGGKTVEELSSTECHQWYKKFMNECPSGQLTLHEFRHFFGLKNLSPTANEYVEQMFETFDFNKDGHIDFMEYVAA

LSLVLKGKVEQKLRWYFKLYDVDGNGCIDRGELLTIIQAIRAINPCNDSTMTAEEFTDMVFTKIDINGDGELSLE

EFMEGVQKDEMLLDTLTRSLDLTHIVRMIQDEEQDKEEAAGRRKEGVSSPP*

>P1; AcarGCAP1

AcarGCAP1

MDGKSVEELSATECHQWYKKFMTECPSGQLTLHEFKQFFGLKNLTPASNEYIEQMFETFDFNKDGYIDFMEYVAA

LSLVLKGKVEQKLRWYFKLYDVDGNGCIDRDELLNIIKAIRTINPCNEIMSAEEFTNMVFDKIDINGDGELSLEE

FMDGVQKDEVLLEILTRSLDLTHIVRKIQNDGKNPDEGGTAEAAQ*

>P1; MdomGCAP1

MdomGCAP1

MEGKTVEELSSTECHQWYKKFMTECPSGQLTLYEFRQFFGLKNLSPSASQYVEQMFETFDFNKDGYIDFMEYVAA

LSLVLKGKVEQKLRWYFKLYDVDGNGCIDRGELLNIIRAIRAINPCSDSTMSAEEFTETVFAKIDVNGDGELSLE

EFMEGVQKDEMLLDTLTRSLDLTHIVRMIQNGGQSEDQGQGEDGAAG*

>P1; GgorGCAP1

GgorGCAP1

MEGKSVEELSSTECHQWYKKFMTECPSGQLTLYEFRQFFGLKNLSPSASQYVEQMFETFDFNKDGYIDFMEYVAA

LSLVLKGKVEQKLRWYFKLYDVDGNGCIDRDELLTIIQAIRAINPCSDSTMTAEEFTDTVFSKIDVNGDGELSLE

EFIEGVQKDQMLLDTLTRSLDLTRIVRRLQNGEQDEEGADEAAEAAG*

>P1; PtroGCAP1

PtroGCAP1

MEGKSVEELSSTECHQWYKKFMTECPSGQLTLYEFRQFFGLKNLSPSASQYVEQMFETFDFNKDGYIDFMEYVAA

LSVLKGKWYFLYDVDGNGCIDRHELLTIIQAIRAINPCSDTTMTAEEFTDTVFSKIDVNGDGELSLEEFIEGVQK

DQMLLDTLTRSLDLTRIVRRLQNGEQDEEGADEAAEAAG*

>P1; GgalGCAP1

GgalGCAP1

MDGKAVEELSATECHQWYKKFMTECPSGQLTLYEFKQFFGLKNLSPSANKYVEQMFETFDFNKDGYIDFMEYVAA

LSLVLKGKVDQKLRWYFKLYDVDGNGCIDRGELLNIIKAIRAINRCNEAMTAEEFTNMVFDKIDINGDGELSLEE

FMEGVQKDEVLLDILTRSLDLTHIVKLIQNDGKNPHAPEEAEEAAQ*

>P1; CporGCAP1

CporGCAP1

VEGKSVEELSSTECHQWYKKFMTECPSGQLTFYEFRQFFGLKNLSPSATEYVEQMFETFDFNKDGYIDFMEYVAA

LSLVLKGKVEQKLRWYFKLYDVDGNGCIDREELLTIIQAIRTINPWSDSGMSAEEFTNTVFAKIDVNGDGELSLE

EFIEGVQKDQMLLDTLTRSLDLTRIVRRLQNGEQDEAPGEGAGAAG*

>P1; TgutGCAP1

TgutGCAP1

MDGKTVEELSTTECHQWYKKFMTECPSGQLTLYEFKQFFGLKNLSPAANKYVEQMFETFDFNKDGYIDFMEYVAA

LSLVLKGKVDQKLRWYFKLYDVDGNGCIDRAELLNIIKAIRSINRCNETMTAEEFTDMVFNKIDINGDGELSLEE

FMEGVQKDEMLLDILTRSLDLTHIVRLIQNDGKNPHEGGEDAQAAP*

>P1; MmusGCAP1

MmusGCAP1

MEGKSVEELSSTECHQWYKKFMTECPSGQLTLYEFRQFFGLKNLSPSASQYVEQMFETFDFNKDGYIDFMEYVAA

LSLVLKGKVEQKLRWYFKLYDVDGNGCIDRDELLTIIRAIRTINPWSDSSMSAEEFTDTVFAKIDINGDGELSLE

EFMEGVQKDQMLLDTLTRSLDLTGIVRRLQNGEHEEAGTGDLAAEAAG*

>P1; TbelGCAP1

TbelGCAP1

MEGKSVEELSSTECHQWYKKFMTECSGQLTLYEFRQFFGLKNLSPTSSQYVEQMFETFDFNKDGYIDFMEYVAAL

SLVLKGKVEQKLRWYFKLYDVDGNGCIDREELLTIIQAIRTINPCSDTTMTAEEFTDTVFSKIDVNGDGELSLEE

FMEGIQKDQVLLDTLTRSLDLTRIVRRLQNGEDEEEEEAGEGTAKAAG*

>P1; DrerGCAP1

DrerGCAP1

SSSMSATELSACKCHQWYRKFMTECPSGQLTFYEFKKFFGLKNLSEKSNAYVNTMFKTFDIDDDGCIDFMEYVAA

LSLVLKGGVQQKLRWYFKLFDMDGSGCIDKDELLLIFKAVQAINGAEPEISAEDLADMVFNKIDVNGDGNLSVFE

FMEGISADEKISEMLTQSLDLTRIVSNIYNDSYIEQEAEIIEDQA*

>P1; XtroGCAP1

XtroGCAP1

MDGKAVEELSATEIHQWYKKFMTECPSGQLTQHEFKQFFNLKNLSPASNQYIEQMFNTFDFNKDGYMDFMEYVAA

LSLVLKGKVEQKLKWYFKLYDVDGNGCIDRGELLNIIKAIRAINRCNEDMTAEEFTDMVFDKIDINGDGELSLEE

FIEGVQRDEFLLNVLTRSLDLKHIVHMIQNDGQSIHTESTRQEIINGNIH*

>P1; PpygGCAP1

PpygGCAP1

MEGKSVEELSSTECHQWYKKFMTECPSGQLTLYEFRQFFGLKNLSPSASQYVEQMFETFDFNKDGYIDFMEYVAA

LSLVLKGKVEQKLRWYFKLYDVDGNGCIDRDELLTIIQAIRAINPCSDTTMTAEEFTDTVFSKIDVNGDGELSLE

EFIEGVQKDQMLLDTLTRSLDLTRIVRRLQNGEQDEEGADQAAEAAG*

>P1; OgarGCAP1

OgarGCAP1

MEGKSVEELSSTECHQWYKKFMTECPSGQLTLYEFRQFFGLKNLSPSASQYVEQMFETFDFNKDGYIDFMEYVAA

LSLVLKGKVEHKLRWYFKLYDVDGNGCIDRDELLTIIQAIRAINPCSDTTMSAEEFTDTVFSKIDVNGDGELSLE

EFMEGVQKDQMLLDTLTRSLDLTRIVRRLQNSQEDEEGDGDGAAEAAG*

>P1; MmulGCAP1

MmulGCAP1

MEGKSVEELSSTECHQWYKKFMTECPSGQLTLYEFRQFFGLKNLSPSASQYVEQMFETFDFNKDGYIDFMEYVAA

LSLVLKGKVEQKLRWYFKLYDVDGNGCIDRDELLTIIQAIRAINPCSDTAMTAEEFTDTVFSKIDVNGDGELSLE

EFIEGVQKDQMLLDTLTRSLDLTRIVRRLQNGEQDEEGADKEAAEAAG*

>P1; PvamGCAP1

PvamGCAP1

MEGKSVEELSSTECHQWYKKFMTECPSGQLTLYEFRQFFGLKNLSPSTSQYVEQMFETFDFNKDGYIDFMEYVAA

LSLVLKGKVEQKLRWYFKLYDVDGNGCIDRDELLTIIRAIRTINPYSDTTMSAEEFTDTVFSKIDVNGDGELSLE

EFMEGVQKDQMLLETLTRSLDLTSIVRRLQNGEQDEGAGGGAAEAAG*

>P1; LafrGCAP2

LafrGCAP2

MGQQFSWEEVEEMDMAELQEWYKKFVVECPSGTLFMHEFKRFFKVTDNEEATQYVEGMFRAFDKNGDNTIDFLEY

VAALNLVLRGTLEHKLKWTFKIYDKDRDGCIDRLELLDIVEAIYKMKKACQVEMEAEQQGQLLTPEEVVDRIFLL

VDENGDGQLSLTEFLEGARRDKWVMKMLHMDVSPGSWVSQQRRRSAMF*

>P1; EeurGCAP2

EeurGCAP2

MGQQFSWEEAEEMDVAALQEWYKKFVVECPSGTLFMHEFKRFFKVTGNEEASQYVEGMFRAFDKNGDNTIDFLEY

VAALNLVLRGTLEHKLKWTFKIYDKDRNGCIDRLELLDIVEAIYKLKKACRIETEGEQYQMLTPEVVDRIFLLVD

ENGDGRLSLNEFIEGARRDKWVMKMLQMDMNPGSWISQQRRKSAMF*

>P1; OlatGCAP2

OlatGCAP2

MGQRLSEESDPEIDVAELQEWYKKFVVECPSGTLFMHEFKGFFGVTNNKEAADYIENMFRAFDKNGDNTIDFLEY

VAALNLVLRGKLEHKLKWTFKMYDKDGSGCIDKTELLEIVESIYRLKKACHGELDEECNLLTPDQVVDRIFELVD

ENGDGELSLDEFIDGARRDKWVMKMLQMDVNPGDWINERRCSEDF*

>P1; CfamGCAP2

CfamGCAP2

MGQQFSWEEAEEMDVAELQEWYKKFVVECPSGSLFMHEFKRFFKVTGNEEATQYVEGMFRAFDKNGDNTIDFLEY

VAALNLVLRGTLEHKLKWTFKIYDKDRNGCIDRLELLDIVEAIYKLKKACRVEMESEQQGQLLTPEEVVDRIFLL

VDENGDGNLSLNEFIEGARRDKWVMKMLQMDVNPGGWISQQRRRSAMF*

>P1; MmulGCAP2

MmulGCAP2

MGQEFSWEEAEEIDVAELQEWYKKFVMECPSGTLFMHEFKRFFKVTDDEEASQYVEGMFRAFDKNGDNTIDFLEY

VAALNLVLRGTLEHKLKWTFKIYDKDGNGCIDRLELLNIVEGIYQLKKACRRELQTEQGQLLTPEEVVDRIFLLV

DENGDGQLSLNEFVEGARRDKWVMKMLQMDMNPSSWLAQQRRKSAMF*

>P1; RnorGCAP2

RnorGCAP2

MGQQFSWEEAEEMDVAELQEWYKKFVVECPSGTLFMHEFKRFFKVTGNEEATQYVEGMFRAFDKNGDNTIDFLEY

VAALNLVLRGTLEHKLKWTFKIYDKDRNGCIDRLELLDIVEAIYKLKKACRAELDLEQQGQLLTPEEVVDRIFLL

VDENGDGQLSLTEFIEGARRDKWVMKMLQMDVNPGGWITQQRRRSAMF*

>P1; AcarGCAP2

AcarGCAP2

MGQHFTNEEGEDIDAAELQEWYKKFVVECPSGTLFMHEFKHFFGVQNDEQAAEYVENMFKAFDKNGDNTIDFLEY

VAALNLVLRGKLEHKLRWTFKIYDKDGNGCIDKPELLEIIESIYRLKQICQTEGEKGQPSLTPEEVVERIFELVD

ENGDGQLSLDEFIDGARKDRWVMKMLQMDGNAGVWITERRRKSAMF*

>P1; PtroGCAP2

PtroGCAP2

MGQEFSWEEAEEIDVAELQEWYKKFVMECPSGTLFMHEFKRFFKVTDDEEASQYVEGMFRAFDKNGDNTIDFLEY

VAALNLVLRGTLEHKLKWTFKIYDKDGNGCIDRLELLNIVEGIYQLKKACRRELQTEQGQLLTPEEVVDRIFLLV

DENGDGQLSLNEFVEGARRDKWVMKMLQMDMNPSSWLAQQRRKSAMF*

>P1; DrerGCAP2

DrerGCAP2

MGQRLSDDSDEIDVAELQEWYKKFVIECPSGTLFMHEFKSFFGVTENPEAADYIENMFRAFDKNGDNTIDFLEYV

AALNLVLRGKLEHKLKWTFKMYDKDGSGCIDKTELKEIVESIYRLKKACHGELDAECNLLTPDQVVDRIFELVDE

NGDGELSLDEFIDGARRDKWVMKMLQMDVNPGDWINEQRRRSANF*

>P1; TgutGCAP2

TgutGCAP2

MGQQFTNAEGEEIDVAELQEWYKKFVVECPSGTLFMHEFKRFFGVQDNQEAAEYVENMFRAFDKNGDNTIDFLEY

VAALNLVLRGKLEHKLRWTFKVYDKDGNGCIDKPELLEIVESIYRLKKVCWSEVEDRTPLLTPEEVVDRIFQLVD

ENGDGQLSLDEFIDGARKDKWVMKMLQMDVNPGGWITEQRRKSALF*

>P1; TtruGCAP2

TtruGCAP2

MGQQFSGEEAEVLDVAELQEWYRKFLEECPSGTLFLHEFKRFFKVAGNEEASQYVEGMFRAFDKNGDDTIDFLEY

VAALNLVLRGTLEHKLKWTFKIYDKDRNGFLDRLELLDIVESIYKLKKACRVEMEAEWQGKLLTPEEVVDRIFLL

VDENGDGQLSLNEFIEGARRDKWVMKMLQMDVNPGSWISQQRRKSAVF*

>P1; FcatGCAP2

FcatGCAP2

MGQQFSLEEAKEMDVAELQEWYKKFVVECPSGSLFMHEFKRFFKVTGNEEATQYVEGMFRAFDKNGDNTIDFLEY

VAALNLVLRGTLEHKLKWTFKIYDKDRNGCIDRLELLDIVEAIYKLKKACRVEEEAEQQGQLLTPEEVVDRIFLL

VDENGDGQLSLNEFIEGARRDKWVMKMLQMDMNPGGWISQQRRKSAMF*

>P1; BtauGCAP2

BtauGCAP2

MGQQFSWEEVEAADAAQLQEWYKKFLEECPSGTLFMHEFKRFFKVPDNEEATQYVEAMFRAFDTNGDNTIDFLEY

VAALNLVLRGTLEHKLKWTFKIYDKDRNGCIDRQELLDIVESIYKLKKACSVEVEAEQQGKLLTPEEVVDRIFLL

VDENGDGQLSLNEFVEGARRDKWVMKMLQMDLNPSSWISQQRRKSAMF*

>P1; MmusGCAP2

MmusGCAP2

MGQQLSWEEAEEMDVAELQEWYKKFVVECPSGTLFMHEFKRFFKVTGNEEASQYVESMFRAFDKNGDNTIDFLEY

VAALNLVLRGSLEHKLKWTFKIYDKDRNGCIDRLELLDIVEAIYKLKKACRAELDLEHQGQLLTPEEVVDRIFLL

VDENGDGQLSLTEFIEGARRDKWVMKMLQMDINPGCWITQQRRRSAMF*

>P1; EcabGCAP2

EcabGCAP2

MGQQFSWEEVEEMDVAELQEWYKKFVVECPSGTLFMHEFKRFFKVAGDEEATQYVEGMFRAFDKNGDNTIDFLEY

VAALNLVLRGTLEHKLKWTFKIYDKDRNGCIDRLELLDIVEAIYKLKKACRVEMEAEQQSQLLTPEEVVDRIFLL

VDENGDGQLSLNEFIEGARRDKWVMKMLQLDVNPSGWISQQRRKSAMF*

>P1; GacuGCAP2

GacuGCAP2

MGQRLSEESDPEIDVAELQEWYKKFVVECPSGTLFMHEFKGFFGVADNKEAADYIENMFRAFDKNGDNTIDFLEY

VAALNLVLRGKLEHKLKWTFKMYDKDGSGCIDKTELLEIVESIYRLKKACHGDLDEECHLLTPDQVVDRIFELVD

ENGDGELSLDEFIDGARRDKWVMKMLQMDVNPGDWMNEQSRSSDF*

>P1; GgalGCAP2

GgalGCAP2

MGQQFTNAEGEEIDVAELQEWYKKFVVECPSGTLFMHEFKRFFGVQDNHEAAEYIENMFRAFDKNGDNTIDFLEY

VAALNLVLRGKLEHKLRWTFKVYDKDGNGCIDKPELLEIVESIYKLKKVCRSEVEERTPLLTPEEVVDRIFQLVD

ENGDGQLSLDEFIDGARKDKWVMKMLQMDVNPGGWISEQRRKSALF*

>P1; TrubGCAP2

TrubGCAP2

MGQRLSEESDPEIDVAELQEWYKKFVVECPSGTLFMHEFKGFFGVTENKEAADYIENMFRAFDKNGDNTIDFLEY

VAALNLVLRGKLEHKLKWTFKMYDKDGSGCIDKTELLEIVESIYRLKKACHGELDEDCTLLTPDQVVDRIFELVD

ENGDGELSLDEFIDGARRDKWVMKMLQMDVNPGDWLNDRRRSADF*

>P1; HsapGCAP2

HsapGCAP2

MGQEFSWEEAEEIDVAELQEWYKKFVMECPSGTLFMHEFKRFFKVTDDEEASQYVEGMFRAFDKNGDNTIDFLEY

VAALNLVLRGTLEHKLKWTFKIYDKDGNGCIDRLELLNIVEGIYQLKKACRRELQTEQGQLLTPEEVVDRIFLLV

DENGDGQLSLNEFVEGARRDKWVMKMLQMDMNPSSWLAQQRRKSAMF*

>P1; CpoxGCAP2

CpoxGCAP2

MGQQFSWEEGREMDVAELQEWYKRFLMECPSGTLFMHEFKRLFKVTGDDDASQYVEGMFRAFDKNGDNTIDFLEY

VAALNLVLRGSLEHKLRWTFKIYDKDRNGCIDRAELLDIVESIYRLKKACRMETDMAKDGQLLSPEEVVDRIFLL

VDENGDGQLSLDEFIKGARRDQWVMKMLQIDVNPGSWITQQRRKSAMF*

>P1; DrerGCAP3

DrerGCAP3

DMHHWYNKFMRESPSGLITLFELKSILGLQGMNEDANSYVDQVFCTFDMDRDGYIDFVEYIAAISLMLKGEINQK

LKWYFKLFDQDGNGKIDKDELETIFTAIQDITRNRDIVPEEIVALIFEKIDVNGEGELTLEEFIEGAKEHPEIMD

MLKILMDLTPVLLII*

>P1; OlatGCAP3

OlatGCAP3

DMHHWYTKFMRESPSGLITLFELKTMLEMNGMTEEASSYVDQVFFTFDMDGDGYIDFVEYIAAISLLLKGEINQK

LKWYFKLFDQDGNGKIDKDELETIFKAIQDITRSYDIPPEEIVTLIYEKIDVKGEGELTLEEFISGAREHPDIME

MLTKMMDLTHVLEII*

>P1; EcabGCAP3

EcabGCAP3

DTYMWYRKFMMEYPSGLQTLHEFKALLGLQGLDQKANQHIDQVYNTFDMNKDGFIDFLEFIAAINLVVRGKVEQK

LKWYFKLYDADGNGSIDRKELLNIFMAVQALNGQQTLSPEEFTNLVFHKVDVNNDGELSLKEFISGTESDQDLLE

IVSKSFDFSSVLKVI*

>P1; CfamGCAP3

CfamGCAP3

ETYVWYRKFMREYPSGLQTLHEFKTLLGLQGLNPKANQHVDQVYNTFDMNKDGFIDFLEFIAAINLVIRGKMDQK

LKWYFKLYDADGNGSIDKKELLNIFMAVQALNGQQTLSPEEFTNLVFNKIDINNDGELTLEEFINGTEKDQDLLD

IVSKSFDFSNVLKVI*

>P1; TrubGCAP3

TrubGCAP3

DMHHWYTKFMRESPSGLITLFELKTILEMNGMTEEASSYVDQVFYTFDMDGDGYIDFVEYIAAISLLLKGEINQK

LKWYFKLFDQDGNGKIDKDELETIFKAIQDITRTYDIPPEEIVTLIYDKIDVNGEGELTLEEFISGAKEHPDIME

MLTKMMDLSHVLEII*

>P1; PpygGCAP3

PpygGCAP3

ETHVWYRTFMMEYPSGLQTLHEFKTLLGLQGLNQKANKHIDQVYNTFDTNKDGFIDFLEFIAAVNLIVQEKMEQK

LKWYFKLYDADGNGSIDKNELLDMFMAVQALNGQQTLSPEEFINLVFHKIDINNDGELTLEEFINGMAKDQDLLE

IVYKSFDFSNVLRVI*

>P1; MmulGCAP3

MmulGCAP3

GTHVWYRTFMTEYPSGLQTLHEFKTLLGLQGLNQKANKHVDQVYNTFDTNKDGFIDFLEFIAAVNLIVQEKMEQK

LKWYFKLYDVDGNGSIDKNELLDMFVAVQALNGQQTLSPEEFTNLVFRKIDINNDGELTLEEFINGIAKDQDLLE

IVYKSFDLSNVLRVI*

>P1; GacuGCAP3

GacuGCAP3

DMHHWYNKFMRESPSGLITLFELKAILGLKGMTEDANSYVDQVFLTFDMDGDGYIDFVEYIAAISLMLKGEINQK

LKWYFKLFDQDGNGKIDKEELETIFTEVQDITRNRDTDSEDIVSIIFEKIDVKGEGKKRGGEVIEEPRPRDIMDM

LKSLDLTPVLVII*

>P1; GgalGCAP3

GgalGCAP3

EMHHYYSKFMRECPSGQLSLHEFKKLLGLQGLDPQGDLYIKRVFDIFDLNQDGFIDFLEFIAAINLVIRGKIDQK

LKWYFKLYDADGNGCIDKKELLSIFAAIQAINGQTNMTAEEFTNMIFQKIDVNNDGELTLEEFITGVERDEDLME

LITKSFDLSNVIKVI*

>P1; TgutGCAP3

TgutGCAP3

EIHHWYTKFMKECPSGQLCLHEFKHVLDLHGLTPEANNYVEQVFHTFDMNKDGFIDFLEFIAAINLVIRGKIDQK

LKWYFKLYDADGNGCIDKKELLSIFRAIQAINGYTNMSAEEFTNMIFQKIDVNNDGELTLEEFINGVEKDEDLME

LITKSFDLSNVLKVI*

>P1; PvamGCAP3

PvamGCAP3

ETYVWYKKFMMEYPSGLQTLHEFKTLLGLQGLNQKANQHVDQVYNTFDMNKDGFIDFLEFIAAVNLVVRGKMEQK

LKWYFKLYDADGNGSIDEKELLNIFMTVQTLNGQQTLSPEEFTSLVFHKIDINHDGELTLEEFIDGTKKDQDLLK

IVSKSFDFSSVLKLI*

>P1; ChofGCAP3

ChofGCAP3

ETYVWYRKFMLGYPSSLQTLHRFKKYLLQGLNQKANQHVDQVNNTFDMNKDGFIDFLVFIASVNLVVGKVEKLKW

HFKLYDADGNGSIDKKELLNIFIAVQALNSQQTPSPEEFTNLVFHKFIVNNDGELILKELINGTENDQDLLETVS

KSLDFSNVLKAI*

>P1; VpacGCAP3

VpacGCAP3

ETYVWYGGFMMEPPGLQTLHEFKTLLGLQDLNQKASQQVDQVYNTFDRNKNGFADFSEFIAAVNLVAQGKKKKKL

KCYFKLYVVDGNGSTDKRELLNILMVIALNGQQTLSPEEFTKLVFHIHIYENRELTLEELSNGTEKDQGLLEIET

LKLFNVLRVI*

>P1; TsyrGCAP3

TsyrGCAP3

ETYMWYRTFMMEYPSGLQTLHEFKTLLGLQGLNQKANKHIDQVYNTFDMNKDGFIDFLEFIAAVNLIVREKLEQK

LKWYFKLYDTDGNGSIDKNELLDMFMAVQALNGQQTLSPEEFTNLVFHKIDINNDGELTLEEFINGMEKDQDLLE

IVYKSFDLSNVLRVI*

>P1; TnigGCAP3

TnigGCAP3

DMHHWYNKFMRESPSGLITLFELKAILNLKGMNENANSYVDQVFFTFDMDGDGYIDFVEYIAAISLLLKGEINQK

LKWYFKLFDQDGNGKIDREELETIFSAIQDITRNKDIDPEEIVSLIFERIDVNGEGELTLEEFIEGAKDHQDIMD

MLKKIMDLTPVLVII*

>P1; XtroGCAP3

XtroGCAP3

ELHKWYGKFMKECPSGQLSLHEFKELLGLQGMNFEANRYIDQVFSTFDMNKDGFIDFLEFIAAINLVLRGKIDQK

LKWYFKLYDADGNGSIDRKELLSILTAVRAINGHRGMSPEEFTSMVFEKIDVNGDGELTLEEFINGIEKDEQLLE

IISKTFDLSNVLKTI*

>P1; MdomGCAP3

MdomGCAP3

ETHVWFKRFMTECPSGLQTLHEFKALLGLQGLNQRANQYVDQLFNIFDKNKDGFIDFLEFIAAVNLVIRGKMDQK

LKWYFKLYDADGNGSIDRKELLNIFTAIQAINGQNTLNPQEFANLIFEKTDTNNDGELTLEEFISGLEKDPHLLE

MISKSFDLSNVLKII*

>P1; AcarGCAP3

AcarGCAP3

EIHHWYTKFMRECPSGQLCLHEFKSVLGLQGLNEEANKYVEQVFETFDMNKDGFIDFLEFIAAINLVIRGKIDQK

LKWYFKLYDADGNGTIDKKELISIFTAIQAINGNQELSPEDFANVVFQKIDINNDGELTLDEFINGVESDEYLLE

MISKSFDLSNVLKVI*

>P1; HsapGCAP3

HsapGCAP3

ETHVWYRTFMMEYPSGLQTLHEFKTLLGLQGLNQKANKHIDQVYNTFDTNKDGFVDFLEFIAAVNLIMQEKMEQK

LKWYFKLYDADGNGSIDKNELLDMFMAVQALNGQQTLSPEEFINLVFHKIDINNDGELTLEEFINGMAKDQDLLE

IVYKSFDFSNVLRVI*

>P1; PtroVILIP2

PtroVILIP2

MGKQNSKLRPEVLQDLRENTEFTDHELQEWYKGFLKDCPTGHLTVDEFKKIYANFFPYGDASKFAEHVFRTFDTN

GDGTIDFREFIIALSVTSRGKLEQKLKWAFSMYDLDGNGYISRSEMLEIVQAIYKMVSSVMKMPEDESTPEKRTD

KIFRQMDTNNDGKLSLEEFIKGAKSDPSIVRLLQCDPSSASQF*

>P1; GgalVILIP2

GgalVILIP2

MGKQNSKLRPEVLQDLRENTEFTDHELQEWYKGFLKDCPTGHLTVEEFKKIYANFFPYGDASKFAEHVFRTFDTN

GDGTIDFREFIIALSVTSRGKLEQKLKWAFSMYDLDGNGYISRGEMLEIVQAIYKMVSSVMKMPEDESTPEKRTD

KIFRQMDTNNDGKLSLEEFIKGAKSDPSIVRLLQCDPSSASQF*

>P1; MdomVILIP2

MdomVILIP2

MGKQNSKLRPEVLQDLRENTEFTDHELQEWYKGFLKDCPTGHLTVDEFKKIYANFFPYGDASKFAEHVFRTFDTN

GDGTIDFREFIIALSVTSRGKLEQKLKWAFSMYDLDGNGYISRGEMLEIVQAIYKMVSSVMKMPEDESTPEKRTD

KIFRQMDTNNDGKLSLEEFIKGAKSDPSIVRLLQCDPSSASQF*

>P1; StriVILIP2

StriVILIP2

MGKQNSKLRPEVLQDLRENTEFTDHELQEWYKGFLKDCPTGHLTVDEFKKIYANFFPYGDASKFAEHVFRTFDTN

GDGTIDFREFIIALSVTSRGKLEQKLKWAFSMYDLDGNGYISRSEMLEIVQAIYKMVSSVMKMPEDESTPEKRTD

KIFRQMDTNNDGKLSLEEFIKGAKSDPSIVRLLQCDPSSASQF*

>P1; LafrVILIP2

LafrVILIP2

MGKQNSKLRPEVLQDLRENTEFTDHELQEWYKGFLKDCPTGHLTVDEFKKIYANFFPYGDASKFAEHVFRTFDTN

GDGTIDFREFIIALSVTSRGKLEQKLKWAFSMYDLDGNGYISRSEMLEIVQAIYKMVSSVMKMPEDESTPEKRTD

KIFRQMDTNNDGKLSLEEFIKGAKSDPSIVRLLQCDPSSASQF*

>P1; MmurVILIP2

MmurVILIP2

MGKQNSKLRPEVLQDLRENTEFTDHELQEWYKGFLKDCPTGHLTVDEFKKIYANFFPYGDASKFAEHVFRTFDTN

GDGTIDFREFIIALSVTSRGKLEQKLKWAFSMYDLDGNGYISRSEMLEIVQAIYKMVSSVMKMPEDESTPEKRTD

KIFRQMDTNNDEFIKGAKSDPSIVRLLQCDPSSASQF*

>P1; OpriVILIP2

OpriVILIP2

MGKQNSKLRPEVLQDLRENTEFTDHELQEWYKGFLKDCPTGHLTVDEFKKIYANFFPYGDASKFAEHVFRTFDTN

GDGTIDFREFIIALSVTSRGKLEQKLKWAFSMYDLDGNGYISRSEMLEIVQAIYKMVSSVMKMPEDESTPEKRTD

KIFRQMDTNNDGKLSLEEFIKGAKSDPSIVRLLQCDPSSASQF*

>P1; AcarVILIP2

AcarVILIP2

MGKQNSKLRPEVLQDLRENTEFTDHELQEWYKGFLKDCPTGHLTVDEFKKIYANFFPYGDASKFAEHVFRTFDTN

GDGTIDFREFIIALSVTSRGKLEQKLKWAFSMYDLDGNGYISRAEMLEIVQAIYKMVSSVMKMPEDESTPEKRTD

KIFRQMDTNNDGKLSLEEFIKGAKSDPSIVRLLQCDPSSASQF*

>P1; PpygVILIP2

PpygVILIP2

MGKQNSKLRPEVLQDLRENTEFTDHELQEWYKGFLKDCPTGHLTVDEFKKIYANFFPYGDASKFAEHVFRTFDTN

GDGTIDFREFIIALSVTSRGKLEQKLKWAFSMYDLDGNGYISRSEMLEIVQAIYKMVSSVMKMPEDESTPEKRTD

KIFRQMDTNNDGKLSLEEFIKGAKSDPSIVRLLQCDPSSASQF*

>P1; OgarVILIP2

OgarVILIP2

MGKQNSKLRPEVLQDLRENTEFTDHELQEWYKGFLKDCPTGHLTVDEFKKIYANFFPYGDASKFAEHVFRTFDTN

GDGTIDFREFIIALSVTSRGKLEQKLKWAFSMYDLDGNGYISRSEMLEIVQAIYKMVSSVMKMPEDESTPEKRTD

KIFRQMDTNNDGKLSLEEFIKGAKSDPSIVRLLQCDPSSASQF*

>P1; TtruVILIP2

TtruVILIP2

MGKQNSKLRPEVLQDLRENTEFTDHELQEWYKGFLKDCPTGHLTVDEFKKIYANFFPYGDASKFAEHVFRTFDTN

GDGTIDFREFIIALSVTSRGKLEQKLKWAFSMYDLDGNGYISRSEMLEIVQAIYKMVSSVMKMPEDESTPEKRTD

KIFRQMDTNNDGKLSLEEFIKGAKSDPSIVRLLQCDPSSASQF*

>P1; EcabVILIP2

EcabVILIP2

MGKQNSKLRPEVLQDLRENTEFTDHELQEWYKGFLKDCPTGHLTVDEFKKIYANFFPYGDASKFAEHVFRTFDTN

GDGTIDFREFIIALSVTSRGKLEQKLKWAFSMYDLDGNGYISRSEMLEIVQAIYKMVSSVMKMPEDESTPEKRTD

KIFRQMDTNNDGKLSLEEFIKGAKSDPSIVRLLQCDPSSASQF*

>P1; CporVILIP2

CporVILIP2

MGKQNSKLRPEVLQDLRENTEFTDHELQEWYKGFLKDCPTGHLTVDEFKKIYANFFPYGDASKFAEHVFRTFDTN

GDGTIDFREFIIALSVTSRGKLEQKLKWAFSMYDLDGNGYISRSEMLEIVQAIYKMVSSVMKMPEDESTPEKRTD

KIFRQMDTNNDGKLSLEEFIKGAKSDPSIVRLLQCDPSSASQF*

>P1; TrubVILIP2

TrubVILIP2

MGKQNSKLRPEVLNDLRENTEFTDHELQEWYRGFLKDCPTGHLTVDEFKKIYANFFPYGDASKFAEHVFRTFDTN

GDATIDFREFIIALSVTSRGGLEQKLRWAFSMYDLDGNGYISRAEMLEIVQAIYKMVSSVMKMPEDESTPEKRTD

KIFRQMDTDNDGRLSLEEFIKGAKSDPSIVRLLQSDQGGSRQL*

>P1; RnorVILIP2

RnorVILIP2

MGKQNSKLRPEVLQDLREHTEFTDHELQEWYKGFLKDCPTGHLTVDEFKKIYANFFPYGDASKFAEHVFRTFDTN

SDGTIDFREFIIALSVTSRGKLEQKLKWAFSMYDLDGNGYISRSEMLEIVQAIYKMVSSVMKMPEDESTPEKRTD

KIFRQMDTNNDGKLSLEEFIKGAKSDPSIVRLLQCDPSSASQF*

>P1; DrerVILIP2

DrerVILIP2

MGKQNSKLRPEVLNDLRENTEFTDHELQEWYRGFLKDCPSGHLTVEEFKKIYANFFPYGDASKFAEHVFRTFDTN

SDATIDFREFIIALSVTSRGGLEQKLRWAFSMYDLDGNGYISRAEMLEIVQAIYKMVSSVMKMPEDESTPEKRTD

KIFRQMDTDNDGRLSLEEFIKGAKSDPSIVRLLQSDQSSSRQL*

>P1; TgutVILIP2

TgutVILIP2

MGKQNSKLRPEVLQDLRENTEFTDHELQEWYKGFLKDCPTGHLTVEEFKKIYANFFPYGDASKFAEHVFRTFDTN

GDGTIDFREFIIALSVTSRGKLEQKLKWAFSMYDLDGNGYISRGEMLEIVQAIYKMVSSVMKMPEDESTPEKRTD

KIFRQMDTNNDGKLSLEEFIKGAKSDPSIVRLLQCDPSSASQF*

>P1; TnigVILIP2

TnigVILIP2

MGKQNSKLRPEVLNDLRENTEFTDHELQEWYRGFLKDCPSGHLTVDEFKKIYANFFPYGDASKFAEHVFRTFDTN

GDATIDFREFIIALSVTSRGGLEQKLRWAFSMYDLDGNGYISREEMLEIVQAIYKMVSSVMKMPEDESTPEKRTD

KIFRQMDTDNDGRLSMEEFIKGAKSDPSIVRLLQSDQAGPR*

>P1; MmusVILIP2

MmusVILIP2

MGKQNSKLRPEVLQDLREHTEFTDHELQEWYKGFLKDCPTGHLTVDEFKKIYANFFPYGDASKFAEHVFRTFDTN

SDGTIDFREFIIALSVTSRGKLEQKLKWAFSMYDLDGNGYISRSEMLEIVQAIYKMVSSVMKMPEDESTPEKRTD

KIFRQMDTNNDGKLSLEEFIKGAKSDPSIVRLLQCDPSSASQF*

>P1; HsapVILIP2

HsapVILIP2

MGKQNSKLRPEVLQDLRENTEFTDHELQEWYKGFLKDCPTGHLTVDEFKKIYANFFPYGDASKFAEHVFRTFDTN

GDGTIDFREFIIALSVTSRGKLEQKLKWAFSMYDLDGNGYISRSEMLEIVQAIYKMVSSVMKMPEDESTPEKRTD

KIFRQMDTNNDGKLSLEEFIRGAKSDPSIVRLLQCDPSSASQF*

>P1; MmulVILIP2

MmulVILIP2

MGKQNSKLRPEVLQDLRENTEFTDHELQEWYKGFLKDCPTGHLTVDEFKKIYANFFPYGDASKFAEHVFRTFDTN

GDGTIDFREFIIALSVTSRGKLEQKLKWAFSMYDLDGNGYISRSEMLEIVQAIYKMVSSVMKMPEDESTPEKRTD

KIFRQMDTNNDGKLSLEEFIKGAKSDPSIVRLLQCDPSSASQF*

>P1; CfamVILIP2

CfamVILIP2

MGKQNSKLRPEVLQDLRENTEFTDHELQEWYKGFLKDCPTGHLTVDEFKKIYANFFPYGDASKFAEHVFRTFDTN

GDGTIDFREFIIALSVTSRGKLEQKLKWAFSMYDLDGNGYISRSEMLEIVQAIYKMVSSVMKMPEDESTPEKRTD

KIFRQMDTNNDGKLSLEEFIKGAKSDPSIVRLLQCDPSSASQF*

>P1; XtroVILIP2

XtroVILIP2

MGKQNSKLRPEVLQDLRENTEFTDHELQEWYKGFLKDCPTGHLTVEEFKKIYANFFPYGDASKFAEHVFRTFDTN

GDGTIDFREFIIALSVTSRGKLEQKLKWAFSMYDLDGNGYISRGEMLEIVQAIYKMVSSVMKMPEDESTPEKRTD

KIFKQMDTNNDGKLSLEEFIKGAKSDPSIVRLLQCDPSSTSQF*

>P1; BtauVILIP2

BtauVILIP2

MGKQNSKLRPEVLQDLRENTEFTDHELQEWYKGFLKDCPTGHLTVDEFKKIYANFFPYGDASKFAEHVFRTFDTN

GDGTIDFREFIIALSVTSRGKLEQKLKWAFSMYDLDGNGYISRSEMLEIVQAIYKMVSSVMKMPEDESTPEKRTD

KIFRQMDTNNDGKLSLEEFIKGAKSDPSIVRLLQCDPSSASQF*

>P1; GacuVILIP2

GacuVILIP2

MGKQNSKLRPEVLNDLRENTEFTDHELQEWYRGFLKDCPTGHLTVEEFKKIYANFFPYGDASKFAEHVFRTFDTN

GDATIDFREFIIALSVTSRGGLEQKLRWAFSMYDLDGNGYISRAEMLEIVQAIYKMVSSVMKMPEDESTPEKRTD

KIFRQMDIDNDGRLSLEEFIKGAKSDPSIVRLLQSDQGASCQF*

>P1; OlatVILIP2

OlatVILIP2

MGKQNSKLRPEVLNDLRENTEFTDNELQEWYRGFLKDCPTGHLTVEEFKKIYANFFPYGDASKFAEHVFRTFDTN

GDATIDFREFIIALSVTSRGGLEQKLRWAFSMYDLDGNGYISRAEMLEIVQAIYKMVSSVMKMPEDESTPEKRTD

KIFKQMDIDNDGRLSLEEFIKGAKSDPSIVRLLQSDQGTSRQI*

>P1; FcatVILIP2

FcatVILIP2

MGKQNSKLRPEVLQDLRENTEFTDHELQEWYKGFLKDCPTGHLTVDEFKKIYANFFPYGDASKFAEHVFRTFDTN

GDGTIDFREFIIALSVTSRGKLEQKLKWAFSMYDLDGNGYISRSEMLEIVQAIYKMVSSVMKMPEDESTPEKRTD

KIFRQMDTNNDGKLSLEEFIKGAKSDPSIVRLLQCDPSSASQF*

>P1; EtelVILIP3

EtelVILIP3

MGKTNSKLAPEVLEDLVQNTEFSEQELKQWYKGFLKDCPSGILNLEEFQQLYIKFFPYGDASKFAQHAFRTFDKN

GDGTIDFREFICALSVTSRGSFEQKLNWAFEMYDLDGDGRITRLEMLEIIEAIYKMVGTVIMMRMNQDGLTPQQR

VDKIFKKMDQDKDDQITLEEFKEAAKSDPSIVLLLQCDMQK*

>P1; CfamVILIP3

CfamVILIP3

MGKTNSKLAPEVLEDLVQNTEFSEQELKQWYKGFLKDCPSGILNLEEFQQLYIKFFPYGDASKFAQHAFRTFDKN

GDGTIDFREFICALSVTSRGSFEQKLNWAFEMYDLDGDGRITRLEMLEIIEAIYKMVGTVIMMRMNQDGLTPQQR

VDKIFKKMDQDKDDQITLEEFKEAAKSDPSIVLLLQCDMQK*

>P1; MdomVILIP3

MdomVILIP3

MGKGNSKLAPEVLEDLVQTTEFSEQELKQWYKGFLKDCPSGILNLEEFQQLYVKFFPYGDASKFAQHAFRTFDKN

GDGTIDFREFICALSVTSRGSFEQKLNWAFEMYDLDGDGRITRLEMLEIIEAIYKMVGTVIMMRMNQDGLTPQQR

VDKIFTKMDQDKDDQITLEEFKEAAKSDPSIVLLLQCDMQK*

>P1; RnorVILIP3

RnorVILIP3

MGKNNSKLAPEELEDLVQNTEFSEQELKQWYKGFLKDCPSGILNLEEFQQLYIKFFPYGDASKFAQHAFRTFDKN

GDGTIDFREFICALSVTSRGSFEQKLNWAFEMYDLDGDGRITRLEMLEIIEAIYKMVGTVIMMRMNQDGLTPQQR

VDKIFKKMDQDKDDQITLEEFKEAAKSDPSIVLLLQCDMQK*

>P1; MmulVILIP3

MmulVILIP3

MGKTNSKLAPEVLEDLVQNTEFSEQELKQWYKGFLKDCPSGILNLEEFQQLYIKGGRGGEAPGFAQHAFRTFDKN

GDGTIDFREFICALSVTSRGSFEQKLNWAFEMYDLDGDGRITRLEMLEIIEAIYKMVGTVIMMRMNQDGLTPQQR

VDKIFKKMDQDKDDQITLEEFKEAAKSDPSIVLLLQCDMQK*

>P1; BtauVILIP3

BtauVILIP3

MGKTNSKLAPEVLEDLVQNTEFSEQELKQWYKGFLKDCPSGILNLEEFQQLYIKFFPYGDASKFAQHAFRTFDKN

GDGTIDFREFICALSVTSRGSFEQKLNWAFEMYDLDGDGRITRLEMLEIIEAIYKMVGTVIMMRMNQDGLTPQQR

VDKIFKKMDQDKDDQITLEEFKEAAKSDPSIVLLLQCDMQK*

>P1; PvamVILIP3

PvamVILIP3

MGKTNSKLAPEVLEDLVQNTEFSEQELKQWYKGFLKDCPSGILNLEEFQQLYIKFFPYGDASKFAQHAFRTFDKN

GDGTIDFREFICALSVTSRGSFEQKLNWAFEMYDLDGDGRITRLEMLEIIEAIYKMVGTVIMMRMNQDGLTPQQR

VDKIFKKMDQDKDDQITLEEFKEAAKSDPSIVLLLQCDMQK*

>P1; PtroVILIP3

PtroVILIP3

MGKTNSKLAPEVLEDLVQNTEFSEQELKQWYKGFLKDCPSGILNLEEFQQLYIKFFPYGDASKFAQHAFRTFDKN

GDGTIDFREFICALSVTSRGSFEQKLNWAFEMYDLDGDGRITRLEMLEIIEAIYKMVGTVIMMRMNQDGLTPQQR

VDKIFKKMDQDKDDQITLEEFKEAAKSDPSIVLLLQCDMQK*

>P1; TnigVILIP3

TnigVILIP3

MGKHNSKLAPEVLDDLTKSTEFNEAELKQWYKGFLKDCPSGILNLEEFQQLYVKFFPYGDATKFAQHAFRTFDKN

GDGTIDFREFICALSITSRGSFEQKLNWAFNMYDLDGDGKITRMEMLEIIEAIYKMVGTVIMMRMNEDGLTPQQR

VDKIFSKMDKDHNDEITLEEFKEAAKSDPSIVLLLQCDMQK*

>P1; TgutVILIP3

TgutVILIP3

MGKHSSKLAPEMLDDLVRSTEFSEQELKQWYKGFLKDCPTGILNLEEFQQLYIKFFPYGDASKFAQHAFRTFDKN

GDGTIDFREFICALSVTSRGTFEQKLNWAFEMYDLDGDGKITRLEMLEIIEAIYKMVGTVIMMRMNQDGLTPQQR

VDKIFTKMDKDKDDQISLEEFKEAAKSDPSIVLLLQCDMQK*

>P1; GgorVILIP3

GgorVILIP3

MGKTNSKLAPEVLEDLVQNTEFSEQELKQWYKGFLKDCPSGILNLEEFQQLYIKFFPYGDASKFAQHAFRTFDKN

GDGTIDFREFICALSVTSRGSFEQKLNWAFEMYDLDGDGRITRLEMLEIIEAIYKMVGTVIMMRMNQDGLTPQQR

VDKIFKKMDQDKDDQITLEEFKEAAKSDPSIVLLLQC*

>P1; FcatVILIP3

FcatVILIP3

MGKTNSKLAPEVLEDLVQNTEFSEQELKQWYKGFLKDCPSGILNLEEFQQLYIKFFPYGDASKFAQHAFRTFDKN

GDGTIDFREFICALSVTSRGSFEQKLNWAFEMYDLDGDGRITRLEMLEIIEAIYKMVGTVIMMRMNQDGLTPQQR

VDKIFKKMDQDKDDQITLEEFKEAAKSDPSIVLLLQCDMQK*

>P1; CporVILIP3

CporVILIP3

MGKTNSKLAPEVLEDLVQNTEFSEQELKQWYKGFLKDCPSGILNLEEFQQLYIKFFPYGDASKFAQHAFRTFDKN

GDGTIDFREFICALSVTSRGSFEQKLNWAFEMYDLDGDGRITRLEMLEIIEAIYKMVGTVIMMRMNQDGLTPQQR

VDKIFKKMDQDKDDQITLEEFKEAAKSDPSIVLLLQCDMQK*

>P1; AcarVILIP3

AcarVILIP3

MGKHNSKLAPEMLDDLVRSTEFNEQELKQWYKGFLKDCPTGILNLEEFQQLYIKFFPYGDASKFAQHAFRTFDKN

GDGTIDFREFICALSVTSRGSFEQKLNWAFEMYDLDGDGKITRLEMLEIIEAIYKMVGTVIMMRMNQDGLTPQQR

VDKIFAKMDKDKDDQISLEEFKEAAKSDPSIVLLLQCDMQK*

>P1; DordVILIP3

DordVILIP3

MGKTNSKLAPEVLEDLVKSTEFSEQELKQWYKGFLKDCPSGILNLEEFQQLYIKFFPYGDASKFAQHAFRTFDKN

GDGTIDFREFICALSVTSRGSFEQKLNWAFEMYDLDGDGRITRLEMLEIIEAIYKMVGTVIMMRMNQDGLTPQQR

VDKIFKKMDQDKDDQITLEEFKEAAKSDPSIVLLLQCDMQK*

>P1; OpriVILIP3

OpriVILIP3

MGKTNSKLAPEVLEDLVQNTEFSEQELKQWYKGFLKDCPSGILNLEEFQQLYIKFFPYGDASKFAQHAFRTFDKN

GDGTIDFREFICALSVTSRGSFEQKLNWAFEMYDLDGDGRITRLEMLEIIEAIYKMVGTVIMMRMNQDGLTPQQR

VDKIFKKMDQDKDDQITLEEFKEAAKSDPSIVLLLQCDMQK*

>P1; PcapVILIP3

PcapVILIP3

MGKTNSKLAPEVLEDLVQNTEFSEQELKQWYKGFLKDCPSGILNLEEFQQLYIKFFPYGDASKFAQHAFRTFDKN

GDGTIDFREFICALSVTSRGSFEQKLNWAFEMYDLDGDGRITRLEMLEIIEAIYKMVGTVIMMRMNQDGLTPQQR

VDKIFKKMDQDKDDQITLEEFKEAAKSDPSIVLLLQCDMQK*

>P1; DrerVILIP3

DrerVILIP3

MGKHNSKLAPEVLDDLTKSTEFNEAELKQWYKGFLKDCPSGILNLEEFQQLYVKFFPYGDASKFAQHAFRTFDKN

GDGTIDFREFICALSITSRGSFEQKLNWAFNMYDLDGDGKITRMEMLEIIEAIYKMVGTVIMMRMNEDGLTPQQR

VDKIFSKMDKDHNDEISLEEFKEAAKSDPSIVLLLQCDMQK*

>P1; HsapVILIP3

HsapVILIP3

MGKTNSKLAPEVLEDLVQNTEFSEQELKQWYKGFLKDCPSGILNLEEFQQLYIKFFPYGDASKFAQHAFRTFDKN

GDGTIDFREFICALSVTSRGSFEQKLNWAFEMYDLDGDGRITRLEMLEIIEAIYKMVGTVIMMRMNQDGLTPQQR

VDKIFKKMDQDKDDQITLEEFKEAAKSDPSIVLLLQCDMQK*

>P1; MmusVILIP3

MmusVILIP3

MGKNNSKLAPEVLEDLVQNTEFSEQELKQWYKGFLKDCPSGILNLEEFQQLYIKFFPYGDASKFAQHAFRTFDKN

GDGTIDFREFICALSVTSRGSFEQKLNWAFEMYDLDGDGRITRLEMLEIIEAIYKMVGTVIMMRMNQDGLTPQQR

VDKIFKKMDQDKDDQITLEEFKEAAKSDPSIVLLLQCDMQK*

>P1; TrubVILIP3

TrubVILIP3

MGKHNSKLAPEVLDDLTKSTEFNEAELKQWYKGFLKDCPSGILNLEEFQQLYVKFFPYGDATKFAQHAFRTFDKN

GDGTIDFREFICALSITSRGSFEQKLNWAFNMYDLDGDGKITRMEMLEIIEAIYKMVGTVIMMRMNEDGLTPQQR

VDKIFSKMDKDHNDEISLEEFKEAAKSDPSIVLLLQCDMQK*

>P1; GacuVILIP3

GacuVILIP3

MGKHNSKLAPEVLEDLTKSTEFNEAELKQWYKGFLKDCPSGILNLDEFQQLYVKFFPYGDASKFAQHAFRTFDKN

GDGTIDFREFICALSITSRGSFEQKLNWAFNMYDLDGDGKITRVEMLEIIEAIYNMVRTVIMMRMNKDELTPQRV

YKIFSKMDKDHNDEISLEEFKEAAKSDPSIVLLLQCDMQK*

>P1; PpygVILIP3

PpygVILIP3

MGKTNSKLAPEVLEDLVQNTEFSEQELKQWYKGFLKDCPSGILNLEEFQQLYIKFFPYGDASKFAQHAFRTFDKN

GDGTIDFREFICALSVTSRGSFEQKLNWAFEMYDLDGDGRITRLEMLEIIEAIYKMVGTVIMMRMNQDGLTPQQR

VDKIFKKMDQDKDDQITLEEFKEAAKSDPSIVLLLQCDMQK*

>P1; OlatVILIP3

OlatVILIP3

MGKHNSKLAPEVLDDLTKNTEFNEAELKQWYKGFLKDCPSGILNLEEFQQLYVKFFPYGDASKFAQHAFRTFDKN

GDGTIDFREFICALSITSRGSFEQKLNWAFNMYDLDGDGKITRMEMLEIIEAIYKMVGTVIMMRMNEDGLTPQQR

VDKIFSKMDKDHNDEISLEEFKEAAKSDPSIVLLLQCDMQK*

>P1; DordVHIPPOCALCIN

DordVHIPPOCALCIN

MGKQNSKLRPEMLQDLRENTEFSELELQEWYKGFLKDCPTGILNVDEFKKIYANFFPYGDASKFAEHVFRTFDTN

SDGTIDFREFIIALSVTSRGRLEQKLMWAFSMYDLDGNGYISREEMLEIVQAIYKMVSSVMKMPEDESTPEKRTE

KIFRQMDTNNDGKLSLEEFIRGAKSDPSIVRLLQCDPSSASQF*

>P1; MdomVHIPPOCALCIN

MdomVHIPPOCALCIN

MGKQNSKLRPEMLQDLRENTEFSDLELQEWYKGFLKDCPTGILNVEEFKKIYANFFPYGDASKFAEHVFRTFDTN

GDGTIDFREFIIALSVTSRGKLEQKLMWAFSMYDLDGNGYISREEMLEIVQAIYKMVSSVMKMPEDESTPEKRTE

KIFRQMDTNNDGKLSLEEFIRGAKSDPSIVRLLQCDPSSTSQF*

>P1; PcapVHIPPOCALCIN

PcapVHIPPOCALCIN

MGKQNSKLRPEMLQDLRENTEFSELELQEWYKGFLKDCPTGILNVDEFKKIYANFFPYGDASKFAEHVFRTFDTN

SDGTIDFREFIIALSVTSRGRLEQKLMWAFSMYDLDGNGYISREEMLEIVQAIYKMVSSVMKMPEDESTPEKRTE

KIFRQMDTNNDGKLSLEEFIRGAKSDPSIVRLLQCDPSSASQF*

>P1; MlucVHIPPOCALCIN

MlucVHIPPOCALCIN

MGKQNSKLRPEMLQDLRENTEFSELELQEWYKGFLKDCPTGILNVDEFKKIYANFFPYGDASKFAEHVFRTFDTN

SDGTIDFREFIIALSVTSRGRLEQKLMWAFSMYDLDGNGYISREEMLEIVQAIYKMVSSVMKMPEDESTPEKRTE

KIFRQMDTNNDGKLSLEEFIRGAKSDPSIVRLLQCDPSSASQF*

>P1; TtruVHIPPOCALCIN

TtruVHIPPOCALCIN

MGKQNSKLRPEMLQDLRENTEFSELELQEWYKGFLKDCPTGILNVDEFKKIYANFFPYGDASKFAEHVFRTFDTN

SDGTIDFREFIIALSVTSRGRLEQKLMWAFSMYDLDGNGYISREEMLEIVQAIYKMVSSVMKMPEDESTPEKRTE

KIFRQMDTNNDGKLSLEEFIRGAKSDPSIVRLLQCDPSSASQF*

>P1; GgorVHIPPOCALCIN

GgorVHIPPOCALCIN

MGKQNSKLRPEMLQDLRENTEFSELELQEWYKGFLKDCPTGILNVDEFKKIYANFFPYGDASKFAEHVFRTFDTN

SDGTIDFREFIIALSVTSRGRLEQKLMWAFSMYDLDGNGYISREEMLEIVQAIYKMVSSVMKMPEDESTPEKRTE

KIFRQMDTNNDGKLSLEEFIRGAKSDPSIVRLLQCDPSSASQF*

>P1; CporVHIPPOCALCIN

CporVHIPPOCALCIN

MGKQNSKLRPEMLQDLRENTEFSELELQEWYKGFLKDCPTGILNVDEFKKIYANFFPYGDASKFAEHVFRTFDTN

SDGTIDFREFIIALSVTSRGRLEQKLMWAFSMYDLDGNGYISREEMLEIVQAIYKMVSSVMKMPEDESTPEKRTE

KIFRQMDTNNDGKLSLEEFIRGAKSDPSIVRLLQCDPSSASQF*

>P1; DrerVHIPPOCALCIN

DrerVHIPPOCALCIN

MGKQNSKLRPEMLQDLRENTEFTDHELQEWYKGFLKDCPSGHLNVEEFKKIYANFFPYGDASKFAEHVFRTFDTN

NDGTIDFREFIIALSVTSRGKLEQKLKWAFSMYDLDGNGYISREEMLEIVQAIYKMVSSVMKMPEDESTPEKRTD

KIFRQMDLNNDGKLSLEEFIKGAKSDPSIVRLLQCDPSSASQF*

>P1; GgalVHIPPOCALCIN

GgalVHIPPOCALCIN

MGKQNSKLRPEMLQDLRENTEFSDLELQGWYKGFLKDCPSGILNVEEFKKIYANFFPYGDASKFAEHVFRTFDTN

GDGTIDFREFIIALSVTSRGKLEQKLMWAFSMYDLDGNGYISREEMLEIVQAIYKMVSSVMNMPEDESTPEKRTE

KIFRQMDTNNDGKLSLEEFIKGAKSDPSIVRLLQCDPSGAGQL*

>P1; OpriVHIPPOCALCIN

OpriVHIPPOCALCIN

MGKQNSKLRPEMLQDLRENTEFSELELQEWYKGFLKDCPTGILNVDEFKKIYANFFPYGDASKFAEHVFRTFDTN

SDGTIDFREFIIALSVTSRGRLEQKLMWAFSMYDLDGNGYISREEMLEIVQAIYKMVSSVMKMPEDESTPEKRTE

KIFRQMDTNNDGKLSLEEFIRGAKSDPSIVRLLQCDPSSASQF*

>P1; TrubVHIPPOCALCIN

TrubVHIPPOCALCIN

MGKQNSKLRPEMLQDLRENTEFSDHELQEWYKGFLKDCPSGTLNVEEFKKIYANFFPYGDASKFAEHVFRTFDTN

GDGTIDFREFIIALSVTSRGKLEQKLKWAFSMYDLDGNGYISREEMLEIVQAIYKMVSSVMKMPEDESTPEKRTD

KIFRQMDLNNDGKLSLEEFIKGAKSDPSIVRLLQCDPSSA*

>P1; TgutVHIPPOCALCIN

TgutVHIPPOCALCIN

MGKQNSKLRPEMLQDLRENTEFSDLELQGWYKGFLKDCPSGMLDVEEFKKIYANFFPYGDASKFAEHVFRTFDTN

GDGTIDFREFIIALSVTSRGKLEQKLMWAFSMYDLDGNGYISREEMLEIVQAIYKMVSSVMNMPEDESTPEKRTD

KIFRQMDTNNDGKLSLEEFIRGAKSDPSIVRLLQCDPSGAMQ*

>P1; HsapVHIPPOCALCIN

HsapVHIPPOCALCIN

MGKQNSKLRPEMLQDLRENTEFSELELQEWYKGFLKDCPTGILNVDEFKKIYANFFPYGDASKFAEHVFRTFDTN

SDGTIDFREFIIALSVTSRGRLEQKLMWAFSMYDLDGNGYISREEMLEIVQAIYKMVSSVMKMPEDESTPEKRTE

KIFRQMDTNNDGKLSLEEFIRGAKSDPSIVRLLQCDPSSASQF*

>P1; PtroVHIPPOCALCIN

PtroVHIPPOCALCIN

MGKQNSKLRPEMLQDLRENTEFSELELQEWYKGFLKDCPTGILNVDEFKKIYANFFPYGDASKFAEHVFRTFDTN

SDGTIDFREFIIALSVTSRGRLEQKLMWAFSMYDLDGNGYISREEMLEIVQAIYKMVSSVMKMPEDESTPEKRTE

KIFRQMDTNNDGKLSLEEFIRGAKSDPSIVRLLQCDPSSASQF*

>P1; OlatVHIPPOCALCIN

OlatVHIPPOCALCIN

MGKQNSKLRPEMLQDLRENTEFSDHELQEWYKGFLKDCPSGTLNVEEFKKIYANFFPYGDASKFAEHVFRTFDTN

GDGTIDFREFIIALSVTSRGKLEQKLKWAFSMYDLDGNGYISREEMLEIVQAIYKMVSSVMKMPEDESTPEKRTD

KIFRQMDLNNDGKLSLEEFIKGAKSDPSIVRLLQCDPSSASQF*

>P1; MmurVHIPPOCALCIN

MmurVHIPPOCALCIN

MGKQNSKLRPEMLQDLRENTEFSELELQEWYKGFLKDCPTGILNVDEFKKIYANFFPYGDASKFAEHVFRTFDTN

SDGTIDFREFIIALSVTSRGRLEQKLMWAFSMYDLDGNGYISREEMLEIVQAIYKMVSSVMKMPEDESTPEKRTE

KIFRQMDTNNDGKLSLEEFIRGAKSDPSIVRLLQCDPSSASQF*

>P1; RnorVHIPPOCALCIN

RnorVHIPPOCALCIN

MGKQNSKLRPEMLQDLRENTEFSELELQEWYKGFLKDCPTGILNVDEFKKIYANFFPYGDASKFAEHVFRTFDTN

SDGTIDFREFIIALSVTSRGRLEQKLMWAFSMYDLDGNGYISREEMLEIVQAIYKMVSSVMKMPEDESTPEKRTE

KIFRQMDTNNDGKLSLEEFIRGAKSDPSIVRLLQCDPSSASQF*

>P1; CfamVHIPPOCALCIN

CfamVHIPPOCALCIN

MGKQNSKLRPEMLQDLRENTEFSELELQEWYKGFLKDCPTGILNVDEFKKIYANFFPYGDASKFAEHVFRTFDTN

SDGTIDFREFIIALSVTSRGRLEQKLMWAFSMYDLDGNGYISREEMLEIVQAIYKMVSSVMKMPEDESTPEKRTE

KIFRQMDTNNDGKLSLEEFIRGAKSDPSIVRLLQCDPSSASQH*

>P1; GacuVHIPPOCALCIN

GacuVHIPPOCALCIN

MGKQNSKLRPEMLQDLRENTEFSDYELQEWYKGFLKDCPSGTLNVEEFKKIYANFFPYGDASKFAEHVFRTFDTN

ADGTIDFREFIIALSVTSRGKLEQKLKWAFSMYDLDGNGYISRDEMLEIVQAIYKMVSSVMKMPEDESTPEKRTD

KIFRQMDLNNDGKLSLEEFIKGAKSDPSIVRLLQCDPSSASQF*

>P1; SaraVHIPPOCALCIN

SaraVHIPPOCALCIN

MGKQNSKLRPEMLQDLRENTEFSELELQEWYKGFLKDCPTGILNVDEFKKIYANFFPYGDASKFAEHVFRTFDTN

SDGTIDFREFIIALSVTSRGRLEQKLMWAFSMYDLDGNGYISREEMLEIVQAIYKMVSSVMKMPEDESTPEKRTE

KIFRQMDTNNDGKLSLEEFIRGAKSDPSIVRLLQCDPSSASQF*

>P1; XtroVHIPPOCALCIN

XtroVHIPPOCALCIN

MGKQNSKLRPEMLQDLRENTEFSDHELQEWYKGFLKDCPSGILNVEEFKKIYANFFPYGDASKFAEHVFRTFDTN

GDGTIDFREFIIALSVTSRGKLEQKLKWAFSMYDLDGNGYISREEMLEIVQAIYKMVSSVMKMPEDESTPEKRTE

KIFRQMDTNNDGKLSLEEFIKGAKSDPSIVRLLQCDPSTASQF*

>P1; BtauVHIPPOCALCIN

BtauVHIPPOCALCIN

MGKQNSKLRPEMLQDLRENTEFSELELQEWYKGFLKDCPTGILNVDEFKKIYANFFPYGDASKFAEHVFRTFDTN

SDGTIDFREFIIALSVTSRGRLEQKLMWAFSMYDLDGNGYISREEMLEIVQAIYKMVSSVMKMPEDESTPEKRTE

KIFRQMDTNNDGKLSLEEFIRGAKSDPSIVRLLQCDPSSASQF*

>P1; PvamVHIPPOCALCIN

PvamVHIPPOCALCIN

MGKQNSKLRPEMLQDLRENTEFSELELQEWYKGFLKDCPTGILNVDEFKKIYANFFPYGDASKFAEHVFRTFDTN

SDGTIDFREFIIALSVTSRGRLEQKLMWAFSMYDLDGNGYISREEMLEIVQAIYKMVSSVMKMPEDESTPEKRTE

KIFRQMDTNNDGKLSLEEFIRGAKSDPSIVRLLQCDPSSASQF*

>P1; EtelVHIPPOCALCIN

EtelVHIPPOCALCIN

MGKQNSKLRPEMLQDLRENTEFSELELQEWYKGFLKDCPTGILNVDEFKKIYANFFPYGDASKFAEHVFRTFDTN

SDGTIDFREFIIALSVTSRGRLEQKLMWAFSMYDLDGNGYISREEMLEIVQAIYKMVSSVMKMPEDESTPEKRTE

KIFRQMDTNNDGKLSLEEFIRGAKSDPSIVRLLQCDPSSASQF*

>P1; MdomRECOVERIN

MdomRECOVERIN

MGNSKSGSLSKEILEELQLNTKYKEEELCAWYQSFLKECPSGRITKKEFESIYSKFFPDADPKAYAQHVFRSFDA

NSDGTLDFKEYIIALHMTTAGKTNQKLEWAFSLYDIDGNGAISKNEVLEIIMAIFKMINPEDLKHLPEDENTPEK

RAEKIWSFFGKKEDDKLTEKEFIEGTLANKEILRLIQYEPQKVKEKLKDKKP*

>P1; OgaRECOVERIN

OgaRECOVERIN

MGNSKSGALSKEILEELQLNTKFTEEELCAWYQSFLKECPSGRITQQEFQSIYAKFFPDADPKAYAQHVFRSFDA

NSDGTLDFKEYVIALHMTTAGKPNQKLEWAFSLYDVDGNGAISKNEVLDIVTAIFKMINPEDVKHLPDDENTPEK

RAEKIWNFFGKKEDDKLTEEEFIEGTLANKEILRLIQFEPQKVKERLKEKKP*

>P1; EeurRECOVERIN

EeurRECOVERIN

MGNSKSGALSKEILEELQLNTRFTQEELCAWYQSFLKECPTGRITQQEFAGIYAKFFPDSDPKAYAQHVFRSFDA

NSDGTLDFKEYVVALHMTSAGKTTQKLEWAFSLYDVDGNGTISKNEVLEIVMAIFKMINPEDLKHLPDDENTPEK

RAEKIWAFFGKKEDDKLTEEEFIEGTLANKEILRLIQFEPQKVKERIKEKKP*

>P1; OcunRECOVERIN

OcunRECOVERIN

MGNSKSGALSKEILEDLQLNTKFTEEELCTWYQSFLKECPSGRITRQEFESIYAKFFPDADPKAYAQHVFRSFDA

NSDGTLDFKEYVIALHMTTAGKPSQKLEWAFSLYDVDGNGAISKNEVLEIVMAIFKMINPEDVKHLPDDENTPEK

RTEKIWGFFGKKDDDKLTEEEFIEGTMANKEILRLIQFEPQKVKERIKEKKQ*

>P1; MmulRECOVERIN

MmulRECOVERIN

MGNSKSGALSKEILEELQLNTKFSEEELSSWYQSFLKDCPSGRITQQQFQSIYAKFFPDTDPKAYAQHVFRSFDS

NLDGTLDFKEYVIALHMTTAGKTNQKLEWAFSLYDVDGNGTISKNEVLEIVMAIFKMITPEDVKLLPEDENTPEK

RAEKIWKYFGKSDDDKLTEKEFIEGTLANKEILRLIQFEPQKVKEKIKNA*

>P1; PcapRECOVERIN

PcapRECOVERIN

MGNSRSGALSKEILEELQLNTKFTEEELCTWYQSFLKECPSGRITRQEFESIYSKFFPDADPKAYAQHVFRSFDA

NNDGTLDFKEYVIALHMTTSGKTNQKLEWAFSLYDVDGNGSISKSEVLDIIVAIFKMISPEDVKHLPEDENTPEK

RTEKIWRFFGKKDDDKLTEKEFIDGTLANKEILRLIQFEPQKVKERLKEKKT*

>P1; PpygRECOVERIN

PpygRECOVERIN

MGNSKSGALSKEILEELQLNTKFSEEELCSWYQSFLKDCPTGRITQQQFQSIYAKFFPDTDPKAYAQHVFRSFDS

NLDGTLDFKEYVIALHMTTAGKTNQKLEWAFSLYDVDGNGTISKNEVLEIVMAIFKMITPEDVKLLPDDENTPEK

RAEKIWKYFGKNDDDKLTEKEFIEGTLANKEILRLIQFEPQKVKEKMKNA*

>P1; DordRECOVERIN

DordRECOVERIN

MGNSKSGALSKEILEELQLNTKFTEEELSAWYQSFLKECPSGRITRQEFQSIYAKFFPDADPKAYAQHVFRSFDA

NSDGTLDFKEYVIALHMTTAGKPTQKLEWAFSLYDVDGNGAISKNEVLEIVMAIFKMIPEDVKHPDDENTPEKRA

EKIWTFFGKKDDDKLTEEEFIEGTLANKEILRLIQFEPQKVKERIKEKKP*

>P1; SaraRECOVERIN

SaraRECOVERIN

MGNSKSGALSKEILEELQLNTKFTQEELCAWYQSFLKECPTGRITQQEFASIYAKFFPDADPKAYAQHVFRSFDA

NSDGTLDFKEYVVALHMTTVGKTSQKLEWAFSLYDVDGNGAISKGEVLEIVMAIFKMISPEDVKNLPDDENTPEK

RAEKIWAFFGKKDNDKLTEEEFIDGTMANKEILRLIQFEPQKVKERVKEKKP*

>P1; TrubRECOVERIN

TrubRECOVERIN

MGNTKSSALSKELLEDLKSHTKYGEAELCSWYQSFLKECPGGKISKEQFEGIYASFFPNADPSQYARHVFRSFDT

NADGTLDFKEYIVALHLTSGGKTLQKLEWAFALYDVDGNGTISKNEILEIVRSIFNMIPADDQKTLPEDENTPEK

RAEKVWEFFGKKDNDKISEGEFIQGVMDNKEILRLIQYDEPQKIKDKLKEKKQ*

>P1; DrerRECOVERIN

DrerRECOVERIN

MGNTKSGALSKELLEDLKLNTKYTEEELCAWYTSFLKECPSGRITKEQFEGIYASFFPDADPTAYARHVFRSFDT

NADGTLDFKEYIVALHLTSSGKTLRKLEWAFALYDVDGNGTISKNEVQEIVRSIFNMVPVEDQKNLPDDENTPEK

RADKIWAFFGKQDNDKIGEGEFIQGVMENKDILRLIQYDEPKKIQEKLKEKKH*

>P1; OlatRECOVERIN

OlatRECOVERIN

MGNTKSSALSKELLEELKSNTKYSEAELCTWYQSFLKECPSGKITKEQFEGIYASFFPGADPSAYARHVFRSFDT

NADGTLDFKEYIVALHLTSGGKTLQKLEWAFALYDVDGNGTISKNEIQEIVRSIFNMIPADDQKNLPDDENTPEK

RAEKIWAFFGKKENDKISEGEFIQGVMDNKDILRLIQYDEPQKIKDKLKEKKH*

>P1; StriRECOVERIN

StriRECOVERIN

MGNSKSGALSKEILEDLQLNTKFTEEELCAWYQSFLKECPSGRISRKEFESIYGKFFPDADPKAYAQHVFRSFDT

NSDGTLDFKEYVVALHMTTGGKPTQKLEWAFSLYDVDGNGTISKSEVLEIVMAIFKMISPEDVKLLPDDENTPEK

RAEKIWAYFGKKDDDKLTEQEFIEGTMANKDILRLIQFEPQKVKEKIKEKKP*

>P1; GacuRECOVERIN

GacuRECOVERIN

MGNTKSSALSKELLEELKSNTKYSESELCTWYQSFLKECPGGKISKQQFEGIYASFFPDADPTKYARHVFRSFDT

NADGTLDFKEYIVALHLTSGGKTLQKLEWAFALYDVDGNGTISKSEILEIVRSIFNMIPADDQKNLPEDENTPEK

RAEKVWEFFGKKDNDKISEGEFIQGVMENKDILRLIQYDEPQKIKDKLKEKKQ*

>P1; PtroRECOVERIN

PtroRECOVERIN

MGNSKSGALSKEILEELQLNTKFSEEELCSWYQSFLKDCPTGRITQQQFQSIYAKFFPDTDPKAYAQHVFRSFDS

NLDGTLDFKEYVIALHMTTAGKTNQKLEWAFSLYDVDGNGTISKNEVLEIVMAIFKMITPEDVKLLPDDENTPEK

RAEKIWKYFGKNDDDKLTEKEFIEGTLANKEILRLIQFEPQKVKEKMKNA*

>P1; TsyrRECOVERIN

TsyrRECOVERIN

MGNSRSGALSKEILEELQLNTKFSEEELCTWYQTFLKECPSGRITQQQFQGIYAKFFPDADPKAYAQHVFRSFDA

NSDGTLDFKEYVIALHMTSAGKTTQKLEWAFSLYDVDGNGTSKNEVLEIVMAIFKMIKPEDVKYLADDENTPEKR

AEKIWKFFGKKDDDKLTEKEFIEGTLGNTEILRLIQFEPQKVKDRLKEKKP*

>P1; MmusRECOVERIN

MmusRECOVERIN

MGNSKSGALSKEILEELQLNTKFTEEELSAWYQSFLKECPSGRITRQEFESIYSKFFPDSDPKAYAQHVFRSFDA

NSDGTLDFKEYVIALHMTTAGKPTQKLEWAFSLYDVDGNGTISKNEVLEIVMAIFKMIKPEDVKLLPDDENTPEK

RAEKIWAFFGKKEDDKLTEEEFIEGTLANKEILRLIQFEPQKVKERIKEKKQ*

>P1; TnigRECOVERIN

TnigRECOVERIN

MGNSRSSALSKELLEDLKSNTKYSEAELCCWYQSFLKECPGGKISKEQFEGIYASFFPDADPSQYARHVFRSFDT

NADGTLDFKEYMVALHLTSGGKTLQKLEWAFALYDVDGNGTISKNEILEIVRSIFNMIPADDQKNLPEDENTPEK

RAEKVWEFFGKKENDKISEGEFIQGVMDNKEILRLIQYDEPQKIKDKLKEKKQ*

>P1; EcabRECOVERIN

EcabRECOVERIN

MGNSKSGALSKEILEELQLNTKFTEEELCAWYQSFLKECPSGLITRQEFQSIYSKFFPEADPKAYAQHVFRSFDA

NSDGTLDFKEYVIALHMTTAGKTNQKLEWAFSLYDVDGNGTISKNEVLEIIMAIFKMINPEDLKNLPDDENTPEK

RAEKIWGFFGKKDDDKLTEKEFIEGTLANKEILRLIQFEPQKVKEKIKEKKH*

>P1; LafrRECOVERIN

LafrRECOVERIN

MGNSKSGALSKEILEELQLNTKFTEEELCTWYQSFLKECPSGRITRQEFESIYSKFFPDADPKAYAQHVFRSFDA

NSDGTLDFKEYVIALHMTTAGKTNQKLEWAFSLYDVDGNGSISKNEVLEIIMAIFKMINPEDMKHLPEDENTPEK

RAEKIWGFFGKKDDDKLTEEEFIEGTLANKEILRLIQFEPQKVKERLKEKKP*

>P1; BtauRECOVERIN

BtauRECOVERIN

MGNSKSGALSKEILEELQLNTKFTEEELSSWYQSFLKECPSGRITRQEFQTIYSKFFPEADPKAYAQHVFRSFDA

NSDGTLDFKEYVIALHMTRAGKTNQKLEWAFSLYDVDGNGTISKNEVLEXVQAIFKMISPEDTKHLPEDENTPEK

RAEKIWGFFGKKDDDKLTEKEFIEGTLANKEILRLIQFEPQKVKEKLKEKKL*

>P1; RnorRECOVERIN

RnorRECOVERIN

MGNSKSGALSKEILEELQLNTKFTEEELSAWYQSFLKECPSGRITRQEFESIYSKFFPDSDPKAYAQHVFRSFDA

NSDGTLDFKEYVIALHMTTAGKPTQKLEWAFSLYDVDGNGTISKNEVLEIVMAIFKMIKPEDVKNLPDDENTPEK

RAEKIWAFFGKKDDDKLTEEEFIEGTLANKEILRLIQFEPQKVKERIKEKKQ*

>P1; CporRECOVERIN

CporRECOVERIN

MGNSRSGALSKEILEELQGNTKFTEEELATWYQSFLKECPNGRISRQQFESIYSKFFPDSDPKAYAQHVFRSFDA

NNDGTLDFKEYVIALHMTTAGKTNQKLEWAFSLYDVDGNGAISKNEVLEIVMAIFKMINPEDVKHLPDDENTPEK

RAEKIWTFFGKKDDDKLSEEEFIEGTLANKEILRLIQFEPQKVKERIK*

>P1; HsapRECOVERIN

HsapRECOVERIN

MGNSKSGALSKEILEELQLNTKFSEEELCSWYQSFLKDCPTGRITQQQFQSIYAKFFPDTDPKAYAQHVFRSFDS

NLDGTLDFKEYVIALHMTTAGKTNQKLEWAFSLYDVDGNGTISKNEVLEIVMAIFKMITPEDVKLLPDDENTPEK

RAEKIWKYFGKNDDDKLTEKEFIEGTLANKEILRLIQFEPQKVKEKMKNA*

>P1; GgorRECOVERIN

GgorRECOVERIN

MGNSKSGALSKEILEELQLNTKFSEEELCSWYQSFLKDCPTGRITQQQFQSIYAKFFPDTDPKAYAQHVFRSFDS

NLDGTLDFKEYVIALHMTTAGKTNQKLEWAFSLYDVDGNGTISKNEVLEIVMAIFKMITPEDVKLLPDDENTPEK

RAEKIWKYFGKNDDDKLTEKEFIEGTLANKEILRLIQFEPQKVKEKMKNA*

>P1; MmurRECOVERIN

MmurRECOVERIN

MGNSKSGALSKEILDELQLNTKFTEEELCAWYQSFLKECPSGRITQQEFQSIYAKFFPDSDPKAYAQHVFRSFDA

NSDGTLDFKEYVIALHMTTAGKTTQKLEWAFSLYDVDGNGAISKNEVLEIVMAIFKMINPEDVKHLPDDENTPEK

RAEKIWKFFGKNDDDKLTEDEFIEGTLANKEILRLIQFEPQKVKEKLKEKKP*

>P1; MlucRECOVERIN

MlucRECOVERIN

MGNSRSGALSQEILEELQLSTKFTQEELCAWYQSFLKECPGGRITRQQFQGIYAKFFPDADPKAYAEHVFRSFDA

NSDGTLDFKEYVVALHMTTAGRPSQKLEWAFSLYDVDGNGAISKSEVLEIVMAIFKMINPEDAKHLPDDENTPEK

RADKIWGFFGKKDDDKLTEKEFIEGTLANKEILRLIQLEPQKVKERLKEKEKNP*

>P1; XtroRECOVERIN

XtroRECOVERIN

MGNSKSSALSKEILEELQLNTKFSQEELCTWYQSFLKECPTGRISKQQFEGIYSKFFPDADPKAYARHVFRSFDS

NNDGTLDFKEYMIALHMTSSGKANQKLEWAFSLYDVDGNGTINKSEVLEIITAIFKMINTEDQKHLPEDENTPER

RTNKIWDFFGKKDNDKLTEGEFIQGIMNNKEILRLIQFEPQKVKEK*

>P1; CfamRECOVERIN

CfamRECOVERIN

MGNSKSGALSKEILEELQLNTKFTEEELCSWYQSFLKECPSGRITKQEFQSIYSKFFPEADPKAYAQHVFRSFDA

NSDGTLDFKEYVIALHMTSAGKTNQKLEWAFSLYDVDGNGAISKSEVLEIVMAIFKMISPEDVKQLPEDENTPEK

RAEKIWGFFGKKDDDKLTEEEFIEGTLANKEILRLIQFEPRKVKEKLKEKKH*

>P1; DrerKChIP1

DrerKChIP1

DKVDDELEMTMVCHRPEGLEQLEAQTNFSKQELQVLYRGFKNECPSGVVNEDTFKHIYAQFFPHGDASTYAHYLF

HAFDTRNNGSIKFEDFVMGLSTLLRGTVRDKLEWTFHLYDINKDGFINKEEMTEIVRAIYDMMGKYTYPALKGDV

PKAHVDAFFEKMDKNKDGVVTLEEFVLACQEDENMMRSMQLFENVM*

>P1; MmusKChIP1

MmusKChIP1

DKIEDELEMTMVCHRPEGLEQLEAQTNFTKRELQVLYRGFKNECPSGVVNEETFKQIYAQFFPHGDASTYAHYLF

NAFDTTQTGSVKFEDFVTALSILLRGTVHEKLRWTFNLYDINKDGYINKEEMMDIVKAIYDMMGKYTYPVLKEDT

PRQHVDVFFQKMDKNKDGIVTLDEFLESCQEDDNIMRSLQLFQNVM*

>P1; TgutKChIP1

TgutKChIP1

DKIEDELEMTTVCHRPEGLEQLEAQTNFTKRELQVLYRGFKNECPSGVVNEETFKQIYAQFFPHGDASMYAHYLF

NAFDTAQNGSVKFEDFVMALSILLRGTVHEKLRWTFNLYDINKDGCINKEEMMDIVKAIYDMMGKYTYPVLKEDA

PRQHVEVFFQKMDKNKDGVVTLDEFIESCQEDDNIMRSLQLFENVM*

>P1; TrubKChIP1

TrubKChIP1

DKADDELEMTTVCHRPEALDQLEAQTNFSKQELQILYRGFKNECPSGVVNEETFKHIYAQFFPHGYASMYAHYLF

NAFDTANNGSIKFKDFVTGMSILLRGTVREKLEWTFHLYDINRDGYINREEMTEIVRAIYDMMGKYTYPALKGDV

PQQHVDAFFQKMDKNKDGVVTLEEFVIACQEDETMMRSMQLFENVM*

>P1; CfamKChIP1

CfamKChIP1

DKIEDELEMTMVCHRPEGLEQLEAQTNFTKRELQVLYRGFKNVRPTGGGKEETFKQNHAQVFPHGDASMYAHYLF

HAFDTTQTGSVKFEDFVTALSILLRGTVHEKLRWTFNLYDINKDGYINKEEMMDIVKAIYDMMGKYTYPVLKEDT

PRQHVDVFFQKMDKNKDGIVTLDEFLESCQEDDNIMRSLQLFQNVM*

>P1; EcabKChIP1

EcabKChIP1

DKIEDELEMTMVCHRPEGLEQLEAQTNFTKRELQVLYRGFKNECPSGVVNEETFKQIYAQFFPHGDASTYAHYLF

HAFDTTQTGSVKFEDFVTALSILLRGTVHEKLRWTFNLYDINKDGYINKEEMMDIVKAIYDMMGKYTYPVLKEDT

PRQHVDVFFQKMDKNKDGIVTLDEFLESCQEDDNIMRSLQLFQNVM*

>P1; EtelKChIP1

EtelKChIP1

DKIEDELEMTVVCHRPEGLEQLEAQTNFTKRELQVLYRGFKNECPSGVVNEETFKQIYAQFFPHGDASTYAHYLF

NAFDTTQTGSVKFEDFVTALSILLRGTIHEKLRWTFNLYDINKDGYINKEEMMDIVKAIYDMMGKYTYPVLKEDT

PRQHVDIFQKMDKNKDGIVTLDEFLESCQEDDNIIRSLQLFQNVM*

>P1; OgarKChIP1

OgarKChIP1

KIEDELEMTMVCHRPEGLEQLEAQTNFTKRELQVLYRGFKNECPSGVVNEETFKQIYAQFFPHGDASTYAHYLFN

AFDTTQTGSVKFEDFVTALSILLRGTVHEKLRWTFNLYDINKDGYINKEEMIDIVKAIYDMMGKYTYPVLREDTP

RQHVDVFFQKMDKNKDGIVTLDEFLESCQEDDNIMRSLQLFQNVM*

>P1; TnigKChIP1

TnigKChIP1

DKVDDELEMTTVCHRPEGLEQLEAQTNFSKQELQILYRGFKNECPSGVVNEETFKHIYAQFFPHGASMYAHYLFN

AFDTSNNGSIKFKDFVTGLSILLRGTVREKLEWTFHLYDINRDGYINREEMTEIVRAIYDMMGKYTYPALKGDVP

QQHVDAFFQKMDKNKDGVVTLEEFIIACQEDETMMRSMQLFENVM*

>P1; GgalKChIP1

GgalKChIP1

DKIEDELEMTTVCHRPEGLEQLEAQTNFTKRELQVLYRGFKNECPSGVVNEETFKQIYAQFFPHGDASMYAHYLF

NAFDTAQYGSVKFEDFVMALSILLRGTVHEKLRWTFNLYDINKDGYINKEEMMDIVKAIYDMMGKYTYPVLKEDA

PRQHVEVFFQKMDKNKDGVVTLDEFIESCQEDDNIMRSLQLFENVM*

>P1; BtauKChIP1

BtauKChIP1

GKAGAELEMTMVCHRPEGLEQLEAQTNFTKRELQVLYRGFKNECPSGVVNEETFKQIYAQFFPHGDASMYAHYLF

HAFDTTQTGSVKFEDFVTALSILLRGTVHEKLRWTFNLYDINKDGYINKEEMMDIVKAIYDMMGKYTYPVLKEDT

PRQHVDIFFQKMDKNKDGIVTLDEFLESCQEDDNIMRSLQLFQNVM*

>P1; MmulKChIP1

MmulKChIP1

DKIEDELEMTMVCHRPEGLEQLEAQTNFTKRELQVLYRGFKNECPSGVVNEDTFKQIYAQFFPHGDASTYAHYLF

NAFDTTQTGSVKFEDFVTALSILLRGTVHEKLRWTFNLYDINKDGYINKEEMMDIVKAIYDMMGKYTYPVLKEDT

PRQHVDVFFQKMDKNKDGIVTLDEFLESCQEDDNIMRSLQLFQNVM*

>P1; XtroKChIP1

XtroKChIP1

DKVEDELEMTTVCYRPEGLEQLEAQTNFNKRELQVLYRGFKNECPSGVVNEDTFKLIYSQFFPHGDASMYAHYLF

NAFDAAQSGSVKFEDFVAALSVLLRGSIHEKLRWTFNLYDINKDGNINKEEMMDIVKAIYDMMGKYTYPVLKEDA

PKQHVEVFFQKMDKNKDGVVTLDEFIESCQEDDNIMRSLQLFENVM*

>P1; AcarKChIP1

AcarKChIP1

DKIEDELEMTTVCYRPEGLEQLEAQTNFTKKELQVLYRGFKNECPSGAVNEETFKQIYAQFFPHGDASMYAHYLF

NAFDTAQNGSVKFEDFVTALSILLRGTVHEKLRWTFNLYDINKDGYINKEEMMDIVKAIYDMMGKYTYPVLKDDA

PRQHVEVFFQKMDKNKDGVVTLDEFIESCQEDDNIMRSLQLFENVM*

>P1; HsapKChIP1

HsapKChIP1

DKIEDELEMTMVCHRPEGLEQLEAQTNFTKRELQVLYRGFKNECPSGVVNEDTFKQIYAQFFPHGDASTYAHYLF

NAFDTTQTGSVKFEDFVTALSILLRGTVHEKLRWTFNLYDINKDGYINKEEMMDIVKAIYDMMGKYTYPVLKEDT

PRQHVDVFFQKMDKNKDGIVTLDEFLESCQEDDNIMRSLQLFQNVM*

>P1; RnorKChIP1

RnorKChIP1

DKIEDDLEMTMVCHRPEGLEQLEAQTNFTKRELQVLYRGFKNECPSGVVNEETFKQIYAQFFPHGDASTYAHYLF

NAFDTTQTGSVKFEDFVTALSILLRGTVHEKLRWTFNLYDINKDGYINKEEMMDIVKAIYDMMGKYTYPVLKEDT

PRQHVDVFFQKMDKNKDGIVTLDEFLESCQEDDNIMRSLQLFQNVM*

>P1; MdomKChIP1

MdomKChIP1

DKIEDELEMTMVCHRPEGLEQLEAQTNFSKRELQVLYRGFKNECPSGVVNEETFKQIYSQFFPHGDASMYAHYLF

NAFDTTQTGSVKFEDFVTALSILLRGTVHEKLRWTFNLYDINKDGYINKEEMIDIVKAIYDMMGKYTYPVLKDDT

PRQHVDVFFQKMDKNKDGVVTLDEFLESCQEDDNIMRSLQLFENVM*

>P1; ChofKChIP1

ChofKChIP1

DKIEDELEMTMVCHRPEGLEQLEAQTNFTKRELQVLYRGFKNECPSGVVNEETFKQIYAQFFPHGDASTYAHYLF

NAFDTTQTGSVKFEDFVTALSILLRGTVHEKLRWTFNLYDINKDGYINKEEMMDIVKAIYDMMGKYTYPVLKEDT

PRQHVDVFFQKMDKNKDGIVTLDEFLESCQEDDNIMRSLQLFQNVM*

>P1; PcapKChIP1

PcapKChIP1

DKIEDELEMTIVCHRPEGLEQLEAQTNFTKRELQVLYRGFKNECPSGVVNEETFKQIYAQFFPHGDASTYAHYLF

NAFDTTQTGSVKFEDFVTALSVLLRGTVHEKLRWTFNLYDINKDGYINKEEMMDIVKAIYDMMGKYTYPVLKEDT

PRQHVDVFFQKMDKNKDGIVTLDEFLESCQEDDNIMRSLQLFQNVM*

>P1; OlatKChIP1

OlatKChIP1

DKTEDELEMTTVCHRPEGLDQLEAQTNFTKQELQILYRGFKNECPSGVVNEETFKHIYAQFFPHGDASTYAHYLF

NAFDTTNNGSIKFKDFVMGLSTLLRGTLREKLEWTFHLYDINRDGYINREEMTEIVRAIYDMMGKYTYPAIKGDV

PQQHVDAFFQQKMDKNKDGVVTLEEFVVACQEDETMMRSMQLFENVM*

>P1; PpygKChIP1

PpygKChIP1

DKIEDELEMTMVCHRPEGLEQLEAQTNFTKRELQVLYRGFKNECPSGVVNEDTFKQIYAQFFPHGDASTYAHYLF

NAFDTTQTGSVKFEDFVTALSILLRGTVHEKLRWTFNLYDINKDGYINKEEMMDIVKAIYDMMGKYTYPVLKEDT

PRQHVDVFFQKMDKNKDGIVTLDEFLESCQEDDNIMRSLQLFQNVM*

>P1; PtroKChIP1

PtroKChIP1

DKIEDELEMTMVCHRPEGLEQLEAQTNFTKRELQVLYRGFKNECPSGVVNEDTFKQIYAQFFPHGDASTYAHYLF

NAFDTTQTGSVKFEDFVTALSILLRGTVHEKLRWTFNLYDINKDGYINKEEMMDIVKAIYDMMGKYTYPVLKEDT

PRQHVDVFFQKMDKNKDGIVTLDEFLESCQEDDNIMRSLQLFQNVM*

>P1; TsyrKChIP1

TsyrKChIP1

DKIEDELEMTMVCHRPEGLEQLEAQTNFTKRELQVLYRGFKNECPSGVVNEETFKQIYAQFFPHGDASTYAHYLF

NAFDTTQTGSVKFEDFVTALSILLRGTVHEKLRWTFNLYDINKDGYINKEEMMDIVKAIYDMMGKYTYPVLKEDT

PRQHVDVFFQKMDKNKDGIVTLDEFLESCQEDDNIMRSLQLFQNVI*

>P1; GacuKChIP1

GacuKChIP1

DKIDDELEMTTVCYRPEGLDQLEAQTNFSKQELQILYRGFKNECPSGVVDEDTFKHIYAQFFPHGDASMYAHYLF

NAFDTTNNGSIKFKDFVVGLSTLLRGSMREKLEWTFYLYDINRDGYINREEMTEIVRAIYDMMGKYTYPALKGDV

PQQHVDAFFQKMDKNKDGVVTLEEFIMACQEDETMMRSMQLFENVM*

>P1; OanaKChIP1

OanaKChIP1

DKIEDELEMAMVCHRPEGLEQLEAQTNFTKRELQVLYRGFKNECPSGVVNEETFKQIYAQFFPHGDASMYAHYLF

NAFDTAQTGSVKFEDFVTALSILLRGTVLEKLRWTFNLYDINKDGYINKEEMMDIVKAIYDMMGKYTYPVLKDDA

PRQHVDVFFQKMDKNRDGVVTLDEFIESCQEDDNIMKSLQLFENVM*

>P1; CporKChIP1

CporKChIP1

DKIEDELEMTMVCHRPEGLEQLEAQTNFTKRELQVLYRGFKNECPSGAVNEETFKQIYAQFFPHGDASTYAHYLF

NAFDTTQTGSVKFEDFVTALSILLRGTVHEKLRWTFNLYDINKDGYINKEEMMDIVKAIYDMMGKYTYPVLKEDT

PRQHVDVFFQKMDKNKDGIVTLDEFLESCQEDDNIMRSLQLFQNVM*

>P1; PvamKChIP1

PvamKChIP1

DKIEDELEMTMVCHRPEGLEQLEAQTNFTKSELQVLYRGFKNECPSGVVNEETFKQIYAQFFPHGDASTYAHYLF

HAFDTTQTGSVKFEDFVTALSILLRGTVHEKLRWTFNLYDINKDGYINKEEMMDIVKAIYDMMGKYTYPVLKEDT

PRQHVDVFFQKMDKNKDGIVTLDEFLESCQEDDNIMRSLQLFQNVM*

>P1; CporKChIP2

CporKChIP2

SVEDEFELCTVCHRPEGLEQLQEQTKFTRKELQVLYRGFKNECPSGMVNEENFKQIYSQFFPQGDSSTYATFLFN

AFDTNHDGCVSFEDFVAGLSVILRGTIDDRLNWAFNLYDLNKDGCITKEEMLDIMKSIYDMMGKYTYPALREEAP

REHVESFFQKMDRNKDGVVTIEEFIESCQKDENIMRSMQLFDNVI*

>P1; EcabKChIP2

EcabKChIP2

SVEDEFELSTVCHRPEGLEQLQEQTKFTRKELQVLYRGFKNECPSGIVNEENFKQIYSQFFPQGDSSTYATFLFN

AFDTNHDGSVSFEDFVAGLSVILRGTIDDRLNWAFNLYDLNKDGCITKEEMLDIMKSIYDMMGKYTYPALREEAP

REHVESFFQKMDRNKDGVVTIEEFIESCQKDENIMRSMKLFDNVI*

>P1; GgalKChIP2

GgalKChIP2

GSMQPAHLKAAAGAPSGGDGHFAWTKAPATRQAKLKKALKQECPSGIVNEENFKQIYSQFFPQGDSSTYATFLFN

AFDTDHDGSVSFEDFVSGLSIILRGTIDDRLNWAFNLYDLNKDGCITKEEMLDIMKSIYDMMGKYTYPAMREEAP

REHVENFFQKMDRNKDGVVTIEEFLESCQKDENIMRSMQQCRQRD*

>P1; MmusKChIP2

MmusKChIP2

SVEDEFELSTVCHRPEGLEQLQEQTKFTRRELQVLYRGFKNECPSGIVNEENFKQIYSQFFPQGDSSNYATFLFN

AFDTNHDGSVSFEDFVAGLSVILRGTIDDRLNWAFNLYDLNKDGCITKEEMLDIMKSIYDMMGKYTYPALREEAP

REHVESFFQKMDRNKDGVVTIEEFIESCQQDENIMRSMQLFDNVI*

>P1; BtauKChIP2

BtauKChIP2

SVEDEFELSTVCHRPEGLEQLQEQTKFTRKELQVLYRGFKNECPSGIVNEENFKQIYSQFFPQGDSSTYATFLFN

AFDTNHDGSVSFEDFVAGLSVILRGTTDDRLNWAFNLYDLNKDGCITKEEMLDIMKSIYDMMGKYTYPALREEAP

REHVESFFQKMDRNKDGVVTIEEFIESCQKDENIMRSMQLFDNVI*

>P1; AcarKChIP2

AcarKChIP2

SIEDEFELSTVCHRPEGLDQLQQQSKFTRKELQVLYRGFKNECPSGIVNEDSFKQIYSQFFPQGDSSTYATFLFN

AFDTDHDGSVSFEDFVAGLSIILRGTIDDRLNWAFNLYDLNKDGCITKEEMLDIMKSIYDMMGKYTYPAMRDEAP

QEHVENFFQKMDRNKDGVVTIEEFIESCQKDENIMNSMKFFDNVI*

>P1; HsapKChIP2

HsapKChIP2

SVDDEFELSTVCHRPEGLEQLQEQTKFTRKELQVLYRGFKNECPSGIVNEENFKQIYSQFFPQGDSSTYATFLFN

AFDTNHDGSVSFEDFVAGLSVILRGTVDDRLNWAFNLYDLNKDGCITKEEMLDIMKSIYDMMGKYTYPALREEAP

REHVESFFQKMDRNKDGVVTIEEFIESCQKDENIMRSMQLFDNVI*

>P1; TbelKChIP2

TbelKChIP2

SVEDEFELSTVCHRPEGLEQLQEQTKFTRKELQVLYRGFKNECPSGIVNEENFKQIYSQFFPQGDSSTYATFLFN

AFDTNHDGSVSFEDFVAGLSVILRGTVDDRLNWAFNLYDLNKDGCITKEEMLDIMKSIYDMMGKYTYPALREEAP

REHVESFFQKMDRNKDGVVTIEEFIESCQKDENIMRSMQLFDNVI*

>P1; OlatKChIP2

OlatKChIP2

SIEDDFELSTVCHRPESMDKLEELTKFTKKELQVLYRGFKNECPSGVVNEDNFKTIYSQFFPQGDSSMYAHFLFE

AFDTNKNGSVSFEDFVFGLSIILRGTINDRLNWAFNLYDLNKDGCITKEEMLDIMKSIYDMMGKCTYPTMQEDAP

LEHVESFFQKMDQNKDGVVTIEEFIESCKKDENIMQSMQLFDNVI*

>P1; EeurKChIP2

EeurKChIP2

SVEDEFELSTVCHRPEGLEQLQEQTKFTRKELQVLYRGFKNECPSGIVNEENFKQIYSQFFPQGDSSTYATSLAL

TQHDSDSEDFVAGLSVILRGTIDDRLNWAFNLYDLNKDGCITKEEMLDIMKSIYDMMGKYTYPALREEAPREHVE

SFFQKMDRNKDGVVTIEEFIESCQKDENIMRSMRLFDNVI*

>P1; TgutKChIP2

TgutKChIP2

SVEDEFELSTVCHRPEGLEQLQEQTKFTRKELQVLYRGFKNECPSGIVNEENFKQIYSQFFPQGDSSTYATFLFN

AFDTDHDGSVSFEDFVSGLSTILRGTIDDRLNWAFNLYDLNKDGCITKEEMLDIMKSIYDMMGKYTYPAMREEAP

REHVENFFQKMDRNKDGVVTIEEFLESCQKDENIMRSMQLFDSVI*

>P1; TnigKChIP2

TnigKChIP2

SVEDDFELSTVCHRPESMDKLQEQTKFTKKELQVLYRGFKNECPSGVVNEENFKTIYSQFFPQGDSSMYAHFLFE

AFDTNKNGSVSFETRTLCLALSIILRGTVNDRLNWFNLYDLNKDGCIFTFQEMLDIMKSIYDMMGKYTYPTMQDD

APREHVENFFQKMDRNKDGVVTIEEFIESCKKDENIMQSMQLFDNVI*

>P1; GgorKChIP2

GgorKChIP2

SVDDEFELSTVCHRPEGLEQLQEQTKFTRKELQVLYRGFKNECPSGIVNEENFKQIYSQFFPQGDSSTYATFLFN

AFDTNHDGSVSFEDFVAGLSVILRGTVDDRLNWAFNLYDLNKDGCITKEEMLDIMKSIYDMMGKYTYPALREEAP

REHVESFFQKMDRNKDGVVTIEEFIESCQKDENIMRSMQLFDNVI*

>P1; FcatKChIP2

FcatKChIP2

SVEDEFELSTVCHRPEGLEQLQEQTKFTRKELQVLYRGFKNECPSGIVNEENFKQIYSQFFPQGDSSTYATFLFN

AFDTNHDGSVSFEDFVAGLSVILRGTIDDRLNWAFNLYDLNKDGCITKEEMLDIMKSIYDMMGKYTYPALREEAP

REHVESFFQKMDRNKDGVVTIEEFIESCQKDENIMRSMQLFDNVI*

>P1; MdomKChIP2

MdomKChIP2

SVEDEFELSTVCHRPEGLEQLQEQTKFTRKELQVLYRGFKNECPSGIVNEENFKQIYSQFFPQGDSSTYATFLFN

AFDTNHDGSVSFEDFVAGLSVILRGTIDDRLNWAFNLYDLNKDGCITKEEMLDIMKSIYDMMGKYTYPALREEAP

REHVENFFQKMDRNKDGVVTIEEFIESCQKDENIMRSMQLFDDVI*

>P1; MmulKChIP2

MmulKChIP2

SVEDEFELSTVCHRPEGLEQLQEQTKFTRKELQVLYRGFKNECPSGIVNEENFKQIYSQFFPQGDSSTYATFLFN

AFDTNHDGSVSFEDFVAGLSVILRGTVDDRLNWAFNLYDLNKDGCITKEEMLDIMKSIYDMMGKYTYPALREEAP

REHVENFFQKMDRNKDGVVTIEEFIESCQKDENIMRSMQLFDNVI*

>P1; CfamKChIP2

CfamKChIP2

SVEDEFELSTVCHRPEGLEQLQEQTKFTRKELQVLYRGFKNECPSGIVNEENFKQIYSQFFPQGDSSTYATFLFN

AFDTNHDGSVSFEDFVAGLSVILRGTIDDRLNWAFNLYDLNKDGCITKEEMLDIMKSIYDMMGKYTYPALREEAP

REHVESFFQKMDRNKDGVTIEEFIESCQKDENIMRSMQLFDNVI*

>P1; RnorKChIP2

RnorKChIP2

SVEDEFELSTVCHRPEGLEQLQEQTKFTRRELQVLYRGFKNECPSGIVNEENFKQIYSQFFPQGDSSNYATFLFN

AFDTNHDGSVSFEDFVAGLSVILRGTIDDRLSWAFNLYDLNKDGCITKEEMLDIMKSIYDMMGKYTYPALREEAP

REHVESFFQKMDRNKDGVVTIEEFIESCQQDENIMRSMQLFDNVI*

>P1; PvamKChIP2

PvamKChIP2

SVEDEFELSTVCHRPEGLEQLQEQTKFTRKELQVLYRGFKNECPSGIVNEENFKQIYSQFFPQGDSSTYATFLFN

AFDTNHDGSVSFEDFVAGLSVILRGTIDDRLNWAFNLYDLNKDGCITKEEMLDIMKSIYDMMGKYTYPALREEAP

REHVETFFQKMDRNKDGVVTIEEFIESCQKDENIMRSMQLFDNVI*

>P1; GacuKChIP2

GacuKChIP2

SVEDDFELSTVCHRPESMDKLEEQTKFTKKELQVLYRGFKNECPCGVVNEENFKTIYSQFFPQGDSSMYAHFLFE

AFDTNKNGSVSFEDFVFGLSIILRGTINDRLNWAFNLYDLNKDGCITKEEMFDIMKSIYDMMGKYTYPTMQDDIP

REHVESFFQKMDRNKDGVVTIEEFIESCKKDENIMQSMQLFDNVI*

>P1; PtroKChIP2

PtroKChIP2

NVDDEFELSTVCHRPEGLEQLQEQTKFTRKELQVLYRGFKNECPSGIVNEENFKQIYSQFFPQGDSSTYATFLFN

AFDTNHDGSVSFEDFVAGLSVILRGTVDDRLNWAFNLYDLNKDGCITKEEMLDIMKSIYDMMGKYTYPALREEAP

REHVESFFQKMDRNKDGVVTIEEFIESCQKDENIMRSMQLFDNVI*

>P1; DordKChIP2

DordKChIP2

SVEDEFELSTVCHRPEGLEQLQEQTKFTRKELQVLYRGFKNECPSGIVNEENFKQIYSQFFPQGDSSTYATFLFN

AFDTNHDGSVSFEDFVAGLSVILRGTIDDRLNWAFNLYDLNKDGCITKEEMLDIMKSIYDMMGKYTYPALREEAP

REHVETFFQKMDRNKDGVVTIEEFIESCQKDENIMRSMQLFDNVI*

>P1; TtruKChIP2

TtruKChIP2

SVEDEFELSTVCHRPEGLEQLQEQTKFTRKELQVLYRGFKNECPSGIVNEENFKQIYSQFFPQGDSSTYATFLFN

AFDTNHDGSVSFEDFVAGLSVILRGTTDDRLNWAFNLYDLNKDGCITKEEMLDIMKSIYDMMGKYTYPALREEAP

REHVESFFQKMDRNKDGVVTIEEFIESCQKDENIMRSMQLFDNVI*

>P1; MmurKChIP2

MmurKChIP2

SVEDEFELSTVCHRPEGLEQLQEQTKFTRKELQVLYRGFKNECPSGIVSEENFKQIYSQFFPQGDSSTYATFLFN

AFDTNHDGSVSFEDFVAGLSVILRGTVDDRLNWAFNLYDLNKDGCITKEEMLDIMKSIYDMMGKYTYPALREEAP

REHVENFFQKMDRNKDGVVTIEEFIESCQKDENIMKSMQLFDNVI*

>P1; LafrKChIP2

LafrKChIP2

SVEDEFELSTVCHRPEGLEQLQEQTKFTRKELQVLYRGFKNECPSGIVNEENFKQIYSQFFPQGDSSTYATFLFN

AFDTNHDGSVSFEDFVAGLSVILRGTIDDRLNWAFNLYDLNKDGCITKEEMLDIMKSIYDMMGKYTYPALREEAP

REHVENFFQKMDRNKDGVVTIEEFIESCQKDENIMRSMQLFDNVI*

>P1; PpygKChIP2

PpygKChIP2

SVEDEFELSTVCHRPEGLEQLQEQTKFTRKELQVLYRGFKNECPSGVNEENFKQIYSQFFPQGDSSTYATFLFNA

FDTNHDGSVSFEDFVAGLSVILRGTVDDRLNWAFNLYDLNKDGCITKEEMLDIMKSIYDMMGKYTYPALREEAPR

EHVESFFQKMDRNKDGVVTIEEFMESCQKDENIMRSMQLFDNVI*

>P1; XtroKChIP2

XtroKChIP2

SVEDDFELSTVCHRPEGLEQLQEQTKFTKKELQVLYRGFKNECPSGIVNEENFKQIYSQFFPQGDSSMYAHFLFN

AFDTDHSGSVSFEDFVAGLSVILRGTIDDKLNWAFNLYDLNKDGCITKEEMLDIMKSIYDMMGKYTYPNMREEAP

REHVENFFQKMDRNKDGVVTIEEFIESCQKDENIMRSMQLFDNVI*

>P1; OgarKChIP2

OgarKChIP2

SVEDEFELSTVCHRPEGLEQLQEQTKFTRKELQVLYRGFKNECPSGIVSEENFKQIYSQFFPQGDSSTYATFLFN

AFDTNHDGSVSFEDFVAGLSVILRGTIDDRLNWAFNLYDLNKDGCITKEEMLDIMKSIYDMMGKYTYPALREEAP

REHVENFFQKMDRNKDGVVTIEEFIESCQKDENIMRSMQLFDNVI*

>P1; _Bos_taurus_Frq

_Bos_taurus_Frq

MGKSNSKLKPEVVEELTRKTYFTEKEVQQWYKGFIKDCPSGQLDAAGFQKIYKQFFPFGDPTKFATFVFNVFDEN

KDGRIEFSEFIQALSVTSRGTLDEKLRWAFKLYDLDNDGYITRNEMLDIVDAIYQMVGNTVELPEEENTPEKRVD

RIFAMMDKNADGKLTLQEFQEGTKADPSIVQALSLYDGLV*

>P1; _Gallus_gallus_Frq

_Gallus_gallus_Frq

MGKSNSKLKPEVVEELTRKTYFTEKEVQQWYKGFIKDCPSGQLDAAGFQKIYKQFFPFGDPTKFATFVFNVFDEN

KDGRIEFSEFIQALSVTSRGTLDEKLRWAFKLYDLDNDGYITRNEMLDIVDAIYQMVGNTVELPEEENTPEKRVD

RIFAMMDKNADGKLTLQEFQEGSKADPSIVQALSLYDGLV*

>P1; Homo_sapiens_Frq

Homo_sapiens_Frq

MGKSNSKLKPEVVEELTRKTYFTEKEVQQWYKGFIKDCPSGQLDAAGFQKIYKQFFPFGDPTKFATFVFNVFDEN

KDGRIEFSEFIQALSVTSRGTLDEKLRWAFKLYDLDNDGYITRNEMLDIVDAIYQMVGNTVELPEEENTPEKRVD

RIFAMMDKNADGKLTLQEFQEGSKADPSIVQALSLYDGLV*

>P1; Pongo_pygmaeus_Frq

Pongo_pygmaeus_Frq

MGKSNSKLKPEVVEELTRKTYFTEKEVQQWYKGFIKDCPSGQLDAAGFQKIYKQFFPFGDPTKFATFVFNVFDEN

KDGRIGFSEFIQALSVTSRGTLDEKLRWAFKLYDLDNDGYITRNEMLDIVDAIYQMVGNTVELPEEENTPEKRVD

RIFAMMDKNADGKLTLQEFQEGSKADPSIVQALSLYDGLV*

>P1; Ratus_norvegicus_Frq

Ratus_norvegicus_Frq

MGKSNSKLKPEVVEELTRKTYFTEKEVQQWYKGFIKDCPSGQLDAAGFQKIYKQFFPFGDPTKFATFVFNVFDEN

KDGRIEFSEFIQALSVTSRGTLDEKLRWAFKLYDLDNDGYITRNEMLDIVDAIYQMVGNTVELPEEENTPEKRVD

RIFAMMDKNADGKLTLQEFQEGSKADPSIVQALSLYDGLV*

>P1; Mus_musculus_Frq

Mus_musculus_Frq

MGKSNSKLKPEVVEELTRKTYFTEKEVQQWYKGFIKDCPSGQLDAAGFQKIYKQFFPFGDPTKFATFVFNVFDEN

KDGRIEFSEFIQALSVTSRGTLDEKLRWAFKLYDLDNDGYITRNEMLDIVDAIYQMVGNTVELPEEENTPEKRVD

RIFAMMDKNADGKLTLQEFQEGSKADPSIVQALSLYDGLV*

>P1; Monodelphis_domestica_Frq

Monodelphis_domestica_Frq

MGKSNSKLKPEVVEELTRKTYFTEKEVQQWYKGFIKDCPSGQLDAAGFQKIYKQFFPFGDPTKFATFVFNVFDEN

KDGRIEFSEFIQALSVTSRGTLDEKLRWAFKLYDLDNDGYITRNEMLDIVDAIYQMVGNTVELPEEENTPEKRVD

RIFAMMDKNSDGKLTLQEFQEGSKADPSIVQALSLYDGLV*

>P1; Xenopus_tropicalis_Frq

Xenopus_tropicalis_Frq

MGKSNSKLKPEVVEELTRKTYFTEKEVQQWYKGFIKDCPSGQLDAAGFQKIYKQFFPFGDPTKFATFVFNVFDEN

KDGRIEFSEFIQALSVTSRGTLDEKLRWAFKLYDLDNDGYITRNEMLDIVDAIYQMVGNTVELPEEENTPEKRVD

RIFAMMDKNSDGKLTLQEFQEGSKADPSIVQALSLYDGLV*

>P1; _Danio_rerio_Frq1

_Danio_rerio_Frq1

MGKSNSKLKPEVVEELTRKTYFTEKEVQQWYKGFIKDCPSGQLDAAGFQKIYKQFFPFGDPTKFASFVFNVFDEN

KDGRIEFSEFIQALSVTSRGTLDEKLRWAFKLYDLDNDGYITRDEMLNIVDAIYQMVGNTVDLPEEENTPEKRVD

RIFAMMDKNADGKLTLQEFQEGSKADPSIVQALSLYDGLV*

>P1; Danio_rerio_Frq2

Danio_rerio_Frq2

MGKSNSKLKPEVVEDLCRKTYFTEKEVQQWYKGFIKDCPSGQLDSSGFQKIYKQFFPFGDPTKFATFVFNVFDEN

KDGRIEFSEFIQALSVTSRGTLDEKLRWAFKLYDLDNDGYITRDEMLNIVDAIYQMVGNTVELPEEENTPEKRVD

RIFAMMDKNADGMLTLQEFQEGSKADPSIVQALSLYDGLV*

>P1; Oryzias_latipes_Frq

Oryzias_latipes_Frq

MGKSNSKLKPEVVEELTRKTYFTEKEVQQWYKGFIKDCPSGQLDAVGFQKIYKQFFPFGDPTKFASFVFNVFDEN

KDGRIEFSEFIQALSVTSRGTLDEKLRWAFKLYDLDNDGYITRDEMLNIVDAIYQMVGNTVELPEEENTPEKRVD

RIFAMMDKNADGKLTLQEFQEGSKADPSIVQALSLYDGLV*

>P1; Gasterosteus_aculeatus_Frq

Gasterosteus_aculeatus_Frq

MGKSNSKLKPEVVEELTRKTYFTEKEVQQWYKGFIKDCPSGQLDAVGFQKIYKQFFPFGDPTKFASFVFNVFDEN

KDGRIEFSEFIQALSVTSRGTLDEKLRWAFKLYDLDNDGYITRDEMLNIVDAIYQMVGNSVELPEEENTPEKRVD

RIFAMMDKNADGKLTLQEFQEGSKADPSIVQALSLYDGLV*

>P1; Takifugu_rubripes_Frq

Takifugu_rubripes_Frq

MGKSNSKLKPEVVEELTRKTYFTEKEVQQWYKGFIKDCPSGQLDAAGFQKIYKQFFPFGDPTKFASFVFNVFDEN

KDGRIEFSEFIQALSVTSRGTLDEKLRWAFKLYDLDNDGYITRDEMLNIVDAIYQMVGNTVELPEEENTPEKRVD

RIFAMMDKNADGKLTLQEFQEGSKADPSIVQALSLYDGLV*

>P1; Tetraodon_nigroviridis_Frq

Tetraodon_nigroviridis_Frq

MGKSNSKLKPEVVEELTSKTYFTEKEVQQWYKGFIKDCPSGQLDAAGFQKIYKQFFPFGDPTKFASFVFNVFDEN

KDGRIEFSEFIQALSVTSRGTLDEKLRWAFKLYDLDNDGYITRDEMLNIVDAIYQMVGNTVELPEEENTPEKRVD

RIFAMMDKNADGKLTLQEFQEGSKADPSIVQALSLYDGLV*

>P1; Procavia_capensis_Frq

Procavia_capensis_Frq

MGKSNSKLKPEVVEELTRKTYFTEKEVQQWYKGFIKDCPSGQLDAAGFQKIYKQFFPFGDPTKFATFVFNVFDEN

KDGRIEFSEFIQALSVTSRGTLDEKLRWAFKLYDLDNDGYITRNEMLDIVDAIYQMVGNTVELPEEENTPEKRVD

RIFAMMDKNADGKLTLQEFQEGSKADPSIVQALSLYDGLV*

>P1; Taeniopygia_guttata_Frq

Taeniopygia_guttata_Frq

MGKSNSKLKPEVVEELTRKTYFTEKEVQQWYKGFIKDCPSGQLDAAGFQKIYKQFFPFGDPTKFATFVFNVFDEN

KDGRIEFSEFIQALSVTSRGTLDEKLRWAFKLYDLDNDGYITRNEMLDIVDAIYQMVGNTVELPEEENTPEKRVD

RIFAMMDKNADGKLTLQEFQEGSKADPSIVQALSLYDGLV*

>P1; Canis_familiaris_Frq

Canis_familiaris_Frq

MGKSNSKLKPEVVEELTRKTYFTEKEVQQWYKGFIKDCPSGQLDAAGFQKIYKQFFPFGDPTKFATFVFNVFDEN

KDGRIEFSEFIQALSVTSRGTLDEKLRWAFKLYDLDNDGYITRNEMLDIVDAIYQMVGNTVELPEEENTPEKRVD

RIFAMMDKNADGKLTLQEFQEGSKADPSIVQALSLYDGLV*

>P1; Perca_flavescens_Frq

Perca_flavescens_Frq

MGKSNSKLKPEVVEELTRKTYFTEKEVQQWYKGFIKDCPSGQLDAVGFQKIYKQFFPFGDPTKFASFVFNVFDEN

KDGRIEFSEFIQALSVTSRGTLDEKLRWAFKLYDLDNDGYITRDEMLNIVDAIYQMVGNTVELPEEENTPEKRVD

RIFAMMDKNADGKLTLQEFQEGSKADPSIVQALSLYDGLV*

>P1; Pimephales_promelas_Frq

Pimephales_promelas_Frq

MGKSSSKLKPEVVEELCRKTYFTEKEVQQWYKGFIKDCPSGQLDAAGFQKIYKQFFPFGDPTKFATFVFNVFDEN

KDGRIEFSEFIQALSVTSRGTLDEKLRWAFKLYDLDNDGYITRDEMLNIVDAIYQMVGNTVELPEEENTPEKRVD

RIFAMMDKNADGLLTLQEFQEGSKGDPSIVQALSLYDGLV*

>P1; Salmo_salar_Frq

Salmo_salar_Frq

MGKSNSKLKPEMVEELTRKTYFTEKEVQQWYKGFIKDCPSGQLDSAGFQKIYKQFFPFGDPTKFASFVFNVFDEN

KDGRIEFAEFIQALSVTSRGTLDEKLRWAFKLYDLDNDGYITRDEMLNIVDAIYQMVGNTVELPEEENTPEKRVD

RIFAMMDKNADGLLTLKEFQEGSKADPSIVQALSLYDGLV*

>P1; Xenopus_laevis_Frq

Xenopus_laevis_Frq

MGKSNSKLKPEVVEELTRKTYFTEKEVQQWYKGFIKDCPSGQLDATGFQKIYKQFFPFGDPTKFATFVFNVFDEN

KDGRIEFSEFIQALSVTSRGTLDEKLRWAFKLYDLDNDGYITRNEMLDIVDAIYQMVGNTVELPEEENTPEKRVD

RIFAMMDKNSDGKLTLQEFQEGSKADPSIVQALSLYDMGKRNSKLKPDEIEELRTKTYFSEEEIQQWYKGFMKDC

PDGKLTLEGFTKIYRQFFPFGDPSKFAAFVFNVFDENKDGYIEFDEFLQALSVTSRGNVDEKLRWAFRLYDLDSD

GFITREELLDIVDAIYKMVGNMVKLPEEENTPEKRVNKIFEIMDKNKDDRLTFDEFLEGSKKDPTIIQALTLYDG

*

>P1; lymnaea_stagnalis_Frq

lymnaea_stagnalis_Frq

MGKRASKLRPEEVDELKAHTYFTESEIKQWHKGFRKDCPDGKLTLEGFTKIYQQFFPFGDPSKFANFVFNVFDEN

KDGFISFSEFLQALSVTSRGTVEEKLKWAFRLYDLDNDGYITRDELLDIVDAIYRMVGESVTLPEEENTPEKRVN

RIFQVMDKNKDDQLTFEEFLEGSKEDPTIIQAL*

>P1; Ciona_intestinalis_Frq

Ciona_intestinalis_Frq

MGNRKSKLKPEVLEKLTKQTKFTEAELHQWHKGFLHDCPTGKLSYEEFQGIYRQFFPQGDSAKFAKLVFTTFDEN

KDGTVEFDEFIIALSVTSRGSLDEKLHWAFQLYDLDNDGFITKNEMLNIVEAIFAMVGDAVNLPAEENTPQKRVE

KIFNVMDKNKDGKLTKEEFLVGAKSDPSIVQALSIYDGLV*

>P1; Caenorhabditis_elegans_Frq

Caenorhabditis_elegans_Frq

MGKGNSKLKSSQIRDLAEQTYFTEKEIKQWYKGFVRDCPNGMLTEAGFQKIYKQFFPQGDPSDFASFVFKVFDEN

KDGAIEFHEFIRALSITSRGNLDEKLHWAFKLYDLDQDGFITRNEMLSIVDSIYKMVGSSVQLPEEENTPEKRVD

RIFRMMDKNNDAQLTLEEFKEGAKADPSIVHALSLYEGLSS*

>P1; MmulKChIP3

MmulKChIP3

SSDGELELSTVRHQPEGLDQLQAQTKFTKKELQSLYRGFKNECPTGLVDEDTFKLIYAQFFPQGDATTYAHFLFN

AFDADGNGAIHFEDFVVGLSILLRGTVHEKLKWAFNLYDINKDGYITKEEMLAIMKSIYDMMGRHTYPILREDTP

AEHVERFFQKMDRNQDGVVTIEEFLETCQKDENIMSSMQLFENVI*

>P1; RnorKChIP3

RnorKChIP3

SSDSELELSTVRHQPEGLDQLQAQTKFTKKELQSLYRGFKNECPTGLVDEDTFKLIYSQFFPQGDATTYAHFLFN

AFDADGNGAIHFEDFVVGLSILLRGTVHEKLKWAFNLYDINKDGYITKEEMLAIMKSIYDMMGRHTYPILREDAP

LEHVERFFQKMDRNQDGVVTIDEFLETCQKDENIMSSMQLFENVI*

>P1; GacuKChIP3

GacuKChIP3

SCDGEFELSMVRHQPEGLDQLQAQTQFTRKELQSLYRGFKNECPSGLVDEETFKTIYSQFFPQGDATTYAHFLFN

AFDMDRNGSIRFEDFVIGLSVLLRGSVTEKLRWAFNLYDINKDGLITKEEMMSIMTSIYDMMGRYTLPSVRDDSP

YEHVERFFQKMDRNRDGVVTIDEFIETCQKDENIMASMQLFENVI*

>P1; BtauKChIP3

BtauKChIP3

SSDSELELSAVRHQPEGLDQLQAQTKFTKKELQSLYRGFKNECPTGLVDEDTFKLIYSQFFPQGDATTYAHFLFN

AFDADGNGAIRFEDFVVGLSILLRGTVHEKLKWAFNLYDINKDGYITKEEMLAIMKSIYDMMGRHTYPILREDAP

LEHVERFFQKMDRNQDGVVTIDEFLETCQKDENIMSSMQLFENVI*

>P1; PpygKChIP3

PpygKChIP3

SSDSELELSTVRHQPEGLDQLQAQTKFTKKELQSLYRGFKNECPTGLVDEDTFKLIYAQFFPQGDATTYAHFLFN

AFDADGNGAIHFEDFVVGLSILLRGTVHEKLKWAFNLYDINKDGYITKEEMLAIMKSIYDMMGRHTYPILREDAP

AEHVERFFQKMDRNQDGVVTIEEFLETCQKDENIMSSMQLFENVI*

>P1; GgorKChIP3

GgorKChIP3

SSDSELELSTVRHQPEGLDQLQAQTKFTKKELQSLYRGFKNECPTGLVDEDTFKLIYAQFFPQGDATTYAHFLFN

AFDADGNGAIHFEDFVVGLSILLRGTVHEKLKWAFNLYDINKDGYITKEEMLAIMKSIYDMMGRHTYPILREDAP

AEHVERFFEKMDRNQDGVVTIEEFLETCQKDENIMSSMQLFENVI*

>P1; PtroKChIP3

PtroKChIP3

SSDSELELSTVRHQPEGLDQLQAQTKFTKKELQSLYRGFKNECPTGLVDEDTFKLIYAQFFPQGDATTYAHFLFN

AFDADGNGAIHFEDFVVGLSILLRGTVHEKLKWAFNLYDINKDGYITKEEMLAIMKSIYDMMGRHTYPILREDAP

AEHVERFFEKMDRNQDGVVTIEEFLETCQKDENIMSSMQLFENVI*

>P1; MmurKChIP3

MmurKChIP3

SSDSELELSTVRHQPEGLDQLQAQTKFTKKELQSLYRGFKNECPTGLVDEDTFKLIYSQFFPQGDATTYAHFLFN

AFDADGNGAIRFEDFVVGLSILLRGTVHEKLKWAFNLYDINKDGYITKEEMLAIMKSIYDMMGRHTYPILREDAP

MEHVERFFQKMDRNQDGVVTIDEFLETCQKDENIMSSMQLFENVI*

>P1; CporKChIP3

CporKChIP3

SSDSELELSTVRHQPEGLDQLQTQTKFTKKELQSLYRGFKNECPTGLVDEDTFKLIYSQFFPQGDATTYAHFLFN

AFDADGNGAIHFEDFVVGLSILLRGTVHEKLKWAFNLYDINKDGYITKEEMLAIMKSIYDMMGHHTYPVLREDAP

LQHVEKFFQKMDRNQDGVVTIEEFLETCQKDENIMNSMQLFENVI*

>P1; EcabKChIP3

EcabKChIP3

SSDSELELSTVRHQPEGLDQLQAQTKFTKKELQSLYRGFKNNHCPPPPLPVTVFLTLISVDPPNGTAPGWAHPGC

RAAALDVMGFVHLRDFVVGLSILLRGTVHEKLKWAFNLYDINKDGYITKEEMLAIMKSIYDMMGRHTYPILREDA

PLEHVERFFQKMDRNQDGVVTIDEFLETCQKDENIMSSMQLFENVI*

>P1; DrerKChIP3

DrerKChIP3

STDSELELSAVRHQPEGLEQLQAQTQFTRKELQSLYRGFKNECPSGLVDEETFKSIYSQFFPQGDATTYAHFLFN

AFDMDRNGSIRFEDFVIGLSVLLRGSVTEKLRWAFNLYDINKDGYITKEEMLAIMKSIYDMMGRYTSPCVKDDAA

FEHVEKFFQKMDRNRDGVVTLEEFIETCQKDENIMSSMQLFENVI*

>P1; MmusKChIP3

MmusKChIP3

SSDSELELSTVRHQPEGLDQLQAQTKFTKKELQSLYRGFKNECPTGLVDEDTFKLIYSQFFPQGDATTYAHFLFN

AFDADGNGAIHFEDFVVGLSILLRGTVHEKLKWAFNLYDINKDGCITKEEMLAIMKSIYDMMGRHTYPILREDAP

LEHVERFFQKMDRNQDGVVTIDEFLETCQKDENIMNSMQLFENVI*

>P1; TrubKChIP3

TrubKChIP3

TCEGDLELSMVRHQPEGLDQLQAQTQFTRKELQSLYRGFKNECPSGLVDEETFKNIYSQFFPQGDATMYAHFLFN

AFDMDRSGSIRFEDFVIGLSVLLRGSVPEKLRWAFNLYDINKDGYITKEEMMAIMTSIYDMMGRYTLPTIRDDSP

FEHVEKFFQKMDRNRDGMVTVEEFIETCQKDENIMSSMQLFEHVI*

>P1; MdomKChIP3

MdomKChIP3

SSDSELELSAVRHQPEGLDQLQAQTKFTKKELQSLYRGFKNECPTGLVDEETFKLIYSQFFPQGDATTYAHFLFN

AFDADGNGAIHFEDFVVGLSILLRGTVHEKLKWAFNLYDINKDGYITKEEMLAIMKSIYDMMGRHTYPILREEAP

LEHVERFFQKMDRNQDGVVTIEEFLETCQKDENIMSSMQLFENVI*

>P1; OlatKChIP3

OlatKChIP3

TSDSDLELSMVRHQPEGLEQLQAQTKFTKKELQSLYRGFKNECPSGLVDEETFKSIYSQFFPQGDATTYAHFLFN

AFDMDRNGSIRFEDFVIGLSVLLRGSITEKLRWAFNLYDINKDGLVTKEEMLAIMTSIYDMMGRYTLPSVREESP

FEHVEKFFQKMDRNRDGVVTIDEFIETCQKDEDIMASMQLFENVI*

>P1; TnigKChIP3

TnigKChIP3

TCEGDLELSMVRHQPEGLDQLQAQTQFTRKELQSLYRGFKNECPSGLVDEETFKNIYSQFFPQGDATMYAHFLFN

AFDMDRSGSIRFEDFVIGLSVLLRGSVTEKLRWAFNLYDINKDGYITKEEMMAIMTSIYDMMGRYTLPTIRDDSP

FEHVEKFFQKMDRNRDGMVTIDEFIETCQKDENIMSSMQLFENVI*

>P1; OanaKChIP3

OanaKChIP3

SSDGELELSTLRHQPEGLDQLQAQTKFTKKELQSLYRGFKNGCCSGAKSEATEKTIHTPTSPGTSTHTILHFEGN

TFIFEEGGRARMWDFVVGLSVLLRGTVHEKLKWAFNLYDINKDGYITKEEMLAIMKSIYDMMGRHTFPILRDDAP

LEHVEKFFQKMDRNQDGVVTMEEFLETCQKDENIMSSMQLFENVI*

>P1; HsapKChIP3

HsapKChIP3

SSDSELELSTVRHQPEGLDQLQAQTKFTKKELQSLYRGFKNECPTGLVDEDTFKLIYAQFFPQGDATTYAHFLFN

AFDADGNGAIHFEDFVVGLSILLRGTVHEKLKWAFNLYDINKDGYITKEEMLAIMKSIYDMMGRHTYPILREDAP

AEHVERFFEKMDRNQDGVVTIEEFLEACQKDENIMSSMQLFENVI*

>P1; CfamKChIP3

CfamKChIP3

SSDSELELSTVRHQPEGLDQLQAQTKFTKKELQSLYRGFKNECPTGLVDEDTFKLIYSQFFPQGDATTYAHFLFN

AFDADGNGAIHFEDFVVGLSILLRGTVHEKLKWAFNLYDINKDGYITKEEMLAIMKSIYDMMGRHTYPILREDAP

LEHVERFFQKMDRNQDGVVTIDEFLETCQKDENIMSSMQLFENVI*

>P1; MlucKChIP3

MlucKChIP3

TSDSELELSMVRHQPEGLEQLQAQTKFTKMELQSLYRGFKNECPSGLVDEDTFKLIYAQFFPQGDATTYAHFLFN

AFDADGNGAICFEDFVVGLSILLRGTVQEKLKWAFNLYDINKDGYITKEEMLAIMKSIYDMMGRHTYPLLREDAP

LEHVERFFQKMDRNQDGVVTIDEFLETCQKDENIMSSMQLFENVI*

>P1; XtroKChIP3

XtroKChIP3

SSDSDIELSTVRHQPEGLDQLQAVTKFTKKELQSLYRGFKNECPSGLVDEETFKLIYSQFFPQGDATMYAHFLFN

AFDMDRSGAIRFEDFVIGLSILLRGTIHEKLKWAFNLYDINKDGYITKEEMLAIMKSIYDMMGRYTYPLLRDDAP

IEHVERFFQKMDRNRDGVVTIDEFLETCQKDENIMRSMQLFENVI*

>P1; TtruKChIP3

TtruKChIP3

SSDSELELSTVRHQPEGLDQLQAQTKFTKKELQSLYRGFKNECPTGLVDEDTFKLIYSQFFPQGDSTTYAHFLFN

AFDADGNGAIRFEDFVVGLSILLRGTVHEKLKWAFNLYDINKDGYITKEEMLAIMKSIYDMMGRHTYPILREDAP

LEHVERFFQKMDRNQDGVVTIDEFLETCQKDENIMSSMQLFENVI*

>P1; OpriKChIP3

OpriKChIP3

SSDSELELSTVRHQPEGLDQLQAQTKFTKKELQSLYRGFKNECPTGLVDEDTFKLIYSQFFPQGDATTYAHFLFN

AFDADGNGAIRFEDFVVGLSILLRGTVHEKLKWAFNLYDINKDGYITKEEMLAIMKSIYDMMGRHTYPILREDAP

REHVERFFQKMDRNQDGVVTMDEFLETCQKDENIMNSMQLFENVI*

>P1; CfamKChIP4

CfamKChIP4

MATVRHRPEALELLEAQSKFTKKELQILYRGFKNECPSGVVNEETFKEIYSQFFPQGDSTTYAHFLFNAFDTDHN

GAVSFEDFIKGLSILLRGTVQEKLNWAFNLYDINKDGYITKEEMLDIMKAIYDMMGKCTYPVLKEDAPRQHVETF

FQKMDKNKDGVVTIDEFIESCQKDENIMRSMQLFENVI*

>P1; AcarKChIP4

AcarKChIP4

CAVWRHRPEALELLEAQSKFTKEGLQILYRGFKNECPSGVVNEETFKEIYSQFFPQGDSTTYAHFLFNAFDTDHN

GCVSFEDFVMGLSILLRGTVQEKLNWAFNLYDINKDGYVTKEEMLDIMKAIYDMMGKCTYPVVKEDAPRQHVETF

FQKMDKNKDGVVTIDEFIESCQKDENIMRSMQLFENVI*

>P1; MdomKChIP4

MdomKChIP4

MATVRHRPEALELLEAQSKFTKKELPILYRGFKNECPSGVVNEDTFKEIYSQFFPQGDSTTYAHFLFNAFDTDHN

GSVSFEVFVMGLSILLRGTVQEKLNWAFNLYDINKDGYITKEEMLDIMKAIYDMMGKCTYPVLKEDAPRQHVETF

FQKMDKNKDGVVTIDEFIESCQKDENIMRSMQLFENVI*

>P1; RnorKChIP4

RnorKChIP4

MATVRHRPEALELLEAQSKFTKKELQILYRGFKNECPSGVVNEETFKEIYSQFFPQGDSTTYAHFLFNAFDTDHN

GAVSFEDFIKGLSILLRGTVQEKLNWAFNLYDINKDGYITKEEMLDIMKAIYDMMGKCTYPVLKEDAPRQHVETF

FQKMDKNKDGVVTIDEFIESCQKDENIMRSMQLFENVI*

>P1; MmusKChIP4

MmusKChIP4

MATVRHRPEALELLEAQSKFTKKELQILYRGFKNECPSGVVNEETFKEIYSQFFPQGDSTTYAHFLFNAFDTDHN

GAVSFEDFIKGLSILLRGTVQEKLNWAFNLYDINKDGYITKEEMLDIMKAIYDMMGKCTYPVLKEDAPRQHVETF

FQKMDKNKDGVVTIDEFIESCQKDENIMRSMQLFENVI*

>P1; GgalKChIP4

GgalKChIP4

MATVRHRPEALELLEAQSKFTKKELQILYRGFKNECPSGVVNEETFKEIYSQFFPQGDSTTYAHFLFNAFDTDHN

GSVSFEDFVMGLSILLRGTVQEKLNWAFNLYDINKDGYITKEEMLDIMKAIYDMMGKCTYPVLKEDTPRQHVETF

FQKMDKNKDGVVTIDEFIESCQKDENIMRSMQLFENVI*

>P1; HsapKChIP4

HsapKChIP4

MATVRHRPEALELLEAQSKFTKKELQILYRGFKNECPSGVVNEETFKEIYSQFFPQGDSTTYAHFLFNAFDTDHN

GAVSFEDFIKGLSILLRGTVQEKLNWAFNLYDINKDGYITKEEMLDIMKAIYDMMGKCTYPVLKEDAPRQHVETF

FQKMDKNKDGVVTIDEFIESCQKDENIMRSMQLFENVI*

>P1; OanaKChIP4

OanaKChIP4

MATVRHRPETLELLEAQSKFTKKELQILYRGFKNECPSGIVNEETFKEIYSQFFPQGDSTTYAHFLFNAFDTDHN

GSVSFEDFVMGLSILLRGTVQEKLNWAFNLYDINKDGYITKEEMLDIMKAIYDMMGKCTYPVLKEDAPRQHVETF

FQKMDKNKDGVVTIDEFIESCQKDENIMRSMQLFENVI*

>P1; EcabKChIP4

EcabKChIP4

MATVRHRPEALELLEAQSKFTKKELQILYRGFKNECPSGVVNEETFKEIYSQFFPQGDSTTYAHFLFNAFDTDHN

GAVSFEDFIKGLSILLRGTVQEKLNWAFNLYDINKDGYITKEEMLDIMKAIYDMMGKCTYPVLKEDAPRQHVETF

FQKMDKNKDGVVTIDEFIESCQKDENIMRSMQLFENVI*

>P1; TtruKChIP4

TtruKChIP4

MATVRHRPEALELLEAQSKFTKKELQILYRGFKNECPSGVVNEDTFKEIYSQFFPQGDSTTYAHFLFNAFDTDHN

GAVSFEDFIKGLSILLRGTVQEKLNWAFNLYDINKDGYITKEEMLDIMKAIYDMMGKCTYPVLKEDAPRQHVETF

FQKDKNKDGVVTIDEFIESCQKDENIMRSMQLFENVI*

>P1; VpacKChIP4

VpacKChIP4

MATVRHRPEALELLEAQSKFTKKELQILYRGFKNECPSGVVNEETFKEIYSQFFPQGDSTTYAHFLFNAFDTDHN

GAVSFEDFIKGLSILLRGTVQEKLNWAFNLYDINKDGYITKEEMLDIMKAIYDMMGKCTYPVLKEDAPRQHVETF

FQKMDKNKDGVVTIDEFIESCQKDENIMRSMNSTHWI*

>P1; PtroKChIP4

PtroKChIP4

MATVRHRPEALELLEAQSKFTKKELQILYRGFKNECPSGVVNEETFKEIYSQFFPQGDSTTYAHFLFNAFDTDHN

GAVSFEDFIKGLSILLRGTVQEKLNWAFNLYDINKDGYITKEEMLDIMKAIYDMMGKCTYPVLKEDAPRQHVETF

FQKMDKNKDGVVTIDEFIESCQKDENIMRSMQLFENVI*

>P1; TbelKChIP4

TbelKChIP4

MATVRHRPEALELLEAQSKFTKKELQILYRGFKNECPSGVVNEETFKEIYSQFFPQGDSTTYAHFLFNAFDTDHN

GAVSFEDFIKGLSILLRGTVQEKLNWAFNLYDINKDGYITKEEMLDIMKAIYDMMGKCTYPVLKEDAPRQHVETF

FQKMDKNKDGVVTIDEFIESCQKDENIMRSMQLFENVI*

>P1; PpygKChIP4

PpygKChIP4

MATVRHRPEALELLEAQSKFTKKELQILYRGFKNECPSGVVNEETFKEIYSQFFPQGDSTTYAHFLFNAFDTDHN

GAVSFEDFIKGLSILLRGTVQEKLNWAFNLYDINKDGYITKEEMLDIMKAIYDMMGKCTYPVLKEDAPRQHVETF

FQKMDKNKDGVVTIDEFIESCQKDENIMRSMQLFENVI*

>P1; DrerKChIP4

DrerKChIP4

LSAVRHRPEALEQLEAQTRFSRKELQILYRGFKNECPSGVVNEDTFKEIYAQFFPQGDASTYAHFLFNAFDTDHN

GSVSFEDFVMGLSILLRGSVQEKLNWAFNLYDINKDGYITKEEMLDIIKSIYDMMGKCTYPILKEETPRQHVEIF

FQKMDKNRDGVVTIDEFIDCCQNVMRSMQLFENVI*

>P1; TrubKChIP4

TrubKChIP4

LSAVRHRPEGLEQLESQTRFSRKELQILYRGFKNECPSGVVNEDTFKDIYAQFFPQGDASTYAHFLFNAFDTDHN

GNVSFEDFVMGLSILLRGTVQEKLNWAFNLYDINKDGYITKEEMLDIMKAIYDMMGKCTYPVLKEETPRQHVEVF

FQKMDKNKDGVVTIDEFIDCCQNDENIMRSMHLFENVL*

>P1; XtroKChIP4

XtroKChIP4

TATVRHRPEALELLEAQTKFTKKELQILYRGFKNECPSGIVNEETFKDIYAQFFPQGDASTYAHFLFNAFDTDHN

GSVSFEDFVIGLSTLLRGTIQEKLNWAFNLYDINKDGYITKEEMFDIMKSIYDMMGKCTYPLVREETPRQHVENF

FQKMDINKDGVVTIEEFIESCQKDENIMCSMQLFENVI*

>P1; TnigKChIP4

TnigKChIP4

LSAVRHRPEGLEQLESQTRFSRKELQILYRGFKNECPSGVVNEETFKDIYAQFFPQGDASTYAHFLFNAFDTDHN

GSVSFEDFVTGLSILLRGTVQEKLNWAFNLYDINKDGYITKEEMLDIMKAIYDMMGKCTYPVLKEETPRQHVEVF

FQKMDRNKDGVVTIDEFIDCCQNDENIMRSMHLFENVL*

>P1; OlatKChIP4

OlatKChIP4

LSTVRHRPEGLEQLEAQTRFSRKELQILYRGFKNECPSGVVNEETFKDIYSQFFPQGDASTYAHFLFNAFDTDHN

GSVSFEDFVMGLSILLRGTVQEKLIWAFNLYDINKDGYISKEEMLDIMKAIYDMMGKCTYPVLKEETPRQHVEIF

FQQKMDKNKDGVVTIDEFIDSCQNDENIMRSMHLFENVL*

>P1; TgutKChIP4

TgutKChIP4

MATVRHRPEALELLEAQSKFTKKELQILYRGFKNECPSGVVNEETFKEIYSQFFPQGDSTTYAHFLFNAFDTDHN

GSVSFEDFVMGLSILLRGTVQEKLNWAFNLYDINKDGYITKEEMLDIMKAIYDMMGKCTYPVLKEDAPRQHVETF

FQKMDKNKDGVVTIDEFIESCQKDENIMRSMQLFENVI*

>P1; TsyrKChIP4

TsyrKChIP4

MATVRHRPEALELLEAQSKFTKKELQILYRGFKNECPSGVVNEETFKEIYSQFFPQGDTTYAHFLFNALDTDHNV

SVIFDFIKGLSILLRGTVQEKLNWAFNLYDINKDGYITKEEMLDIMKAIYDMMGKCTYPVLKEDAPRQHVETFFQ

KMDKNKDGVVTIDEFIESCQKDENIMRSMQLFENVI*

>P1; GacuKChIP4

GacuKChIP4

LSTVRHRPEGLEQLEARTRFSRKELQILYRGFKNECPSGIVNEETFKDIYAQFFPQGESSTYAHFLFNAFDTDHN

GSLSFEDFVMGLSILLRGTIQEKLNWAFNLYDINKDGYITKEEMLDIMKAIYDMMGKCTYPILKEDTPRQHVEVF

FQKMDKNKDGVVTIDEFIDCCQNDENIMRSMHLFENVL*

>P1; TnigVILIP1

TnigVILIP1

MGKQNSKLTPEVMEDLVKNTEFNEHELKQWYKGFLKDCPTGRLNLEEFQQLYVKFFPYGDASKFAQHAFRTFDKN

GDGTIDFREFICALSITSRGSFEQKLNWAFNMYDLDGDGKITRVEMLEIIEAIYKMVGTVIMMKMNEDGLTPEQR

VDKIFSKMDKNNDDQISLDEFKEAAKSDPSIVLLLQCDMQK*

>P1; LafrVILIP1

LafrVILIP1

MGKQNSKLAPEVMEDLVKSTEFNEHELKQWYKGFLKDCPSGRLNLEEFQQLYVKFFPYGDASKFAQHAFRTFDKN

GDGTIDFREFICALSITSRGSFEQKLNWAFNMYDLDGDGKITRVEMLEIIEAIYKMVGTVIMMKMNEDGLTPEQR

VDKIFSKMDKNKDDQITLDEFKEAAKSDPSIVLLLQCDIQK*

>P1; PtroVILIP1

PtroVILIP1

MGKQNSKLAPEVMEDLVKSTEFNEHELKQWYKGFLKDCPSGRLNLEEFQQLYVKFFPYGDASKFAQHAFRTFDKN

GDGTIDFREFICALSITSRGSFEQKLNWAFNMYDLDGDGKITRVEMLEIIEAIYKMVGTVIMMKMNEDGLTPEQR

VDKIFSKMDKNKDDQITLDEFKEAAKSDPSIVLLLQCDIQK*

>P1; TgutVILIP1

TgutVILIP1

MGKQNSKLAPEVMEDLVKSTEFNEHELKQWYKGFLKDCPSGRLNLEEFQQLYVKFFPYGDASKFAQHAFRTFDKN

GDGTIDFREFICALSITSRGSFEQKLNWAFNMYDLDGDGKITRVEMLEIIEAIYKMVGTVIMMKMNEDGLTPEQR

VDKIFSKMDKNKDDQITLDEFKEAAKSDPSIVLLLQCDIQK*

>P1; DrerVILIP1

DrerVILIP1

MGKQNSKLTPEVMEDLVKNTEFNEHELKQWYKGFLKDCPTGRLNLDEFQQLYVKFFPYGDASKFAQHAFRTFDKN

GDGTIDFREFICALSITSRGSFEQKLNWAFNMYDLDGDGKITRVEMLEIIEAIYKMVGTVIMMKMNEDGLTPEQR

VDKIFSKMDKNNDDQISLEEFKEAAKSDPSIVLLLQCDLQK*

>P1; MmusVILIP1

MmusVILIP1

MGKQNSKLAPEVMEDLVKSTEFNEHELKQWYKGFLKDCPSGRLNLEEFQQLYVKFFPYGDASKFAQHAFRTFDKN

GDGTIDFREFICALSITSRGSFEQKLNWAFNMYDLDGDGKITRVEMLEIIEAIYKMVGTVIMMKMNEDGLTPEQR

VDKIFSKMDKNKDDQITLDEFKEAAKSDPSIVLLLQCDIQK*

>P1; CporVILIP1

CporVILIP1

MGKQNSKLAPEVMEDLVKSTEFNEHELKQWYKGFLKDCPSGRLNLEEFQQLYVKFFPYGDASKFAQHAFRTFDKN

GDGTIDFREFICALSITSRGSFEQKLNWAFNMYDLDGDGKITRVEMLEIIEAIYKMVGTVIMMKMNEDGLTPEQR

VDKIFSKMDKNKDDQITLDEFKEAAKSDPSIVLLLQCDIQK*

>P1; BtauVILIP1

BtauVILIP1

MGKQNSKLAPEVMEDLVKSTEFNEHELKQWYKGFLKDCPSGRLNLEEFQQLYVKFFPYGDASKFAQHAFRTFDKN

GDGTIDFREFICALSITSRGSFEQKLNWAFNMYDLDGDGKITRVEMLEIIEAIYKMVGTVIMMKMNEDGLTPEQR

VDKIFSKMDKNKDDQITLDEFKEAAKSDPSIVLLLQCDIQK*

>P1; RnorVILIP1

RnorVILIP1

MGKQNSKLAPEVMEDLVKSTEFNEHELKQWYKGFLKDCPSGRLNLEEFQQLYVKFFPYGDASKFAQHAFRTFDKN

GDGTIDFREFICALSITSRGSFEQKLNWAFNMYDLDGDGKITRVEMLEIIEAIYKMVGTVIMMKMNEDGLTPEQR

VDKIFSKMDKNKDDQITLDEFKEAAKSDPSIVLLLQCDIQK*

>P1; PcapVILIP1

PcapVILIP1

MGKQNSKLAPEVMEDLVKSTEFNEHELKQWYKGFLKDCPSGRLNLEEFQQLYVKFFPYGDASKFAQHAFRTFDKN

GDGTIDFREFICALSITSRGSFEQKLNWAFNMYDLDGDGKITRVEMLEIIEAIYKMVGTVIMMKMNEDALTPEQR

VDKIFSKMDKNKDDQITLDEFKEAAKSDPSIVLLLQCDIQK*

>P1; EtelVILIP1

EtelVILIP1

MGKQNSKLAPEVMEDLVKSTEFNEHELKQWYKGFLKDCPSGRLNLEEFQQLYVKFFPYGDASKFAQHAFRTFDKN

GDGTIDFREFICALSITSRGSFEQKLNWALNMYDLDGDGQITRVEMLEIIEAIYKMVGTVIMMKMNEDGLTPEQR

VDKIFSKMDKNKDDQITLDEFKEAAKSDPSIVLLLQCDIQK*

>P1; PvamVILIP1

PvamVILIP1

MGKQNSKLAPEVMEDLVKSTEFNEHELKQWYKGFLKDCPSGRLNLEEFQQLYVKFFPYGDASKFAQHAFRTFDKN

GDGTIDFREFICALSITSRGSFEQKLNWAFNMYDLDGDGKITRVEMLEIIEAIYKMVGTVIMMKMNEDGLTPEQR

VDKIFSKMDKNKDDQITLDEFKEAAKSDPSIVLLLQCDIQK*

>P1; TtruVILIP1

TtruVILIP1

MGKQNSKLAPEVMEDLVKSTEFNEHELKQWYKGFLKDCPSGRLNLEEFQQLYVKFFPYGDASKFAQHAFRTFDKN

GDGTIDFREFICALSITSRGSFEQKLNWAFNMYDLDGDGKITRVEMLEIIEAIYKMVGTVIMMKMNEDGLTPEQR

VDKIFSKMDKNKDDQITLDEFKEAAKSDPSIVLLLQCDIQK*

>P1; EcabVILIP1

EcabVILIP1

MGKQNSKLAPEVMEDLVKSTEFNEHELKQWYKGFLKDCPSGRLNLEEFQQLYVKFFPYGDASKFAQHAFRTFDKN

GDGTIDFREFICALSITSRGSFEQKLNWAFNMYDLDGDGKITRVEMLEIIEAIYKMVGTVIMMKMNEDGLTPEQR

VDKIFSKMDKNKDDQITLDEFKEAAKSDPSIVLLLQCDIQK*

>P1; TrubVILIP1

TrubVILIP1

MGKQNSKLTPEVMEDLVKNTEFNEHELKQWYKGFLKDCPTGRLNLDEFQQLYVKFFPYGDASKFAQHAFRTFDKN

GDGTIDFREFICALSITSRGSFEQKLNWAFNMYDLDGDGKITRVEMLEIIEAIYKMVGTVIMMKMNEDGLTPEQR

VDKIFSKMDKNNDDQISLEEFKEAAKSDPSIVLLLQCDLQK*

>P1; TsyrVILIP1

TsyrVILIP1

MGKQNSKLAPEVMEDLVKSTEFNEHELKQWYKGFLKDCPSGRLNLEEFQQLYVKFFPYGDASKFAQHAFRTFDKN

GDGTIDFREFICALSITSRGSFEQKLNWAFNMYDLDGDGKITRVEMLEIIEAIYKMVGTVIMMKMNEDGLTPEQR

VDKIFSKMDKNKDDQITLDEFKEAAKSDPSIVLLLQCDIQK*

>P1; GgalVILIP1

GgalVILIP1

MGKQNSKLAPEVMEDLVKSTEFNEHELKQWYKGFLKDCPSGRLNLEEFQQLYVKFFPYGDASKFAQHAFRTFDKN

GDGTIDFREFICALSITSRGSFEQKLNWAFNMYDLDGDGKITRVEMLEIIEAIYKMVGTVIMMKMNEDGLTPEQR

VDKIFSKMDKNKDDQITLDEFKEAAKSDPSIVLLLQCDIQK*

>P1; AcarVILIP1

AcarVILIP1

MGKQNSKLTPEVMEDLVKSTEFNEHELKQWYKGFLKDCPSGRLNLDEFQQLYVKFFPYGDASKFAQHAFRTFDKN

GDGTIDFREFICALSITSRGSFEQKLNWAFNMYDLDGDGKITRVEMLEIIEAIYKMVGTVIMMKMNEDGLTPEQR

VDKIFSKMDKNKDDQITLDEFKEAAKSDPSIVLLLQCDIQK*

>P1; OlatVILIP1

OlatVILIP1

MGKQNSKLTPEVMEDLVKNTEFNEHELKQWYKGFLKDCPSGRLNLDEFQQLYVKFFPYGDASKFAQHAFRTFDKN

GDGTIDFREFICALSITSRGSFEQKLNWAFNMYDLDGDGKITRVEMLEIIEAIYKMVGTVIMMKMNEDGLTPQQR

VDKIFSKMDKNNDDQISLDEFKEAAKSDPSIVLLLQCDLQK*

>P1; TbelVILIP1

TbelVILIP1

MGKQNSKLAPEVMEDLVKSTEFNEHELKQWYKGFLKDCPSGRLNLEEFQQLYVKFFPYGDASKFAQHAFRTFDKN

GDGTIDFREFICALSITSRGSFEQKLNWAFNMYDLDGDGKITRVEMLEIIEAIYKMVGTVIMMKMNEDGLTPEQR

VDKIFSKMDKNKDDQITLDEFKEAAKSDPSIVLLLQCDIQK*

>P1; XtroVILIP1

XtroVILIP1

MGKQNSKLAPEVMEDLVKSTEFNEHELKQWYKGFLKDCPSGRLNLDEFQQLYVKFFPYGDASKFAQHAFRTFDKN

GDGTIDFREFICALSITSRGSFEQKLNWAFNMYDLDGDGKITRVEMLEIIEAIYKMVGTVIMMKMNEDGLTPEQR

VDKIFSKMDKNKDDQITLDEFKEAAKSDPSIVLLLQCDIQK*

>P1; HsapVILIP1

HsapVILIP1

MGKQNSKLAPEVMEDLVKSTEFNEHELKQWYKGFLKDCPSGRLNLEEFQQLYVKFFPYGDASKFAQHAFRTFDKN

GDGTIDFREFICALSITSRGSFEQKLNWAFNMYDLDGDGKITRVEMLEIIEAIYKMVGTVIMMKMNEDGLTPEQR

VDKIFSKMDKNKDDQITLDEFKEAAKSDPSIVLLLQCDIQK*

>P1; OcunVILIP1

OcunVILIP1

MGKQNSKLAPEVMEDLVKSTEFNEHELKQWYKGFLKDCPSGRLNLEEFQQLYVKFFPYGDASKFAQHAFRTFDKN

GDGTIDFREFICALSITSRGSFEQKLNWAFNMYDLDGDGKITRVEMLEIIEAIYKMVGTVIMMKMNEDGLTPEQR

VDKIFSKMDKNKDDQITLDEFKEAAKSDPSIVLLLQCDIQK*

>P1; GacuVILIP1

GacuVILIP1

MGKQNSKLTPEVMEDLVKNTEFNEHELKQWYKGFLKDCPTGRLNLDEFQQLYVKFFPYGDASKFAQHAFRTFDKN

GDGTIDFREFICALSITSRGSFEQKLNWAFNMYDLDGDGKITRVEMLEIIEAIYKMVGTVIMMKMNEDGLTPQQR

VDKIFGKMDKNNDDQISLDEFKEAAKSDPSIVLLLQCDMQK*

>P1; PpygVILIP1

PpygVILIP1

MGKQNSKLAPEVMEDLVKSTEFNEHELKQWYKGFLKDCPSGRLNLEEFQQLYVKFFPYGDASKFAQHAFRTFDKN

GDGTIDFREFICALSITSRGSFEQKLNWAFNMYDLDGDGKITRVEMLEIIEAIYKMVGTVIMMKMNEDGLTPEQR

VDKIFSKMDKNKDDQITLDEFKEAAKSDPSIVLLLQCDIQK*

>P1; StriNEUROCALCIN

StriNEUROCALCIN

MGKQNSKLRPEVMQDLLESTDFTEHEIQEWYKGFLRDCPSGHLSMEEFKKIYGNFFPYGDASKFAEHVFRTFDAN

GDGTIDFREFIIALSVTSRGKLEQKLKWAFSMYDLDGNGYISKAEMLEIVQAIYKMVSSVMKMPEDESTPEKRTE

KIFRQMDTNRDGQPLPPKKSDGSCRTEIEGPNKSLLATSAQDRPWP*

>P1; MdomNEUROCALCIN

MdomNEUROCALCIN

MGKQNSKLRPEVMQDLLESTDFTEHEIQEWYKGFLRDCPSGHLSMEEFKKIYGNFFPYGDASKFAEHVFRTFDAN

GDGTIDFREFIIALSVTSRGKLEQKLKWAFSMYDLDGNGYISKAEMLEIVQAIYKMVSSVMKMPEDESTPEKRTE

KIFRQMDTNRDGKLSLEEFIRGAKSDPSIVRLLQCDPSSAGQF*

>P1; DrerNEUROCALCIN

DrerNEUROCALCIN

MGKQNSKLRPEVMQDLLESTDFTEHEIQEWYKGFLRDCPSGNLSMEEFKKIYGNFFPYGDASKFAEHVFRTFDAN

GDGTIDFREFIIALSVTSRGKLEQKLKWAFSMYDLDGNGYISKSEMLEIVQAIYKMVSSVMKMPEDESTPEKRTE

KIFRQMDTNRDGKLSLEEFIKGAKSDPSIVRLLQCDPSSAGQ*

>P1; GgalNEUROCALCIN

GgalNEUROCALCIN

MGKQNSKLRPEVMQDLLESTDFTEHEIQEWYKGFLRDCPSGHLSMEEFKKIYGNFFPYGDASKFAEHVFRTFDAN

GDGTIDFREFIIALSVTSRGKLEQKLKWAFSMYDLDGNGYISKSEMLEIVQAIYKMVSSVMKMPEDESTPEKRTE

KIFRQMDTNRDGKLSLEEFIRGAKSDPSIVRLLQCDPSSAGQF*

>P1; CfamNEUROCALCIN

CfamNEUROCALCIN

MGKQNSKLRPEVMQDLLESTDFTEHEIQEWYKGFLRDCPSGHLSMEEFKKIYGNFFPYGDASKFAEHVFRTFDAN

GDGTIDFREFIIALSVTSRGKLEQKLKWAFSMYDLDGNGYISKAEMLEIVQAIYKMVSSVMKMPEDESTPEKRTE

KIFRQMDTNRDGKLSLEEFIRGAKSDPSIVRLLQCDPSSAGQF*

>P1; EcabNEUROCALCIN

EcabNEUROCALCIN

MGKQNSKLRPEVMQDLLESTDFTEHEIQEWYKGFLRDCPSGHLSMEEFKKIYGNFFPYGDASKFAEHVFRTFDAN

GDGTIDFREFIIALSVTSRGKLEQKLKWAFSMYDLDGNGYISKAEMLEIVQAIYKMVSSVMKMPEDESTPEKRTE

KIFRQMDTNRDGKLSLEEFIRGAKSDPSIVRLLQCDPSSAGQF*

>P1; OlatNEUROCALCIN

OlatNEUROCALCIN

MGKQNSKLRPEVMQDLLESTDFTEHEIQEWYKGFLRDCPSGNLSMEEFKKIYGNFFPYGDASKFAEHVFRTFDAN

GDGTIDFREFIIALSVTSRGKLEQKLKWAFSMYDLDGNGYISKAEMLEIVQAIYKMVSSVMKMPEDESTPEKRTE

KIFRQMDTNRDGKLSLEEFIKGAKSDPSIVRLLQCDPSSAAWS*

>P1; ChofNEUROCALCIN

ChofNEUROCALCIN

MGKQNSKLRPEVMQDLLESTDFTEHEIQEWYKGFLRDCPSGHLSMEEFKKIYGNFFPYGDASKFAEHVFRTFDAN

GDGTIDFREFIIALSVTSRGKLEQKLKWAFSMYDLDGNGYISKAEMLEIVQAIYKMVSSVMKMPEDESTPEKRTE

KIFRQMDTNRDGYFLYQTLNKSCGARKETPEKLLLTQPCPWRPWPQ*

>P1; PtroNEUROCALCIN

PtroNEUROCALCIN

MGKQNSKLRPEVMQDLLESTDFTEHEIQEWYKGFLRDCPSGHLSMEEFKKIYGNFFPYGDASKFAEHVFRTFDAN

GDGTIDFREFIIALSVTSRGKLEQKLKWAFSMYDLDGNGYISKAEMLEIVQAIYKMVSSVMKMPEDESTPEKRTE

KIFRQMDTNRDGQLLPPRKQNRSCGARMKGTKKPLLAQTHPWDRPRL*

>P1; TbelNEUROCALCIN

TbelNEUROCALCIN

MGKQNSKLRPEVMQDLLESTDFTEHEIQEWYKGFLRDCPSGHLSMEEFKKIYGNFFPYGDASKFAEHVFRTFDAN

GDGTIDFREFIIALSVTSRGKLEQKLKWAFSMYDLDGNGYISKAEMLEIVQAIYKMVSSVMKMPEDESTPEKRTE

KIFRQMDTNRDDELLPSEREKERERACNGERKQASRKPLPALTLHTPRPF*

>P1; MmurNEUROCALCIN

MmurNEUROCALCIN

MGKQNSKLRPEVMQDLLESTDFTEHEIQEWYKGFLRDCPSGHLSMEEFKKIYGNFFPYGDASKFAEHVFRTFDAN

GDGTIDFREFIIALSVTSRGKLEQKLKWAFSMYDLDGNGYISKAEMLEIVQAIYKMVSSVMKMPEDESTPEKRTE

KIFRQMDTNRDGYLLLPRKDRSCGRIEGLKKAWLAQTSPQDRPRA*

>P1; MmulNEUROCALCIN

MmulNEUROCALCIN

MGKQNSKLRPEVMQDLLESTDFTEHEIQEWYKGFLRDCPSGHLSMEEFKKIYGNFFPYGDASKFAEHVFRTFDAN

GDGTIDFREFIIALSVTSRGKLEQKLKWAFSMYDLDGNGYISKAEMLEIVQAIYKMVSSVMKMPEDESTPEKRTE

KIFRQMDTNRDGKLSLEEFIRGAKSDPSIVRLLQCDPSSAGQF*

>P1; AcarNEUROCALCIN

AcarNEUROCALCIN

MGKQNSKLRPEVMQDLLESTDFTEHEIQEWYKGFLRDCPSGHLSMEEFKKIYGNFFPYGDASKFAEHVFRTFDAN

GDGTIDFREFIIALSVTSRGKLEQKLKWAFSMYDLDGNGYISKAEMLEIVQAIYKMVSSVMKMPEDESTPEKRTE

KIFRQMDTNRDGKLSLEEFIRGAKSDPSIVRLLQCDPSSAGQF*

>P1; HsapNEUROCALCIN

HsapNEUROCALCIN

MGKQNSKLRPEVMQDLLESTDFTEHEIQEWYKGFLRDCPSGHLSMEEFKKIYGNFFPYGDASKFAEHVFRTFDAN

GDGTIDFREFIIALSVTSRGKLEQKLKWAFSMYDLDGNGYISKAEMLEIVQAIYKMVSSVMKMPEDESTPEKRTE

KIFRQMDTNRDGQLLPPRKQNGSCGARMKGTKKPLLAQTHPWDRPRL*

>P1; OcunNEUROCALCIN

OcunNEUROCALCIN

MGKQNSKLRPEVMQDLLESTDFTEHEIQEWYKGFLRDCPSGHLSMEEFKKIYGNFFPYGDASKFAEHVFRTFDAN

GDGTIDFREFIIALSVTSRGKLEQKLKWAFSMYDLDGNGYISKAEMLEIVQAIYKMVSSVMKMPEDESTPEKRTE

KIFRQMDTNRDGHLLPPRKQDACGTGLGGPKKPLLAEPSCRDRPRS*

>P1; TrubNEUROCALCIN

TrubNEUROCALCIN

MGKQNSKLRPEVMQDLLESTDFTEHEIQEWYKGFLRDCPSGHLSMEEFKKIYGNFFPYGDASKFAEHVFRTFDAN

GDGTIDFREFIIALSVTSRGKLEQKLKWAFSMYDLDGNGYISKAEMLEIVQAIYKMVSSVMKMPEDESTPEKRTE

KIFRQMDTNRDGKLSLEEFIKGAKSDPSIVRLLQCDPSSAGQF*

>P1; DordNEUROCALCIN

DordNEUROCALCIN

MGKQNSKLRPEVMQDLLESTDFTEHEIQEWYKGFLRDCPSGHLSMEEFKKIYGNFFPYGDASKFAEHVFRTFDAN

GDGTIDFREFIIALSVTSRGKLEQKLKWAFSMYDLDGNGYISKAEMLEIVQAIYKMVSSVMKMPEDESTPEKRTE

KIFRQMDTNRDGHSLPPSPKDRSCEAEGKGLKKPLLAQPSPQERPW*

>P1; CporNEUROCALCIN

CporNEUROCALCIN

MGKQNSKLRPEVMQDLLESTDFTEHEIQEWYKGFLRDCPSGHLSMEEFKKIYGNFFPYGDASKFAEHVFRTFDAN

GDGTIDFREFIIALSVTSRGKLEQKLKWAFSMYDLDGNGYISKAEMLEIVQAIYKMVSSVMKMPEDESTPEKRTE

KIFRQMDTNRDGKLSLEEFIRGAKSDPSIVRLLQCDPSSAGQF*

>P1; MmusNEUROCALCIN

MmusNEUROCALCIN

MGKQNSKLRPEVMQDLLESTDFTEHEIQEWYKGFLRDCPSGHLSMEEFKKIYGNFFPYGDASKFAEHVFRTFDAN

GDGTIDFREFIIALSVTSRGKLEQKLKWAFSMYDLDGNGYISKAEMLEIVQAIYKMVSSVMKMPEDESTPEKRTE

KIFRQMDTNRDGKLSLEEFIRGAKSDPSIVRLLQCDPSSAGQF*

>P1; GacuNEUROCALCIN

GacuNEUROCALCIN

MGKQNSKLRPEVMQDLLESTDFTEHEIQEWYKGFLRDCPSGNLSMEEFKKIYGNFFPYGDASKFAEHVFRTFDAN

GDGTIDFREFIIALSVTSRGKLEQKLKWAFSMYDLDGNGYISKAEMLEIVQAIYKMVSSVMKMPEDESTPEKRTE

KIFRQMDTNRDGKLSLEEFIKGAKSDPSIVRLLQCDPSSAGQF*

>P1; PpygNEUROCALCIN

PpygNEUROCALCIN

MGKQNSKLRPEVMQDLLESTDFTEHEIQEWYKGFLRDCPSGHLSMEEFKKIYGNFFPYGDASKFAEHVFRTFDAN

GDGTIDFREFIIALSVTSRGKLEQKLKWAFSMYDLDGNGYISKAEMLEIVQAIYKMVSSVMKMPEDESTPEKRTE

KIFRQMDTNRDGKLSLEEFIRGAKSDPSIVRLLQCDPSSAGQF*

>P1; XtroNEUROCALCIN

XtroNEUROCALCIN

MGKQNSKLRPEVMQDLLESTDFTEHEIQEWYKGFLRDCPSGHLTMEEFKKIYGNFFPYGDASKFAEHVFRTFDAN

GDGTIDFREFIIALSVTSRGKLEQKLKWAFSMYDLDGNGYISKAEMLEIVQAIYKMVSSVMKMPEDESTPEKRTE

KIFRQMDTNRDGKLSLEEFIRGAKSDPSIVRLLQCDPSSAGQF*

>P1; TnigNEUROCALCIN

TnigNEUROCALCIN

MGKQNSKLRPEVMQDLLESTDFTEHEIQEWYKGFLRDCPSGHLSMEEFKKIYGNFFPYGDASKFAEHVFRTFDAN

GDGTIDFREFIIALSVTSRGKLEQKLKWAFSMYDLDGNGYISKAEMLEIVQAIYKMVSSVMKMPEDESTPEKRTE

KIFRQMDTNRDGKLSLEEFIKGAKSDPSIVRLLQCDPSSAGQF1*
